# GNL3 SUMOylation is essential for DNA double-strand break repair by homologous recombination

**DOI:** 10.1038/s41467-026-77013-1

**Published:** 2026-08-24

**Authors:** Yunhan Yang, Yanping Li, Roselyn S. Dai, Canping Chen, Keith Zientek, Ashok P. Reddy, Zheng Xia, Rosalie C. Sears, Xiao-Xin Sun, Mu-Shui Dai

**Affiliations:** 1 Department of Molecular & Medical Genetics, School of Medicine, Oregon Health & Science University, Portland, OR USA; 2 Department of Biomedical Engineering, Oregon Health & Science University, Portland, OR USA; 3 OHSU Proteomics Shared Resource, Oregon Health & Science University, Portland, OR USA; 4 Center for Biomedical Data Science, Oregon Health & Science University, Portland, OR USA; 5 OHSU Knight Cancer Institute, Oregon Health & Science University, Portland, OR USA; 6 Brenden-Colson Center for Pancreatic Care, Oregon Health & Science University, Portland, OR USA

**Keywords:** Homologous recombination, Sumoylation, Cancer therapy

## Abstract

DNA double-strand break repair via homologous recombination is critical for maintaining genomic integrity and requires proper DNA end resection. Here, we identify GNL3, a nucleolar GTP-binding protein, as a key regulator of homologous recombination in human cells via SUMOylation-dependent control of DNA end resection. Expression of wild-type GNL3, but not the SUMO-defective K196R mutant, abolished DNA damage induced by knockdown of endogenous GNL3. GNL3 interacts with the BLM-DNA2 helicase-nuclease complex, promoting DNA end resection and subsequent RPA and RAD51 loading. This interaction requires SUMOylation and SUMO-interacting motifs in both proteins. We further show that USP36 acts as a SUMO ligase for GNL3, while SENP3 deSUMOylates GNL3. Breast cancer-derived GNL3 variants disrupting its SUMOylation or SUMO-interacting motif fail to interact with the BLM-DNA2 complex. GNL3 depletion sensitizes homologous recombination-proficient breast cancer cells to etoposide and Olaparib treatment, highlighting GNL3 and its SUMOylation as potential therapeutic targets in cancer.

## Abstract (web summary)

GNL3 is identified as a key regulator of DNA repair, where its SUMOylation enables interaction with the BLM-DNA2 complex to promote DNA end resection and homologous recombination. Targeting this pathway sensitizes cancer cells to therapy.

## Introduction

DNA double-strand break (DSB) induced by both endogenous and exogenous DNA damage agents, such as replication stress, irradiation (IR), and toxic chemicals, represents the most detrimental form of genomic lesions in cells^1^. Cells develop a fundamental and sophisticated process called DNA damage response (DDR) to sense and repair DNA breaks to ensure genomic integrity^1,2^. Eukaryotic cells utilize two major pathways to repair DSBs: non-homologous end joining (NHEJ), which directly rejoins the DSB ends but is error-prone^3^, whereas homologous recombination (HR) provides high-fidelity repair by using a sister chromatid as the template during the S and G2 phases of the cell cycle and is also the primary mechanism for repairing replication-associated DSBs^4,5^. A third backup mechanism called microhomology-mediated end-joining (MMEJ) repairs DSBs by DNA polymerase theta (Polθ) during mitosis or when HR and NHEJ are compromised and is inherently mutagenic^6^. Unrepaired or incorrectly repaired DSBs lead to genomic instability, cell death and various diseases, including cancer^1^.

DNA end resection is an essential process in HR and determines the choice of HR over NHEJ^5,7,8^. The MRE11-RAD50-NBS1 (MRN) complex senses DSBs and recruits the ATM kinase to phosphorylate downstream effector proteins to amplify and orchestrate the cellular response to DSBs^8–10^. MRE11 initiates DNA end resection by cleaving the 5’ strand of the DSB ends with the cooperation of its phosphorylated CtIP cofactor, generating a short stretch of 3’ single-stranded DNA (ssDNA), which serves as a docking site for the long-range resection exonuclease 1 (Exo1) or the DNA helicase BLM and the DNA replication helicase/nuclease 2 (DNA2) complex^7,8^. The helicases and nucleases work to unwind the DNA double helix and digest the 5’ DNA strand to generate a long stretch of 3’ ssDNA^7,8^. The resulting ssDNA is rapidly coated and stabilized by the replication protein A (RPA) complex, which is then replaced by the RAD51 recombinase to form a nucleofilament, a step that requires the BRCA1-PALB2-BRCA2 complex^5,10,11^. The RAD51-ssDNA nucleoprotein filaments conduct a homology search and catalyze DNA strand exchange for high-fidelity repair of DSBs by HR^12^.

While defects in the HR pathway result in genomic instability and tumorigenesis, cancers with HR deficiency are synthetic lethal to treatment with poly(ADP-Ribose) polymerase inhibitors (PARPi), including tumors with BRCA1 mutations or with somatic mutations in other DDR and repair pathway genes in the absence of BRCA1 mutations (called “BRCAness”)^13^. Yet, the majority of cancers are HR-proficient and less sensitive to PARPi. HR-deficient cancers, although initially effective, often become resistant to PARPi due to BRCA1/2 reversion mutations or other adaptive mechanisms that restore the HR pathway^14–16^. Thus, there is an unmet need to identify new HR pathway targets to clinically re-sensitize cancers to PARPi treatment.

The DDR pathways are tightly regulated by post-translational modifications, including SUMOylation, the addition of small ubiquitin-like modifiers (SUMOs) to proteins. Mammals express three main SUMO isoforms: SUMO2 and SUMO3 are 97% identical, and each shares 45% sequence identity with SUMO1^17,18^. SUMOylation is ATP-dependent and occurs through sequential reactions involving a heterodimeric SUMO-activating enzyme SAE1/SAE2 (E1), a single SUMO-conjugating enzyme Ubc9 (E2) and one of a few SUMO ligases (E3)^17,18^. The SUMO acceptor lysine (Lys, K) is often present within a conserved ΨKxE motif, where Ψ is a large hydrophobic amino acid, and x is any amino acid^19^. Various DDR pathway proteins^20–23^ are regulated by SUMOylation, and SUMOylated proteins are enriched at local sites of DNA damage^24,25^. For example, SUMOylation of MDC1 upon DNA damage is critical for HR by promoting its RNF4-mediated ubiquitination, degradation and removal from DNA damage sites^26–29^. MRE11 requires SUMOylation to prevent its ubiquitination and degradation and function in DNA end resection^30^. BLM SUMOylation at K317 and K331 is necessary for its interaction with RAD51, RAD51 accumulation at stalled DNA forks^31^, and HR repair^32,33^. Other DDR proteins subjected to SUMO regulation include BRCA1^27–29^, BARD1^34,35^, RAD18^35^, RPA1^36^, RAD51^37–39^, RAD52^40–43^, Ku70^44^, and the SUMO-targeted ubiquitin ligase (STUBL) RNF4^27–29,45^, playing roles in both HR and NHEJ^27–29,34,46–50^.

GNL3, also called nucleostemin, is a nucleolar GTP-binding protein originally identified in neuronal stem cells and is essential for stem cell and cancer cell proliferation by regulating cell cycle progression^51^. High level expression of GNL3 has been observed in various cancers and cancer stem cells^52–56^. Its role in cell cycle regulation is closely linked to p53. GNL3 depletion induces p53 activation by inhibiting MDM2 through ribosomal proteins; additionally, GNL3 can directly bind to and inhibit MDM2 in the nucleoplasm, thereby promoting p53 activation during mitosis or upon ribosomal stress induced by perturbation of ribosome biogenesis^57–59^. However, GNL3 remains indispensable for the proliferation of p53-null cancer cells or mouse embryonic fibroblasts^60–63^. This p53-independent role is related to GNL3’s role in pre-rRNA processing and 60S ribosome biogenesis^64,65^. Consistently, GNL3 associates with pre-60S ribosomal particles during early nucleolar maturation steps and remains bound until their transition to nucleoplasm maturation stages^66^. Interestingly, GNL3 has also been implicated in genome maintenance. It participates in replication stress-induced DNA damage response and HR repair through interaction with RAD51^61,67,68^, and contributes to telomere maintenance by recruiting promyelocytic leukemia protein (PML) to SUMOylated telomeric repeat binding factor 1 (TRF1)^69^. These findings suggest that GNL3 coordinates multiple cellular processes, with its role in genome maintenance potentially preceding its function in ribosome biogenesis^62^. In this context, GNL3L has been proposed as the closest invertebrate homolog primarily dedicated to ribosome biogenesis^62,70^. Despite these advances, how GNL3 is regulated in cells and how it is involved in HR repair remain poorly understood.

Here, we report that GNL3 is regulated by SUMOylation, and this SUMOylation plays a key role in HR repair. DNA damage promotes GNL3 SUMOylation and its interaction with the BLM-DNA2 complex. We showed that GNL3 contains a SUMO-interacting motif (SIM) and its interaction with BLM requires SUMO-SIM interactions between the two proteins. We further discovered that GNL3 is SUMOylated by USP36, a nucleolar deubiquitinase and SUMO ligase dual-function enzyme^71–75^, and deSUMOylated by the SUMO protease SENP3. Several breast cancer-derived GNL3 variants abolish its SUMO regulation in response to DSBs. Knockdown of GNL3 sensitizes HR-proficient TNBC cells to genotoxic agents and PARPi. Thus, USP36 and SENP3-regulated GNL3 SUMOylation is critical for DNA end resection during HR repair and GNL3 and its SUMOylation may represent important cancer therapeutic targets.

## Results

### GNL3 is SUMOylated in cells in response to DNA damage

**Fig. 1:**
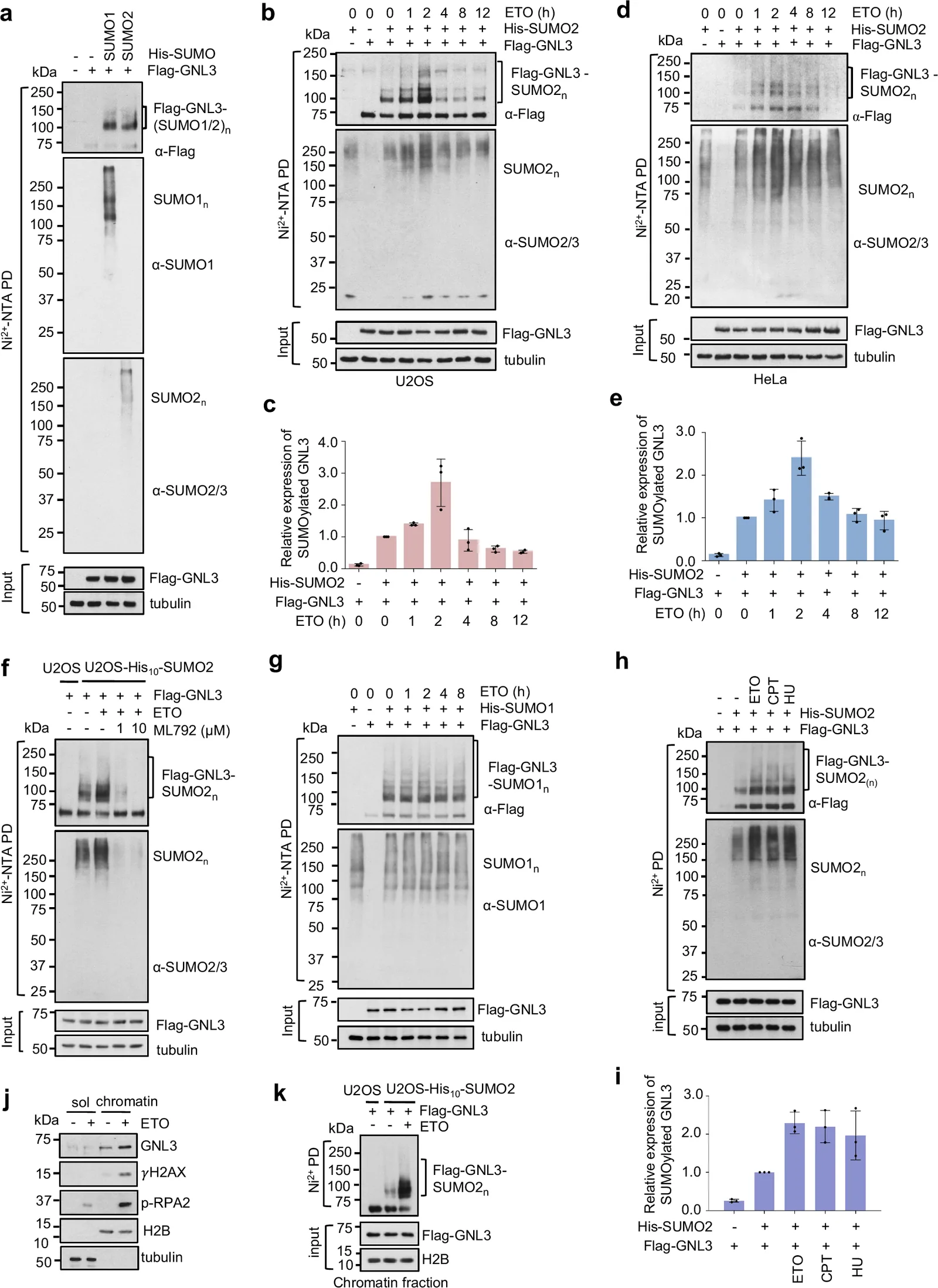
DNA damage induces GNL3 SUMOylation. **a** H1299 cells transfected with the indicated plasmids were subjected to Ni^2+^-NTA pulldown (PD) under denaturing conditions to detect GNL3 SUMOylation. Shown are representative results from four independent experiments. **b–e** U2OS (**b**, **c**) and HeLa (**d**, **e**) cells transfected with the indicated plasmids were treated with 20 μM ETO for the indicated times or control DMSO and assayed for GNL3 SUMOylation by Ni^2+^-NTA PD. Quantification of SUMOylated GNL3 relative to total GNL3 from three independent experiments is shown in (**c**) and (**e**). Data were presented as mean ± SD. **f** U2OS cells stably expressing His_10_-SUMO2 were transfected with Flag-GNL3, treated with 20 μM ETO and SUMO E1 inhibitor ML792, and assayed for GNL3 SUMOylation. **g** U2OS cells transfected with the indicated plasmids were treated with DMSO or with 20 μM ETO for the indicated times and assayed for GNL3 SUMOylation. Representative results from three independent experiments. Quantification of SUMOylated GNL3 relative to total GNL3 is shown in Supplementary Fig. S1c. **h**, **i** U2OS cells stably expressing His_10_-SUMO2 were treated with control DMSO, 20 μM ETO or 2 μM CPT for 2 h or 10 mM HU for 4 h and then assayed for GNL3 SUMOylation. Quantification from three independent experiments is shown in (**i**). Data were presented as mean ± SD. **j** U2OS cells treated with control or 20 μM ETO for 2 h were fractionated into soluble and chromatin fractions and assayed by IB. **k** Chromatin fractions isolated from the Flag-GNL3 transfected cells treated with DMSO or 20 μM ETO for 2 h were assayed for GNL3 SUMOylation. **f**, **j**, **k**, n= two independent experiments.

To understand how GNL3’s role in HR is regulated, we sought to test if GNL3 is regulated by SUMOylation, given that SUMOylation plays a critical role in DDR^23,76^. Indeed, GNL3 can be modified by both SUMO1 and SUMO2 (Fig. 1a and Supplementary Fig. S1a) and overexpression of Ubc9 markedly increased GNL3 SUMOylation, whereas dominant-negative Ubc9 mutant suppressed GNL3 SUMOylation in cells (Supplementary Fig. S1b). We next examined whether GNL3 can be SUMOylated in response to DNA damage. We found that treatment of etoposide (ETO), a topoisomerase 2 inhibitor that induces DSBs^77,78^, markedly induced GNL3 SUMOylation by SUMO2 in U2OS (Fig. 1b, c) and HeLa (Fig. 1d, e) cells, peaking at 2 hours following the ETO treatment. GNL3 SUMOylation induced by ETO treatment is completely blocked by the SUMO activating enzyme (E1) inhibitor ML792 (Fig. 1f). Interestingly, GNL3 SUMOylation by SUMO1 remained unchanged upon ETO treatment (Fig. 1g and Supplementary Fig. S1c). GNL3 SUMOylation by SUMO2 was also increased in cells treated with the topoisomerase 1 inhibitor camptothecin (CPT), the replication stress inducer hydroxyurea (HU) (Fig. 1h, i), and X-ray irradiation (Supplementary Fig. S1d, e). These data reveal that DNA damage promotes GNL3 SUMOylation by SUMO2. Chromatin fractionation assays showed that chromatin-associated GNL3 is significantly increased upon ETO treatment (Fig. 1j) and GNL3 SUMOylation in the chromatin fraction is significantly induced by the ETO treatment (Fig. 1k). Together, these results suggest that GNL3 modification by SUMO2 on chromatin may play a role in DNA damage response and repair.

### GNL3 SUMOylation at K196 is required for DSB repair and cell viability

**Fig. 2:**
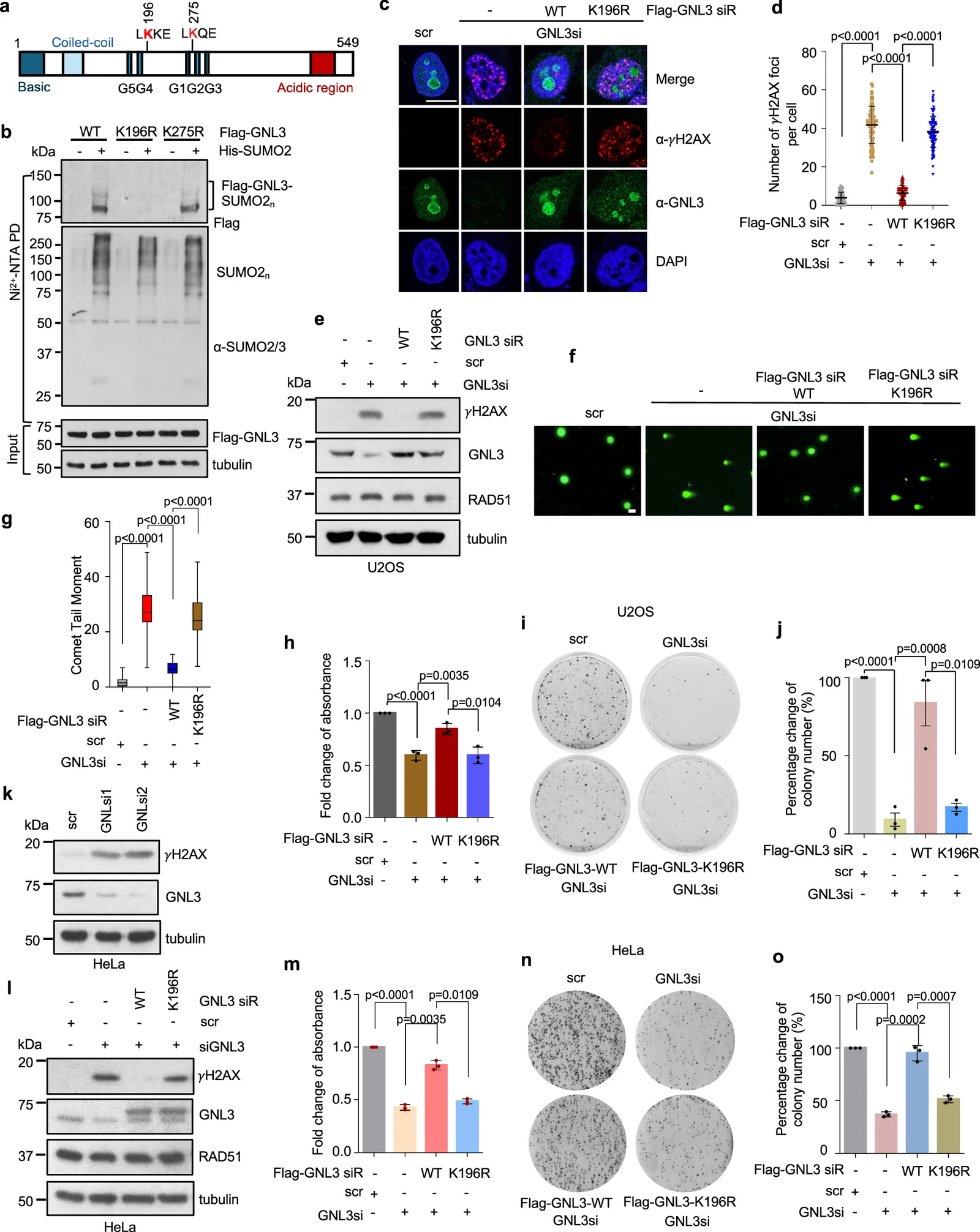
GNL3 SUMOylation at K196 is essential for DDR. **a** Diagram of GNL3 protein showing the putative SUMO sites and its associated basic, coiled-coil, G1-G5 GTP-binding motifs, and acidic region. **b** H1299 cells transfected with the indicated plasmids were assayed for GNL3 SUMOylation. *n* = 4 independent experiments. **c**, **d** IF staining of U2OS cells transfected with scrambled (scr) or GNL3 siRNA together with siRNA-resistant (siR) Flag-GNL3 (WT or K196R). Shown are representative confocal images (**c**) and the quantification (**d**) of *n* = 100 cells per condition from three independent experiments. **e** U2OS cells transfected with scr or GNL3 siRNA together with siRNA-resistant GNL3 (WT or K196R) were assayed by IB. **f**, **g** Comet assays in U2OS cells transfected with scr or GNL3 siRNA together with siRNA-resistant Flag-GNL3 (WT or K196R). Shown are representative images (**f**) and the quantification (**g**) of *n* = 100 cells per condition from three independent experiments. The centerline of the boxplots represents the median value (50th percentile), and the box encapsulates the interquartile range. The whiskers extend to show the rest of the distribution. **h–j** U2OS cells transfected with scr or GNL3 siRNA together with siRNA-resistant Flag-GNL3 (WT or K196R) were assayed by MTT cell viability (h) and colony formation assays (i)(j). Representative images (i) and quantification from three biological replicates (h)(j) are shown. **k** HeLa cells transfected with scr or two different GNL3 siRNAs were assayed by IB. **l** HeLa cells transfected with scr or GNL3 siRNA together with siRNA-resistant Flag-GNL3 (WT or K196R) were assayed by IB. **m–o** HeLa cells transfected with scr or GNL3 siRNA together with siRNA-resistant Flag-GNL3 (WT or K196R) were assayed by MTT cell viability (m) and colony formation (**n**)(**o**) assays. Representative images (n) and quantification (**m**, **o**) from three biological replicates are shown. Scale bars (**c**, **f**), 10 μm. The representative results (**e, k, l**) were from *n* = 3 independent experiments. Error bars (**d**, **h**, **j**, **m**, **o**) denote mean ± SD. Significance (**d**, **g**, **h**, **j**, **m**, **o**) was determined by an unpaired two-tailed Student’s *t*test.

To define the SUMOylation sites in GNL3 protein, we mutated the two consensus SUMO lysine (K) residues predicted by the GPS-SUMO tool^79^, K196 and K275 (Fig. 2a), to Arg (R) and examined their SUMOylation in cells. As shown in Fig. 2b, mutating K196, but not K275, largely abolished GNL3 SUMOylation, suggesting that GNL3 is SUMOylated at K196. To examine whether GNL3 SUMOylation is critical for its role in HR, we performed knockdown and rescue experiments. Consistent with previous studies^55,61^, knockdown of GNL3 markedly induced DDR as shown by the increased levels and foci formation of phosphorylated H2AX (γH2AX) in U2OS cells (Fig. 2c–e). Interestingly, ectopic expression of siRNA-resistant wild-type (WT) GNL3, but not the K196R mutant, abolished the induction of γH2AX foci (Fig. 2c, d) and levels (Fig. 2e) as well as the levels of phospho-RPA2 (Supplementary Fig. S2a) induced by knockdown of endogenous GNL3. ETO treatment induced similar protein levels and foci formation of γH2AX regardless of GNL3 expression (Supplementary Fig. S2a–c), indicating that ETO- induced DNA damage overrides the effects of GNL3 knockdown. Time-course studies of cells treated with ETO followed by washout showed that knockdown of GNL3 significantly impaired the clearance of γH2AX foci following ETO removal (Supplementary Fig. S2d, e), highlighting a critical role for GNL3 in DNA damage repair. Consistently, ectopic expression of WT GNL3, but not the K196R mutant, abolished the induction of p53 levels in response to knockdown of endogenous GNL3 (Supplementary Fig. S2f). Additionally, knockdown of GNL3 markedly induced DNA damage that can be abolished by ectopic expression of WT GNL3, but not the K196R mutant, as determined by Comet assays (Fig. 2f, g), but not in the presence of ETO treatment (Supplementary Fig. S2g, h). Cell viability (Fig. 2h) and colony formation (Fig. 2i, j) assays reveal that knockdown of GNL3 significantly inhibited U2OS cell growth, which is rescued by expression of WT GNL3, but not the K196R mutant. U2OS cells are proficient in telomerase-independent, recombination-based telomere maintenance, known as alternative lengthening of telomeres (ALT)^80–82^. To examine whether the above-observed role for GNL3 in HR depends on ALT, we performed similar GNL3 knockdown and rescue experiments in telomerase-positive, ALT-deficient HeLa cells^83^. Knockdown of GNL3 using two different siRNAs significantly increased γH2AX levels (Fig. 2k). Consistently, expression of WT GNL3, but not the K196R mutant, also abolished the induction of γH2AX and phospho-RPA2 induced by knockdown of endogenous GNL3 in HeLa cells (Fig. 2l & Supplementary Fig. S2i). Moreover, GNL3 knockdown significantly inhibited HeLa cell growth, which was rescued by expression of siRNA-resistant WT GNL3, but not the K196R mutant, as determined by cell viability (Fig. 2m) and colony formation (Fig. 2n, o) assays. Finally, DR-GFP reporter assays revealed that GNL3 knockdown markedly reduced HR efficiency (Supplementary Fig. S2j). Together, these results suggest that GNL3 SUMOylation is critical for DSB repair via HR and for cell viability independently of ALT.

### GNL3 SUMOylation is critical for its interaction with γH2AX and RAD51

**Fig. 3:**
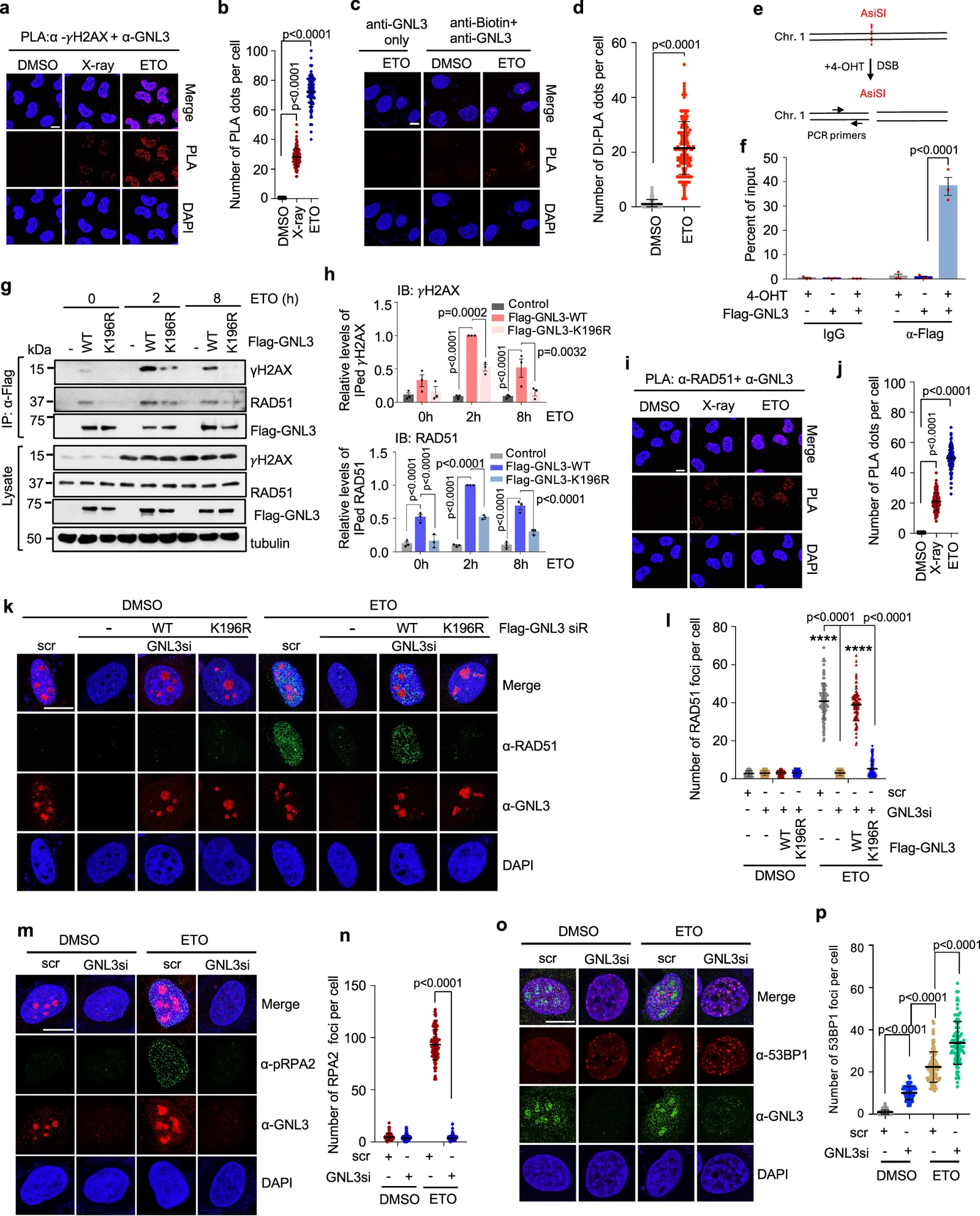
GNL3 SUMOylation is required for interaction with RAD51 and γH2AX and the RAD51 and RPA foci formation in response to DSBs. **a**, **b** U2OS cells treated with 10 Gy X-ray irradiation or 20 μM ETO for 2 hours were assayed by PLA. **c**, **d** U2OS cells treated with 20 μM ETO for 2 hours were assayed by DI-PLA. **e** Diagram of a region in chromosome 1 subjected to *Asi*SI enzyme digestion to generate DSBs upon 4-OHT treatment in ER-*Asi*SI U2OS cells. Primers for PCR amplification are indicated. **f** ChIP-qPCR detection of immunoprecipitated DNA in ER-*Asi*SI U2OS cells transfected with control or Flag-GNL3 plasmid. *n* = 3 independent experiments. **g**, **h** U2OS cells transfected with the indicated plasmids were treated with 20 μM ETO and assayed by co-IP. Quantification of immunoprecipitated γH2AX and RAD51 from three independent experiments is shown in (**h**). **i**, **j** U2OS cells treated with 10 Gy X-ray irradiation or 20 μM ETO for 2 h were assayed by PLA. **k**, **l** U2OS cells transfected with scr or GNL3 siRNA together with siRNA-resistant Flag-GNL3 (WT or K196R) were treated with DMSO or 20 μM ETO and assayed for RAD51 foci formation by IF. **m**, **n** U2OS cells transfected with scr or GNL3 siRNA were treated with DMSO or 20 μM ETO and assayed for RPA foci formation by IF. **o**, **p** U2OS cells transfected with scr or GNL3 siRNA were treated with DMSO or 20 μM ETO for 2 h and assayed for 53BP1 foci formation by IF. Scale bars, 10 μm. Representative confocal images (**a**, **c**, **i**, **k**, **m**, **o**) and the quantification of *n* = 100 (**b**, **j**, **l**, **n**, **p**) and *n* = 150 (**d**) cells per condition from three independent experiments are shown. Error bars denote mean ± SD. Significance was determined by unpaired two-tailed *t*testing (**b**, **d**, **j**) and two-way ANOVA, Tukey’s multiple comparisons test (**f**, **h**, **l**, **n**, **p**). ****P < 0.0001, compared to the respective DMSO-treated group.

To determine whether GNL3 localizes to DNA damage sites, we first examined its interaction with γH2AX, an early DNA damage marker that recruits DDR proteins to damage sites. We performed proximity ligation assays (PLAs) and observed that treatment of cells with either ETO or X-rays markedly induced the interaction between GNL3 and γH2AX (Fig. 3a, b). We then examined whether GNL3 directly associates with DNA breaks using a DNA damage in situ proximity ligation assay (DI-PLA)^84^, wherein DSBs are blunt-ended and ligated to a biotinylated DNA probe, followed by PLA between biotin and GNL3. As shown in Fig. 3c, d, DI-PLA revealed a robust increase in the association between GNL3 and ETO-induced DSBs. Furthermore, we performed chromatin immunoprecipitation (ChIP) assays using ER-*Asi*SI-U2OS cells^85,86^, in which DSBs can be induced by the addition of 4-hydroxytamoxifen (4-OHT), triggering translocation of *Asi*SI enzyme from the cytoplasm to the nucleus to cleave DNA to induce DSBs (Fig. 3e and Supplementary Fig. S3a). GNL3 binding to a chromosome 1 region was specifically detected only when DSBs were generated by 4-OHT-induced *Asi*SI activation (Fig. 3f and Supplementary Fig. S3b), demonstrating that GNL3 associates with DSBs generated by site-specific cleavage. Notably, high-resolution IF showed that nucleoplasmic GNL3 largely co-localized with γH2AX foci in cells treated with ETO (Supplementary Fig. S3c, d). Together, these results reveal that GNL3 directly associates with DNA damage sites. The interaction between GNL3 and γH2AX was also shown by co-immunoprecipitation (co-IP) assays, peaking at 2 hours after ETO treatment (Fig. 3g, h). Interestingly, abolishing GNL3 SUMOylation by mutating K196 markedly reduced its interaction with γH2AX in U2OS cells (Fig. 3g, h and Supplementary Fig. S3e). Similar results were also shown in HeLa cells (Supplementary Fig. S3f). These results suggest that GNL3 is recruited to the DNA damage site dependently on its SUMOylation. It has previously been shown that GNL3 interacts with RAD51, the recombinase essential for HR repair^61^. Indeed, PLA assays showed that the interaction between GNL3 and RAD51 is also markedly increased following the treatment with either X-rays or ETO (Fig. 3i, j). Co-IP assays also showed that the GNL3-RAD51 interaction peaked at 2 h after ETO treatment, which is markedly impaired by mutating K196 (Fig. 3g, h and Supplementary Fig. S3e, f). Together, these data suggest that GNL3 SUMOylation is critical for its recruitment to the sites of DNA damage and interaction with RAD51.

### GNL3 SUMOylation is required for RAD51 and RPA2 foci formation in response to DSBs

To understand how SUMOylation regulates GNL3 function in HR repair, we examined the DNA damage-induced foci formation of key DDR proteins. We found that knockdown of GNL3 does not abolish γH2AX foci formation in cells treated with ETO (Supplementary Fig. S2b, c). Interestingly, DNA damage induced by depletion of endogenous GNL3 fails to induce RAD51 foci (Fig. 3k, l). Also, knockdown of GNL3 abolishes ETO treatment-induced RAD51 foci formation, which can be completely rescued by ectopic expression of WT GNL3, but not the K196R mutant in both U2OS cells (Fig. 3k, l) and HeLa cells (Supplementary Fig. S3g, h). Knockdown of GNL3 also abolishes RPA2 foci formation induced by ETO treatment (Fig. 3m, n). Intriguingly, knockdown of GNL3 still induced the BRCA1 foci formation (Supplementary Fig. S4a, b), consistent with the report by Meng et al^61^. The induced BRCA1 foci was abolished by ectopic expression of siRNA-resistant WT GNL3, but not the K196R mutant (Supplementary Fig. S4a, b). In addition, GNL3 knockdown also induced the foci formation of BARD1 (Supplementary Fig. S4c, d), MDC1 (Supplementary Fig. S4e, f), Mre11 (Supplementary Fig. S4g, h), CtIP (Supplementary Fig. S4i, j), EXO1 (Supplementary Fig. S4k, l), and 53BP1 (Fig. 3o, p). These results suggest that GNL3 may play a role in HR repair by regulating the RPA2 and RAD51 loading without interfering with the tested upstream DDR proteins acting prior to RAD51 loading, including BRCA1, BARD1, MDC1, CtIP and EXO1. Of note, SUMOylation does not affect the predominant nucleolar localization of GNL3 as the K196R mutant still mainly localized in the nucleolus similar to WT GNL3, as determined by IF staining, where the GTP-binding-defective G261V mutant localizes in the nucleoplasm (Supplementary Fig. S4m).

### GNL3 regulates DNA end resection by interacting with the BLM-DNA2 complex in response to DSBs, dependently on its SUMOylation

**Fig. 4:**
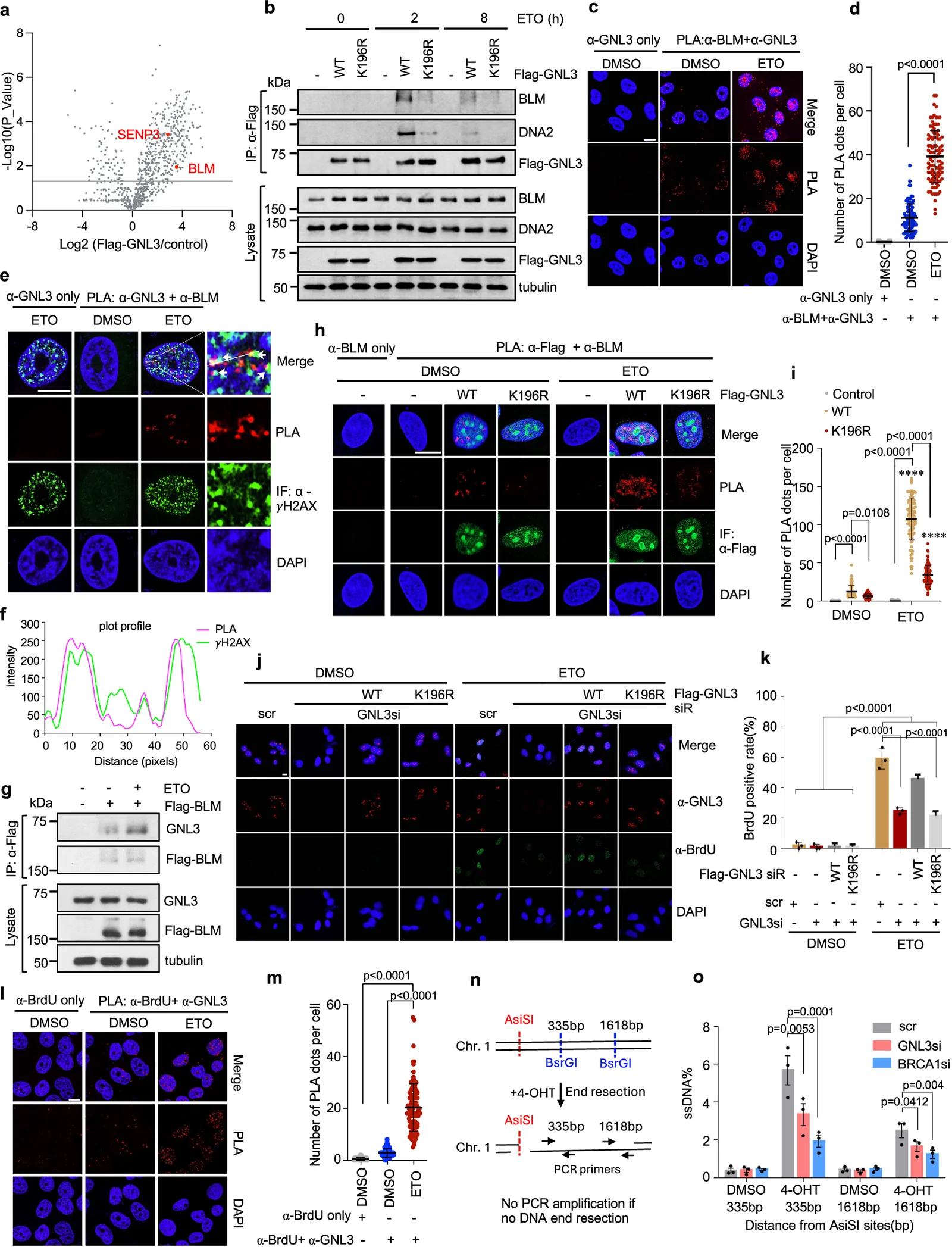
GNL3 regulates DNA end resection by interacting with the BLM-DNA2 complex. **a** Volcano plot of quantitative MS analysis of affinity-purified Flag-GNL3- interactome. X-axis represents the Log2 ratio of peptide counts from Flag-GNL3 immunoprecipitates to control. The significant cut line is set at *P* < 0.05. *n* = 3 biological replicates. **b** U2OS cells transfected with the indicated plasmids were treated with 20 μM ETO for the indicated times and assayed by co-IP. Quantification of immunoprecipitated BLM and DNA2 from three independent experiments is shown in Supplementary Fig. S5a. **c–f** U2OS cells treated with DMSO or ETO were assayed by PLA (**c**, **d**) and PLA followed by IF staining (**e**, **f**). Representative confocal images with a zoom-in region in the right (**e**) and an intensity plot of PLA signal and γH2AX foci of the zoom-in region (**f**) are shown. **g** U2OS cells transfected with Flag-BLM and treated with control or ETO were assayed by co-IP. *n* = 2 independent experiments. **h**, **i** U2OS cells transfected with the indicated plasmids were treated with DMSO or ETO and assayed by PLA. **j**, **k** IF of U2OS cells transfected with the indicated siRNA and plasmids were cultured in the presence of 10 μM BrdU. Item k depicts *n* = 147,110, 155, 144, 166, 226, 175, 193 cells per condition (from left to right) from three independent experiments. **l**, **m** U2OS cells cultured in the presence of 10 μM BrdU for 20 h were treated with DMSO or ETO and assayed by PLA. **n** Diagram showing an *Asi*SI cleavage site at chromosome 1, two BsrGI enzyme digestion sites, and two pairs of primers for PCR amplification. **o** ER-*Asi*SI U2OS cells transfected with indicated siRNA were treated with 4-OHT followed by qPCR detection of ssDNA (*n* = 3 biological replicates). Treatment with DMSO or 20 μM ETO for 2 h were used for all the experiment. Scale bars, 10 μm. Representative images (**c**, **e**, **h**, **j**, **l**) and n = 100 cells/condition (**d**, **i**, **m**) from three independent experiments are shown. Error bars denote mean ± SD (**d**, **i**, **k**, **m**) and mean ± SEM (**o**). Significance was determined by unpaired two-tailed *t*testing (**d**, **m**) and two-way ANOVA, Tukey’s multiple comparisons test (**i**, **k**, **o**). ****P < 0.0001, compared to the respective DMSO-treated group.

As GNL3 SUMOylation is required for RAD51 and RPA foci formation, GNL3 knockdown significantly increased 53BP1 foci formation upon ETO treatment (Fig. 3o, p), and our affinity purification and mass spectrometry analyses identified BLM in the Flag-GNL3-associated protein complexes (Fig. 4a and Supplementary Data 1), we next asked whether GNL3 SUMOylation may play a role in DNA end resection. We first tested whether GNL3 interacts with the BLM-DNA2 complex critical for long-range DNA end resection. Indeed, co-IP assays showed that treatment of cells with ETO markedly promoted Flag-GNL3 interaction with endogenous BLM and DNA2 (Fig. 4b and Supplementary Fig. S5a). PLA assays showed that GNL3 interacts with BLM endogenously upon ETO treatment (Fig. 4c, d). The GNL3-BLM PLA signals largely co-localize with γH2AX in ETO-treated cells (Fig. 4e, 4f), suggesting that GNL3 interacts with BLM at sites of DNA damage. Flag-BLM also co-immunoprecipitated with endogenous GNL3 and this interaction was significantly enhanced by ETO treatment (Fig. 4g). Interestingly, the ETO-induced interaction between GNL3 and the BLM-DNA2 complex was markedly attenuated by mutating the SUMOylation site lysine K196 (Figs. 4b, 4h, 4i & Supplementary Fig. S5a, b). We next examined whether GNL3 plays a role in DNA end resection. We performed 5-bromo-2’-deoxyuridine (BrdU) incorporation assays followed by IF staining under native conditions with anti-BrdU antibody, which recognizes ssDNA only in non-denaturing conditions^87^. As shown in Fig. 4j, k, knockdown of GNL3 markedly reduced the number of BrdU-positive cells upon ETO treatment, indicative of impaired DNA end resection and ssDNA generation. This defect was again rescued by WT GNL3, but not the K196R mutant. Thus, GNL3 SUMOylation plays a key role in regulating DNA end resection by interacting with the BLM-DNA2 complex.

To further consolidate this finding, we sought to test whether GNL3 itself associates with ssDNA. We developed a PLA-based assay, referred to as ssDNA-PLA^88^, to detect the interaction between GNL3 and BrdU-incorporated ssDNA. U2OS cells were first cultured in the presence of BrdU for 20 h and treated with ETO or control DMSO for 2 h. The cells were stained with anti-BrdU and anti-GNL3 antibodies, followed by PLA under non-denaturing conditions, in which the anti-BrdU specifically recognizes BrdU-incorporated ssDNA, but not double-stranded (ds) DNA. As shown in Fig. 4l, m, ETO treatment markedly increased the association between GNL3 and BrdU-incorporated ssDNA. Thus, GNL3 interacts with ssDNA upon induction of DSBs. To directly assess the role of GNL3 in DNA end resection, we performed DNA end resection assays using the ER-*Asi*SI U2OS cell system^85,86^. In this system, 4-OHT treatment- induced activation of *Asi*SI enzyme generates site-specific DSBs. Genomic DNAs are then digested with the restriction enzyme BsrG1, which cleaves dsDNA, but not ssDNA. Thus, qPCR can be used to quantify relative levels of ssDNA generated by DNA end resection (Fig. 4n), where increased PCR products reflect increased DNA resection. Using this approach, we showed that knockdown of GNL3 markedly reduced ssDNA levels at an *Asi*SI-induced DSB site on chromosome 1, similar to BRCA1 knockdown (Fig. 4o and Supplementary Fig. S5c). Together, these results demonstrate that GNL3 is critical for DNA end resection.

### SUMO-SIM interaction directs the binding between GNL3 and BLM

**Fig. 5:**
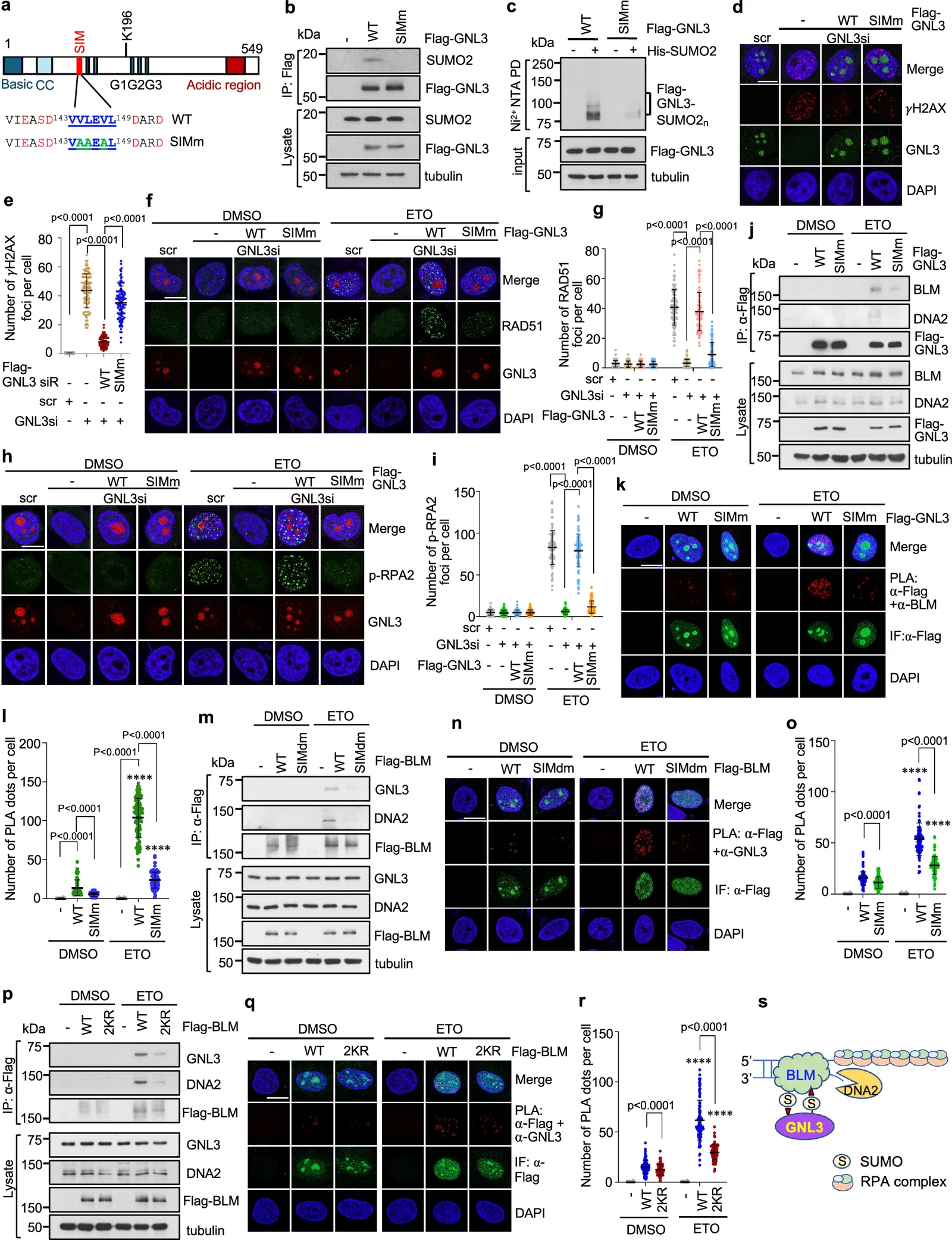
SUMO-SIM interaction directs the GNL3 interaction with BLM. **a** A diagram of GNL3 showing the sequences of the putative SIM sequence and the mutated SIM. Underlined are the SIMs flanked by acidic residues. **b** U2OS cells transfected with WT GNL3 or the SIM mutant were assayed by co-IP. *n* = 3 independent experiments. **c** H1299 cells transfected with the indicated plasmids were assayed for GNL3 SUMOylation by Ni^2+^-NTA PD. *n* = 3 independent experiments. **d**, **e** U2OS cells transfected with scr or GNL3 siRNA together with siRNA-resistant Flag-GNL3 (WT or SIMm) were assayed by IF. **f–i** U2OS cells transfected with scr or GNL3 siRNA together with siRNA-resistant Flag-GNL3 (WT or SIMm) were treated with DMSO or 20 μM ETO for 2 h and assayed for RAD51 (**f**, **g**) and phospho-RPA2 (**h**, **i**) foci formation. **j–l** U2OS cells transfected with Flag-GNL3 WT or the SIM mutant were treated with DMSO or 20 μM ETO for 2 h and assayed by co-IP (**j**) (*n* = 2 independent experiments) and PLA (**k**, **l**). **m–o** U2OS cells transfected with Flag-BLM WT or the mutant with mutations of both SIMs (SIMdm) were treated with control or 20 μM ETO for 2 h and assayed by co-IP (**m**) and PLA (**n**, **o**). **p–r** U2OS cells transfected with Flag-BLM WT or the K317R;K331R (2KR) mutant were treated with control or 20 μM ETO for 2 h and assayed by co-IP (**p**) and PLA (**q**, **r**). **s** Schematic diagram showing the interaction of GNL3 and BLM via mutual SUMO-SIM interactions. S denotes SUMO. Scale bars, 10 μm. Representative confocal images (**d**, **f**, **h**, **k**, **n**, **q**) and quantification (**e,** **g,** **i,** **l,** **o,** **r**) of n = 100 cells per condition from three independent experiments are shown. Error bars denote mean ± SD. Significance was determined by one-way ANOVA Tukey’s multiple comparisons test (**e**) and two-way ANOVA, Tukey’s multiple comparisons test (**g**, **i**, **l**, **o**, **r**). ****P < 0.0001, compared to the respective DMSO control.

To understand how GNL3 SUMOylation regulates its interaction with BLM, we reasoned that this interaction may involve SUMO- SUMO-interacting motif (SIM) interactions. SIMs are typically characterized by a short stretch of core hydrophobic amino acids (V/I)x(V/I)(V/I), flanked by acidic residues^89^. It has been shown that BLM SUMOylation at K317 and K331 is necessary for its interaction with RAD51 and for efficient HR repair^32,33^. BLM also contains two SIMs, ^217^QIDL^220^ and ^235^VICI^238^, which are required for its SUMO binding and SUMOylation^90^. Similarly, RAD51 can be SUMOylated at K57 and K70 by TOPORS^38^ and contains a SIM, ^261^VAVV^264^. Both RAD51 SUMOylation and its SIM are required for its function in HR repair^38,39^. Notably, RAD51 binds to SUMO and SUMOylated BLM^33^. Thus, BLM SUMOylation plays a critical role in DNA end resection and RAD51 loading by SUMO- and SIM-mediated BLM-RAD51 interactions. Since SUMOylation of GNL3 promotes its interaction with both BLM and RAD51, it is conceivable that GNL3 SUMOylation regulates its interaction with BLM and RAD51 via SUMO-SIM interactions. To test whether GNL3 also contains SIMs, we mutated a putative SIM predicted by in silico analysis, ^143^VVLEVL^149^ to ^143^VAAEAL^149^ (Fig. 5a). The resulting GNL3^SIMm^ mutant failed to interact with either endogenous SUMO2 (Fig. 5b and Supplementary Fig. S5d) or ectopically expressed non-conjugatable form of SUMO2, SUMO2G, which contains only one Gly residue at the C-terminus (Supplementary Fig. S5e). This mutation also abolished GNL3 SUMOylation (Fig. 5c), confirming that this sequence is an authentic SIM. Notably, the GNL3^SIMm^ mutant failed to abolish γH2AX foci formation upon DNA damage induced by knockdown of endogenous GNL3 (Fig. 5d, e). The GNL3^SIMm^ mutant also failed to rescue ETO-induced RAD51 (Fig. 5f, g) and phospho-RPA2 (Fig. 5h, i) foci formation in cells with knockdown of endogenous GNL3. Further, mutating the SIM significantly attenuated the GNL3 interaction with BLM and DNA2, as determined by co-IP (Fig. 5j and Supplementary Fig. S5f, g) and PLA assays (Fig. 5k, l and Supplementary Fig. S5h). This reduction is not due to decreased binding of GNL3 to the chromatin in general (Supplementary Fig. S5i). To examine whether GNL3 interacts with BLM at sites of DNA damage, we performed knockdown and rescue experiments in cells treated with ETO. WT GNL3, but not the GNL3^SIMm^ mutant, interacted with BLM and the corresponding PLA signals largely colocalized with γH2AX foci (Supplementary Fig. 6a–c). ssDNA-PLA assays further showed that the GNL3^SIMm^ mutant failed to rescue ETO-induced recruitment of RAD51 (Supplementary Fig. S6d, e) and phospho-RPA2 (Supplementary Fig. S6f, g) to ssDNA in cells depleted of endogenous GNL3, indicating a defect in DNA end resection upon ETO treatment. Collectively, these results suggest that the GNL3 SIM is critical for mediating interaction with BLM and for promoting HR via SUMO-SIM interactions.

To further test this possibility, we generated a BLM mutant, BLM^SIMdm^, in which both of its SIMs were mutated^90^. We found that mutating the two SIMs in BLM markedly reduced its interaction with GNL3 in response to ETO treatment, as determined by both co-IP (Fig. 5m) and PLA (Fig. 5n, o and Supplementary Fig. S7a) assays. We also generated the SUMOylation-defective K317R and K331R BLM mutant^32^, BLM^2KR^, and observed a marked attenuation of the binding of GNL3 to the BLM^2KR^ mutant compared to WT BLM upon ETO treatment, as shown by both co-IP (Fig. 5p) and PLA (Fig. 5q, r and Supplementary Fig. S7b) assays. Similar results were observed using GFP-tagged BLM^2KR^ mutants (Supplementary Fig. S7c). Together, these results suggest that the GNL3-BLM association is mediated by dual SUMO-SIM interactions (Fig. 5s). Of note, PLA assays further showed that knockdown of BLM significantly reduced the interaction of GNL3 with γH2AX (Supplementary Fig. S7d–f), suggesting that GNL3 recruitment to DSB sites depends on its interaction with BLM.

### USP36 is a SUMO ligase for GNL3

**Fig. 6:**
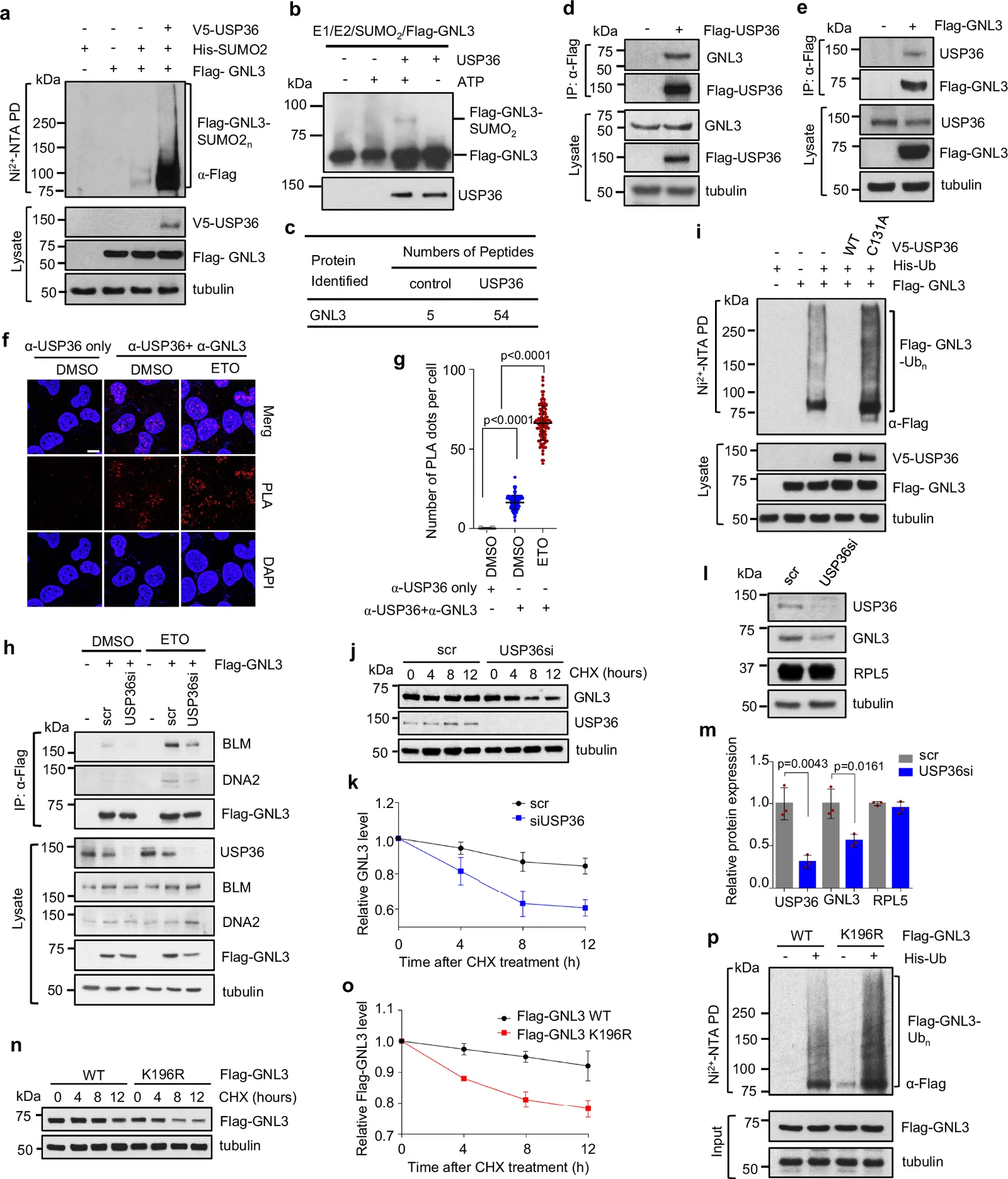
USP36 is a GNL3 SUMO ligase. **a** H1299 cells transfected with the indicated plasmids were assayed for GNL3 SUMOylation by Ni^2+^-NTA PD under denaturing conditions. **b** In vitro SUMOylation assays using recombinant SUMO E1, Ubc9 (E2), SUMO2 and purified Flag-GNL3 in the presence or absence of ATP and purified USP36. **c** GNL3 peptide identified in affinity-purified Flag-USP36 complexes compared to control. **d**, **e** 293 cells transfected with Flag-USP36 (**d**) or Flag-GNL3 (**e**) were assayed by co-IP using anti-Flag antibody. **f**, **g** U2OS cells treated with DMSO or 20 μM ETO for 2 hours were assayed by PLA using anti-USP36 and anti-GNL3 to detect the interaction of endogenous USP36 and GNL3. Scale bar, 10 μm. Representative images (**f**) and quantification (**g**) of *n* = 106, 106, 102 cells per condition (left to right) from three independent experiments are shown. **h** U2OS cells transfected with Flag-GNL3 together with scr or USP36 siRNA were treated with DMSO or 20 μM ETO for 2 h and assayed by co-IP. **i** H1299 cells transfected with the indicated plasmids were assayed by Ni^2+^-NTA-PD to detect the ubiquitinated GNL3. **j**, **k** U2OS cells transfected with scr or USP36 siRNA were treated with cycloheximide (CHX) for different times and assayed by IB (**j**). **l**, **m** U2OS cells transfected with scr or USP36 siRNA were assayed by IB. Representative results (**l**) and quantification (**m**) from three independent experiments are shown. **n**, **o** U2OS cells transfected with Flag-GNL3 (WT or K196R) were treated with CHX for different times and assayed by IB (**n**). Quantification is shown in (o) from three biological replicates. **p** H1299 cells transfected with the indicated plasmids were assayed for GNL3 ubiquitination by Ni^2+^-NTA PD. Representative IB results from three (**a**, **b**, **d**, **e**, **h**, **i**, **j**, **I**, **n**) and two (**p**) independent experiments are shown. Error bars denote mean ± SD. Significance was determined by one-way ANOVA Tukey’s multiple comparisons test (**g**) and two-tailed *t*testing (**m**), respectively.

To identify SUMO ligases that mediate GNL3 SUMOylation, we examined members of the PIAS family SUMO ligases and the nucleolar SUMO ligase USP36, a deubiquitinating enzyme (DUB) that also functions as a SUMO ligase, previously discovered by us^71–74^. We found that USP36 markedly promoted GNL3 SUMOylation, whereas PIAS1, but not other PIAS family members, only slightly promoted GNL3 SUMOylation (Fig. 6a and Supplementary Fig. S8a). In vitro SUMOylation assays using recombinant proteins showed that USP36 directly mediates GNL3 SUMOylation in the presence of E1 (SAE1/SAE2), E2 (Ubc9), SUMO2, and ATP (Fig. 6b). These data demonstrate that USP36 is a major SUMO ligase for GNL3. Consistently, GNL3 was identified in our previously purified USP36 complex^72^ (Fig. 6c). We confirmed the USP36-GNL3 interaction by co-IP (Fig. 6d, e). PLA further showed that the endogenous interaction between USP36 and GNL3 is significantly increased upon ETO treatment (Fig. 6f, g). RNase treatment did not disrupt the USP36-GNL3 interaction, whereas USP36 interaction with ribosomal protein L30 (RPL30) was largely abolished (Supplementary Fig. S8b), suggesting that USP36 interacts with GNL3 independently of RNA and intact ribosomes. Using co-IP assays, we further mapped that both the N-terminal USP domain and the C-terminal domain of USP36 interact with GNL3 (Supplementary Fig. S8c, d). IF staining (Supplementary Fig. S8e) and cell fractionation assays (Supplementary Fig. S8f) showed that both USP36 and GNL3 are predominantly localized in the nucleolus. Supporting its role in SUMOylating GNL3 and HR repair, knockdown of USP36 significantly reduced the interaction between GNL3 and the BLM-DNA2 complexes (Fig. 6h) and decreased HR, but not NHEJ, repair activity, as determined by DNA repair reporter assays (Supplementary Fig. S8g). These results identify USP36 as a novel regulator of GNL3-mediated DDR pathway and HR repair.

### USP36 deubiquitinates GNL3 and regulates its levels

Given that USP36 is a DUB, we next examined whether it deubiquitinates GNL3. We transfected cells with Flag-GNL3 along with either WT USP36 or its catalytically inactive mutant (C131A). In vivo ubiquitination assays under denaturing conditions showed that WT USP36, but not the C131A mutant, completely abolished GNL3 ubiquitination (Fig. 6i). Consistently, USP36 knockdown significantly decreased the stability of GNL3 as determined by protein half-life assays (Fig. 6j, k) and reduced the levels of GNL3, but not RPL5 (Fig. 6l, m). Thus, USP36 acts as a SUMO ligase/DUB dual-function enzyme for GNL3. Interestingly, the SUMOylation-defective K196R mutant showed reduced protein stability (Fig. 6n, o) and increased ubiquitination (Fig. 6p), suggesting that K196 SUMOylation inhibits GNL3 ubiquitination and proteasome-mediated degradation.

### SENP3 deSUMOylates GNL3 and is also required for DNA damage repair

**Fig. 7:**
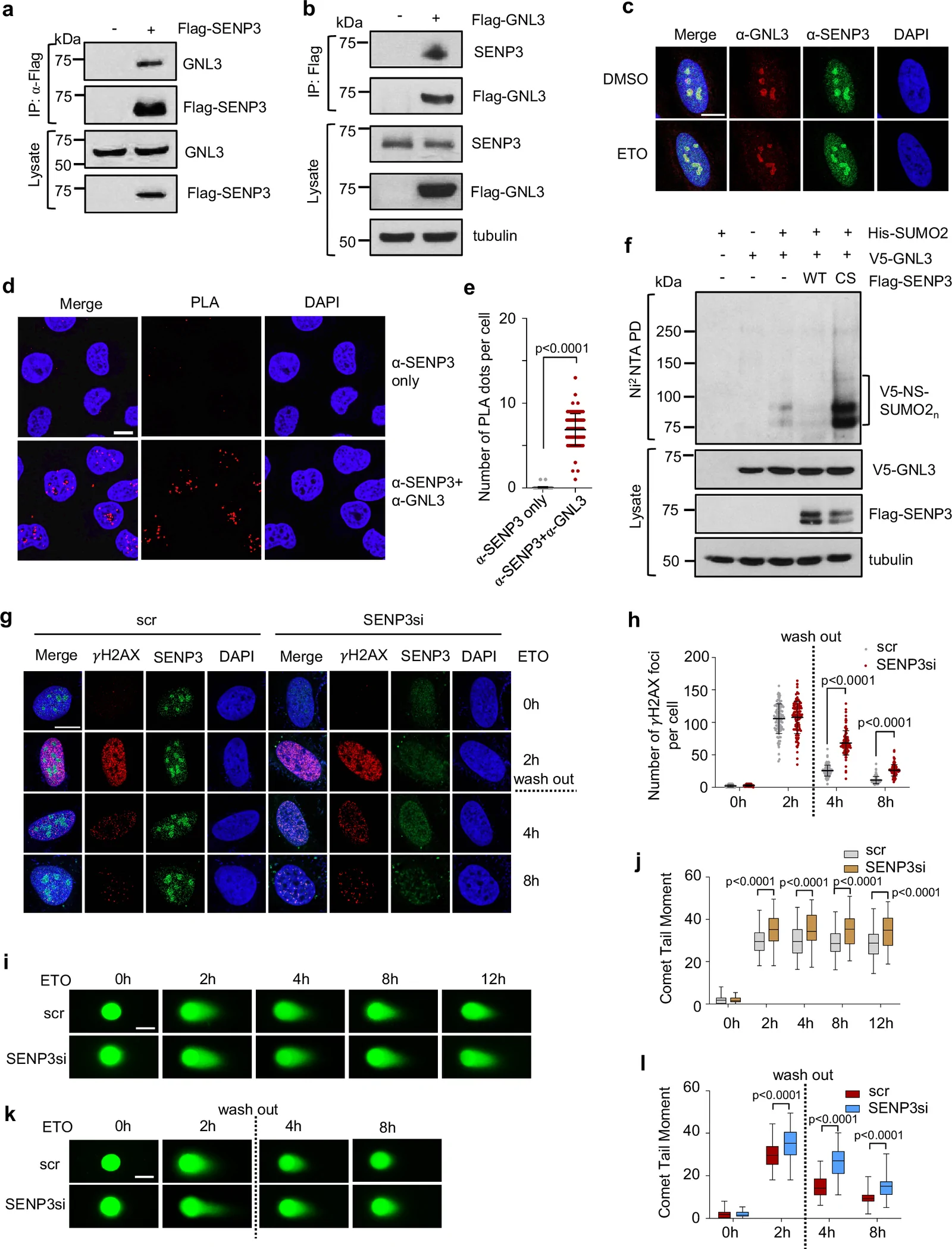
SENP3 interacts with and deSUMOylates GNL3. **a**, **b** 293 cells transfected with Flag-SENP3 (**a**) or Flag-GNL3 (**b**) were assayed by co-IP. **c** U2OS cells were treated with DMSO or 20 μM ETO for 2 hours and assayed by IF staining. **d**, **e** Endogenous SENP3 interacts with GNL3 determined by PLA assays. **f** H1299 cells transfected with the indicated plasmids were assayed for GNL3 SUMOylation by Ni^2+^-NTA PD. **g**, **h** U2OS cells transfected with scr or SENP3 siRNA were treated with or without 20 μM ETO for 2 hours followed by washing out and replacement with fresh medium. The cells were fixed at the indicated times and assayed by IF using anti-γH2AX and anti-SENP3. **i–l** Comet assays were conducted in U2OS cells transfected with scr or SENP3 siRNA and treated without or with 20 μM ETO for different times indicated (**i**, **j**) or the medium was washed out and replaced with fresh medium and harvested at the indicated time (**k**, **l**). **a**, **b**, **c**, **f**, *n* = 3 independent experiments. Scale bars, 10 μm. Representative images (**d**, **g**, **I**, **k**) and quantification (**e**, **h**, **j**, **l**) of *n* = 100 cells per condition from three independent experiments are shown. **h**, **j**, **l**, the centerline of the boxplots represents the median value (50th percentile), the box encapsulates the interquartile range, and the whiskers extend to show the rest of the distribution. Significance was determined by unpaired two-tailed *t*testing (**e**) and two-way ANOVA, Tukey’s multiple comparisons test (**h**, **j**, **l**), respectively.

DeSUMOylation of DDR proteins by SUMO proteases such as SENP3 and SENP6 has been shown to regulate DSB repair^30,34^. Given that both SENP3 and GNL3 are predominantly localized to the nucleolus and shuttle between the nucleolus and the nucleoplasm^51,91–93^, and that SENP3 was also present in our purified Flag-GNL3-associated protein complexes (Fig. 4a), we examined whether SENP3 regulates GNL3. We first confirmed the SENP3-GNL3 interaction by co-IP (Fig. 7a, b). This interaction is RNA-independent (Supplementary Fig. S9a, b) and the N-terminal domain of SENP3 is sufficient for binding to GNL3 (Supplementary Fig. S9c, d). Notably, SENP3 and GNL3 co-localize mainly in the nucleolus, and this localization is not affected by ETO treatment (Fig. 7c). PLA assays further confirmed the endogenous interaction between GNL3 and SENP3 (Fig. 7d, e). Next, we examined whether SENP3 deSUMOylates GNL3. As shown in Fig. 7f, WT SENP3, but not its catalytically inactive C532S mutant, markedly reduced GNL3 SUMOylation, indicating that SENP3 acts as a SUMO protease for GNL3. SENP3 knockdown did not affect the nucleolar localization of either WT GNL3 or the K196R mutant (Supplementary Fig. S9e). As SUMOylation increases GNL3 stability (Fig. 6n–p), we examined whether SENP3-mediated deSUMOylation affects GNL3 levels. However, knockdown of SENP3 did not significantly affect the total levels of GNL3 (Supplementary Fig. S9f, g), suggesting that SENP3 does not impact the overall GNL3 abundance, although we cannot exclude the possibility that a small chromatin-associated pool of GNL3 may be subjected to SUMOylation-regulated turnover.

We next sought to determine whether SENP3 regulates DNA damage repair. As shown in Fig. 7g, h, knockdown of SENP3 markedly attenuated the clearance of γH2AX foci upon ETO treatment, indicative of an impaired DNA damage repair. Comet assays further showed that SENP3 knockdown resulted in increased tail moments compared to the scrambled control, both during ETO treatment and after drug washout (Fig. 7i, l and Supplementary Fig. 9h, i), suggesting a reduction of DNA damage repair efficiency. Consistently, DNA repair reporter assays demonstrate that SENP3 knockdown decreased both HR and NHEJ repair activities (Supplementary Fig. 9j). Together, these results indicate that SENP3 is essential for efficient DNA damage repair, consistent with the recently reported function of SENP3 in DNA damage repair by deSUMOylating TIP60, Mre11 and NPM^30,94–96^.

### GNL3 implication in breast cancer

**Fig. 8:**
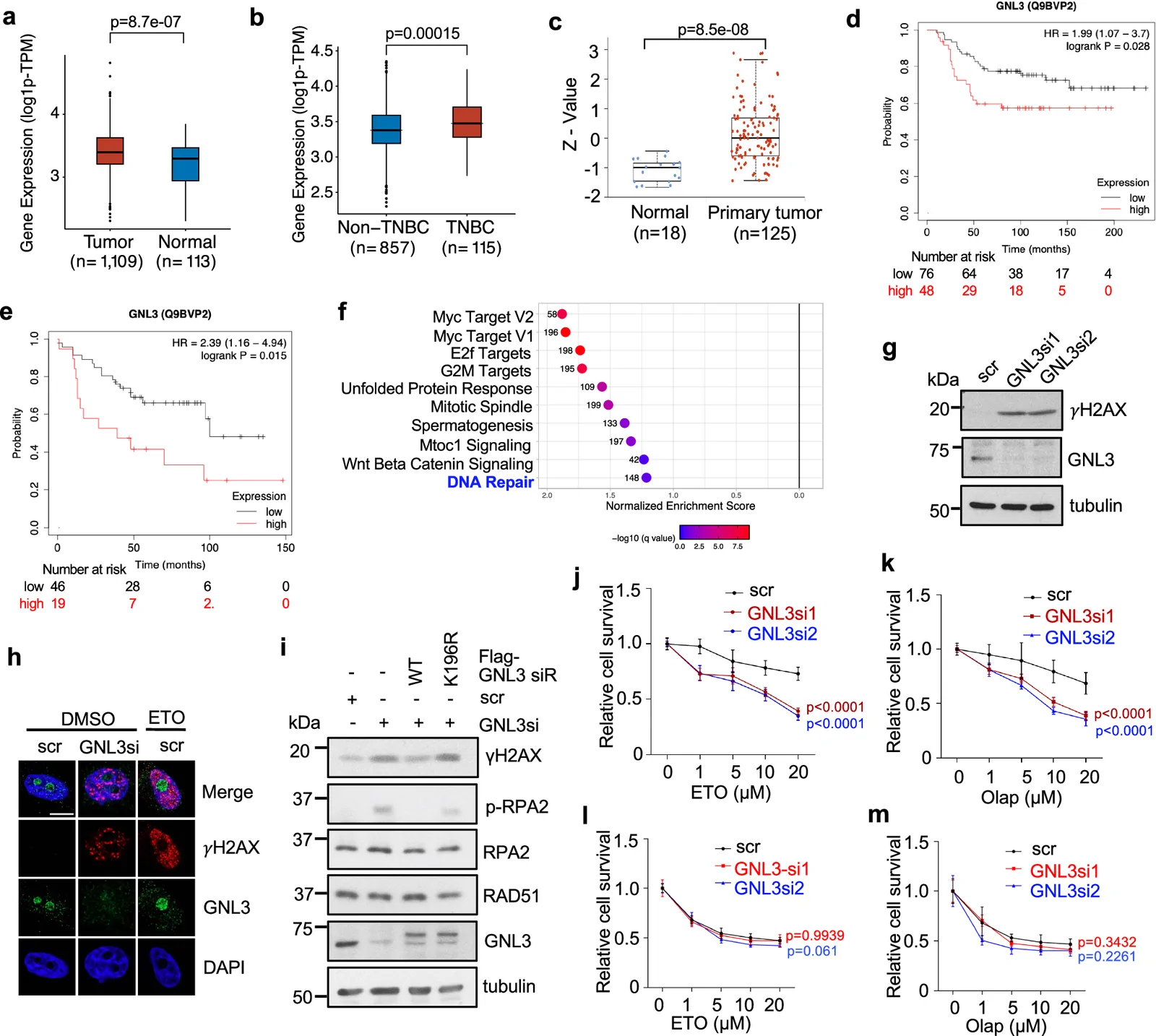
GNL3 is implicated in breast cancer. **a**, **b** GNL3 mRNA expression is significantly increased in BC compared to normal controls (**a**) and increased in TNBC compared to non-TNBC (**b**) (data from TCGA). Significance was determined by a two-sided Wilcoxon rank-sum test. **c** GNL3 protein expression is increased in BC compared to normal controls (data from CPTAC database). Z-values represent standard deviations from the median across samples. Significance was determined by unpaired two sample t-test. Box plots in (**a–c**), the centerline of the boxplots represents the median value (50th percentile), the box encapsulates the interquartile range, and the whiskers extend to show the rest of the distribution, except for outliers defined as 1.5 times the interquartile range. **d**, **e** High expression of GNL3 mRNA (**d**) and protein (**e**) is associated with poor patient survival (data from Kaplan-Meier plotter). **f** GNL3 expression is associated with the DNA damage hallmark in TNBC. Shown are the top ten hallmarks from TCGA data. **g**, **h** MDA-MB-231 cells were transfected with scr or GNL3 siRNA or treated with 20 μM ETO for 2 h and assayed by IB (**g**) and IF staining with anti-γH2AX for detecting γH2AX foci (**h**). *n* = 3 independent experiments. **i** MDA-MB-231 cells transduced with control, siRNA-resistant Flag-GNL3 (WT or K196R) retroviruses were then transfected with scr or GNL3 siRNAs and assayed by IB. *n* = 2 independent experiments. **j–m** MDA-MB-231 (**j**, **k**) and MDA-MB-436 (**l**, **m**) cells transfected with scr or GNL3 siRNAs were treated with different doses of ETO (**j**, **l**) or Olaparib (**k**, **m**), followed by MTT assays. *n* = 3 biological replicates. Error bars denote mean ± SD. Significance was determined by two-way ANOVA, Tukey’s multiple comparison test.

To examine whether the role of GNL3 and its SUMOylation in HR repair is implicated in tumorigenesis and therapeutic response, we focused on breast cancer, as TCGA data showed that GNL3 mRNA expression is significantly higher in primary breast cancers compared to normal control (Fig. 8a) and is significantly higher in triple-negative breast cancers (TNBCs) compared to non-TNBCs (Fig. 8b). Data from NCI Clinical Proteomic Tumor Analysis Consortium (CPTAC) database showed that GNL3 protein levels are significantly increased in breast cancers compared to normal control (Fig. 8c). High GNL3 protein expression is associated with poor patient survival in two available public datasets (Fig. 8d, e). Consistent with its role in HR repair, high expression of GNL3 is significantly associated with the DNA repair hallmark (Fig. 8f and Supplementary Fig. 10a). Collectively, these data suggest that GNL3 is overexpressed in breast cancers and that its role in HR could contribute to therapeutic resistance. We therefore next examined whether GNL3 SUMOylation plays a role in breast cancer therapeutic response. Consistent with the critical role for GNL3 and its SUMOylation in HR repair, knockdown of GNL3 also induced the γH2AX levels (Fig. 8g) and foci formation (Fig. 8h) in TNBC MDA-MB-231 cells. The induction of γH2AX and phospho-RPA2 upon GNL3 depletion was rescued by expression of WT GNL3, but not the K196R mutant (Fig. 8i and Supplementary Fig. S10b). Consistently, knockdown of GNL3 significantly increased the sensitivity of HR competent MDA-MB-231 cells (Fig. 8j, k), but not the HR deficient MDA-MB-436 cells (Fig. 8l, m and Supplementary Fig. 10c), to ETO and Olaparib, suggesting that GNL3 may represent a potential therapeutic target in HR-proficient cancers.

**Fig. 9:**
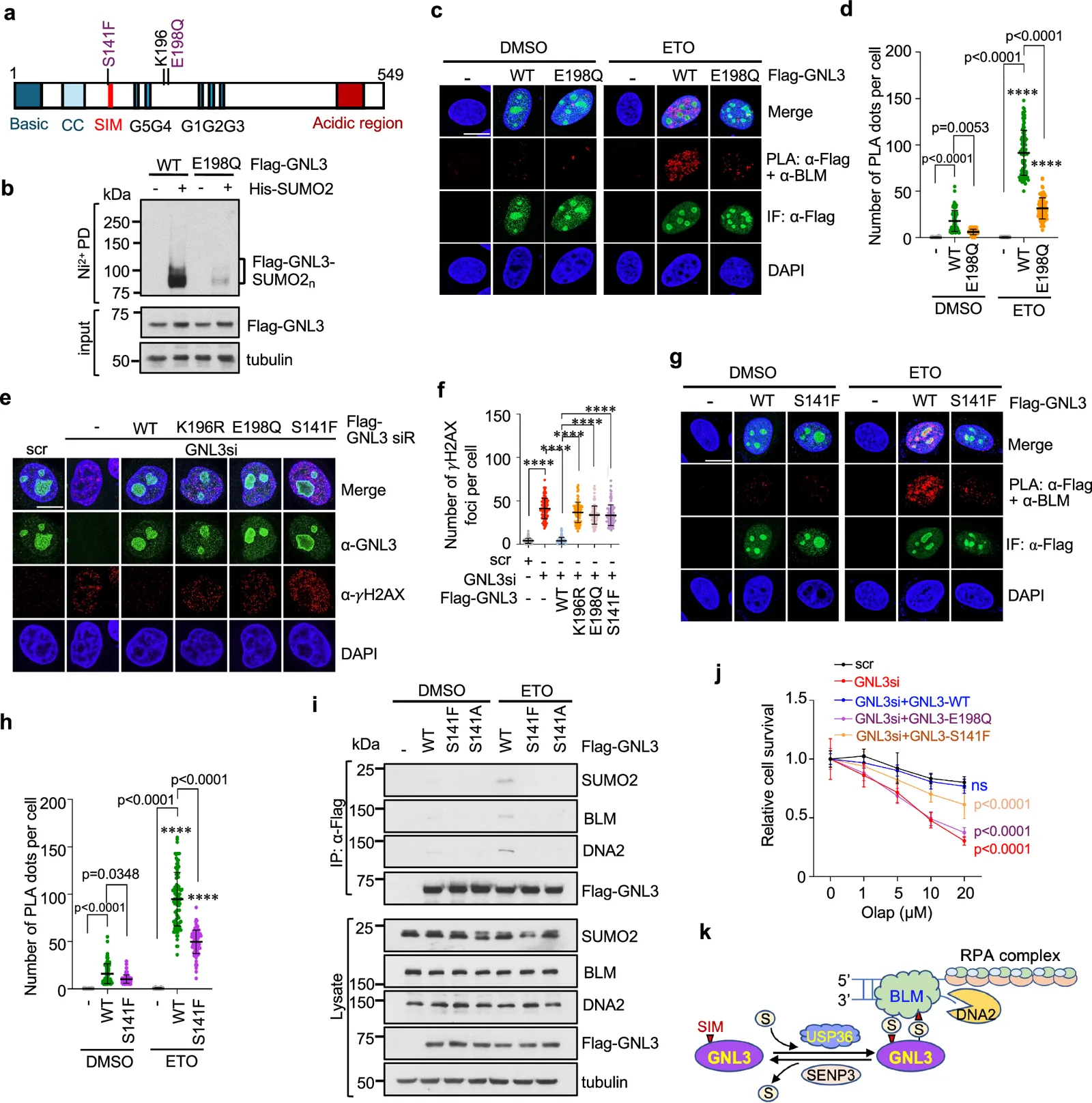
Breast cancer associated GNL3 varients disrupting its SUMOylation or SIM impair BLM interaction and DNA damage repair. **a** Diagram of GNL3 showing the breast cancer-derived mutations E198Q and S141F. **b** H1299 cells transfected with the indicated plasmids were assayed for GNL3 SUMOylation by Ni^2+^-NTA PD. *n* = 3 independent experiments. **c**, **d** U2OS cells transfected with Flag-GNL3 (WT or E198Q) were treated with DMSO or 20 μM ETO for 2 h and assayed by PLA. **e**, **f** HeLa cells transfected with scr or GNL3 siRNA together with siRNA-resistant Flag-GNL3 (WT, K196R, E198Q or S141F) were assayed by IF staining. ****P < 0.001, determined by one-way ANOVA Tukey’s multiple comparison test. **g**, **h** U2OS cells transfected with Flag-GNL3 (WT or S141F) were treated with control or 20 μM ETO for 2 h and assayed by PLA, followed by IF. **i** U2OS cells transfected with Flag-GNL3 (WT, S141F or S141A) were treated with control or 20 μM ETO for 2 h and assayed by co-IP. *n* = 2 independent experiments. **j** MDA-MB-231 cells were transduced with control, siRNA-resistant WT Flag-GNL3, E198Q or S141F mutant retroviruses and then transfected with scr or GNL3 siRNAs. Cells were treated with different doses of Olaparib for 24 h and assayed by MTT viability assay. *n* = 3 biological replicates. **k** Schematic diagram of the working model showing that USP36 and SENP3 regulate GNL3 interaction with BLM via their SUMO-SIM interactions. S denotes SUMO, and SIM denotes a SUMO-interacting motif. Scale bars, 10 μm. Shown are representative confocal images (**c**, **e**, **g**) and the quantification (**d**, **f**, **h**) of *n* = 100 cells per condition from three independent experiments are shown. Error bars denote mean ± SD. Significance was determined by two-way ANOVA, Tukey’s multiple comparison test (**d**, **h**, **j**). ns, not significant. ****P < 0.001, compared to the respective DMSO group (**d**, **h**).

Interestingly, several GNL3 variants of unknown significance (VUS) in the SUMO and SIM motifs are present in TCGA and MSK breast cancer patients, including E198Q (which disrupts the SUMO consensus site) and S141F (which reduces negative charge and likely weakens the SIM) (Fig. 9a). Mutating E198 to Gln (Q) abolished GNL3 SUMOylation (Fig. 9b and Supplementary Fig. S10d) and attenuated the interaction between GNL3 and BLM upon ETO treatment, as determined by PLA (Fig. 9c, d and Supplementary Fig. S10e). The E198Q mutant also failed to rescue GNL3 knockdown-induced DNA damage, as indicated by increased γH2AX levels (Supplementary Fig. S10f) and foci formation (Fig. 9e, f and Supplementary Fig. 10g, h), suggesting that the E198Q mutation disrupts the GNL3 SUMOylation pathway and impairs its function in HR repair. Additionally, mutating S141 also markedly reduced GNL3 binding to BLM and SUMO2 (Fig. 9g–i and Supplementary Fig. S10i). The S141F mutant also fails to rescue GNL3 knockdown-induced γH2AX levels (Supplementary Fig. S10f) and foci formation (Fig. 9e, f), suggesting that mutating S141 impairs the SIM function and compromises GNL3 function in HR. Notably, WT GNL3, but not the E198Q and S141F mutants, significantly reversed the sensitization of MDA-MB-231 cells to Olaparib induced by knockdown of endogenous GNL3 (Fig. 9j). Together, our results suggest that these VUSs attenuate the SUMO-dependent regulation of GNL3 and HR repair and may contribute to breast tumorigenesis.

## Discussion

Long-range DNA end resection by the BLM-DNA2 helicase-nuclease complex is crucial for DSB HR repair. However, how this critical enzymatic process is regulated in cells remains incompletely understood. In this study, we identified GNL3 as a key regulator of HR repair through its interaction with the BLM-DNA2 complex and its regulation of DNA end resection. Mechanistically, this process is tightly controlled by SUMOylation. We found that GNL3 is SUMOylated at K196 in response to DSBs and it contains a functional SIM. Both GNL3 SUMOylation and its SIM are required for its interaction with BLM and function in HR. Notably, BLM itself contains two SIMs and is regulated by SUMOylation^32,33,90^. We showed that either disrupting BLM SUMOylation or mutating the two SIMs impairs its interaction with GNL3. Together, these data demonstrate that the GNL3-BLM interaction is mediated by reciprocal SUMO-SIM interactions (Fig. 9k). In response to DSBs, GNL3 SUMOylation is markedly increased, promoting its interaction with the BLM-DNA2 complex. Thus, GNL3 acts as a critical regulator of the HR pathway by fine-tuning BLM-DNA2-mediated DNA end resection. Importantly, both the K196R and SIM-deficient GNL3 mutants fail to rescue DDR defects induced by endogenous GNL3 knockdown, suggesting that GNL3 SUMOylation is also essential for protecting cells from replication-associated spontaneous DNA damage.

Supporting its role in regulating DNA end resection, GNL3 localizes to sites of DNA damage, as indicated by its association with γH2AX and ssDNA. Our DI-PLA (Fig. 3c, d) and ChIP (Fig. 3f) assays further demonstrated that GNL3 is enriched at DSB sites induced by ETO treatment or by *Asi*SI-mediated site-specific cleavage. The GNL3-ssDNA intermediates may contribute to the regulation of BLM-DNA2 recruitment upon DSBs by stabilizing the short-range ssDNA generated by the MRN-CtIP. Through its interaction with BLM, GNL3 may also directly stimulate BLM helicase activity. Indeed, both RPA^97^ and CtIP^98^ have been shown to interact with BLM and enhance its helicase activity. Chromatin-associated factors such as recently reported ZNF280A have been shown to promote long-range DNA end resection by facilitating recruitment of the BLM-DNA2 complex to DSB sites and enhancing its activity^99^. The BLM complex includes additional components, such as BMI1, BMI2, and TOP3A^100^. Interestingly, both BMI1 and TOP3A are regulated by SUMOylation^101–104^. Thus, it will be interesting to examine whether SUMOylation regulates GNL3 interaction with other components of the BLM complex, including BMI1, BMI2, and TOP3A, and whether DNA damage triggers the coordinated BLM complex-GNL3 protein group SUMOylation. In addition, BLM plays a critical role in RAD51 loading through SUMO- and SIM-mediated interactions with RAD51^31^ and promotes synapsis during HR by stabilizing RAD51 in the displacement loop (D-loop) upon template strand invasion^105^. We showed that both the GNL3-RAD51 interaction and RAD51 foci formation following DSB induction depend on GNL3 SUMOylation, suggesting that GNL3-RAD51 interaction may directly contribute to RAD51 loading and stabilization within the D-loop. Future studies are warranted to elucidate the precise mechanism by which the GNL3-BLM axis contributes to DNA end resection and HR repair.

Intriguingly, we found that several breast cancer-derived VUSs, including E198Q and S141F, impair the SUMOylation-regulated GNL3-BLM interaction. This suggests that these variants could contribute to mammary tumorigenesis by compromising HR repair and promoting genomic instability, thus being pathogenic mutations in breast cancer. This finding further reinforces the critical role of SUMO-dependent regulation of GNL3 in HR repair. Notably, depletion of GNL3 sensitizes HR-proficient MDA-MB-231 cells to Olaparib treatment, and this effect was reversed by expression of WT GNL3, but not the E198Q and S141F mutants (Figs. 8j, m, 9j). These findings suggest that GNL3 and its SUMOylation could be promising therapeutic targets in HR proficient and GNL3-overexpressing cancers. As GNL3 is an essential gene required for animal development and stem cell maintenance^51,60,106^, targeting its SUMOylation may represent a promising strategy to sensitize cancer cells to DNA-damaging agents while minimizing effects on normal cellular functions. In this context, it is imperative to determine whether GNL3 SUMOylation is also essential for its roles in cell growth and cell cycle regulation, particularly in ribosome biogenesis and cell cycle control.

GNL3 is a nucleolar-nucleoplasm shuttling protein that predominantly localizes to the nucleolus. DNA damage or abolishing GNL3 SUMOylation does not appear to alter its predominant nucleolar localization. GNL3 has been shown to interact with γH2AX in the nucleoplasm of S-phase cells following HU treatment^61^. Consistently, we showed that GNL3 co-localizes with γH2AX in the nucleoplasm following ETO treatment (Supplementary Fig. S3c, d). PLA assays further revealed that the GNL3-BLM interactions occur throughout the nucleus and co-localize with γH2AX foci (Fig. 4e, f). Importantly, it has been shown that the nucleolar localization of GNL3 is not required for its genome-protective function^61^. Together, these observations suggest that a subset of SUMOylated GNL3 in the nucleoplasm may be critical for HR. Nucleolar integrity is known to be impaired by most, if not all, cellular stressors, including DNA damage^107^. Thus, subtle changes in nucleolar dynamics may facilitate the re-localization or exchange of GNL3, enabling its interaction with BLM and regulation of DSB repair via HR, particularly during the S and G2 cell cycle phases when HR is active. Yet, this does not exclude the possibility that the nucleolar localization of GNL3 may also contribute to HR regulation, given that many DDR proteins transiently localize to the nucleolus^108^. Particularly, we found that GNL3 SUMOylation is tightly regulated by the nucleolar SUMO ligase USP36 and SUMO protease SENP3 (see below).

USP36 SUMOylates several nucleolar ribosome biogenesis factors, including the snoRNP complex components, EXOSC10 and Las1L, and plays a critical role in pre-rRNA processing and translation^71,72,74^. We showed that USP36 also acts as a SUMO ligase for GNL3. Knockdown of USP36 significantly reduced GNL3-BLM interaction upon DSBs, suggesting that USP36 contributes to the DDR, at least in part by regulating GNL3. In addition, we showed that USP36 can interact with GNL3 in the nucleolus. Thus, USP36 may SUMOylate GNL3 in the nucleolus, thereby promoting its interaction with BLM complex and facilitating its shuttling to nuclear chromatin to execute HR function upon DNA damage. Interestingly, USP36 also deubiquitinates GNL3 and regulates its protein levels, indicating a functional crosstalk between SUMOylation and ubiquitination in regulating GNL3 levels. Thus, USP36 acts as a dual-function enzyme, functioning as both a DUB and a SUMO ligase for GNL3 to regulate its SUMOylation and protein levels in response to DNA damage. GNL3 has recently been shown to play a role in overcoming DNA replication stress by sequestering the DNA replication initiation factor ORC2 in the nucleolus, thereby controlling origin firing and indirectly preventing nascent DNA resection by MRN-CtIP nucleases^68^. Given our findings that GNL3 SUMOylation regulates the BLM-DNA2 pathway, these results suggest an additional mechanism by which GNL3 may directly contribute to DNA damage repair in response to replication stress.

The role of SENP3 in DSB repair has been studied by several groups, particularly its function in deSUMOylating key DDR proteins including Mre11^30^, NPM^96^ and TIP60^94^. We show that SENP3 also deSUMOylates GNL3, and that SENP3 depletion impairs DSB repair, as evidenced by the delayed γH2AX clearance. These findings suggest that SENP3-mediated deSUMOylation is important for efficient HR, potentially by facilitating the removal of the SUMOylated DDR proteins, including GNL3, from the chromatin following repair. Since knockdown of SENP3 does not significantly affect total GNL3 protein levels, it remains to be determined whether SENP3 regulates the turnover of chromatin-associated GNL3 following DNA damage via the ubiquitination-proteasome system. Future work will focus on understanding how USP36 and SENP3 coordinately regulate GNL3 SUMOylation in response to DNA damage, and whether their activities are themselves regulated by post-translational modifications such as ATM/ATR-mediated phosphorylation following DSB induction.

In summary, we discovered that GNL3 SUMOylation is essential for protecting cells from spontaneous DNA damage as well as DSBs induced by exogenous agents. GNL3 has been shown to prevent telomere DNA damage by recruiting PML to SUMOylated TRF1^69^, raising the question of whether GNL3 SUMOylation is also required for this function. Together, our findings establish GNL3 as a novel regulator of DNA repair that safeguards genomic integrity through a SUMOylation-regulated mechanism.

## Methods

### Cell culture and reagents

Human U2OS, HEK293, HeLa, H1299, MDA-MB-231, and MDA-MB-436 cells were cultured in Dulbecco’s modified Eagle’s medium (DMEM) supplemented with 10% (vol/vol) fetal bovine serum (FBS), 50 U/mL penicillin, and 0.1 mg/mL streptomycin at 37 °C in a 5% CO_2_ humidified atmosphere. These cell lines were obtained from ATCC and routinely monitored for mycoplasma contamination. Manufacturers performed authentication through short tandem repeat profiling. ER-*Asi*SI U2OS cells^85,86^ were kindly provided by Dr. Gaëlle Legube (University of Toulouse, France) and cultured in DMEM supplemented with 10% charcoal-stripped FBS (Gibco) and 1 μg/ml puromycin (Invitrogen). Etoposide (ETO), camptothecin (CPT), hydroxyurea (HU), cycloheximide (CHX), doxycycline (Dox), 4-hydroxytamoxifen (4-OHT), N-ethylmaleimide (NEM) and iodoacetamide (IAA) were purchased from Sigma-Aldrich. ML792 was purchased from Selleckchem. RNase A and RNase T1 were purchased from Thermo Scientific.

### Plasmids, DNA transfections and site-directed mutagenesis

Flag-tagged GNL3 (Flag-GNL3) and Flag-BLM were cloned into the pcDNA3-2Flag vector by PCR. The GNL3 cDNA was also subcloned into pcDNA3-V5 and pcDNA4-TO vectors to generate V5-tagged GNL3 and Tet-inducible pcDNA4-TO-2Flag-GNL3 plasmids, respectively. Flag-GNL3 siRNA-resistant (Flag-GNL3-siR) plasmid was generated by site-directed mutagenesis using the QuikChange Site-Directed Mutagenesis Kit (Agilent Technologies), in which the GNL3 siRNA targeting sequence (GNL3 si-1) 5’-AAGAACTAAAACAGCAGCAGA-3’ was mutated to 5’-AGGAGTTGAAGCAACAACAAA-3’. The Flag-tagged GNL3 K196R and K275R mutants were generated by site-directed mutagenesis using Flag-GNL3 as a template. The Flag-tagged siRNA-resistant GNL3 K196R, E198Q, S141F, S141A, and SIM mutants were generated using the pcDNA4-TO-2Flag-GNL3-siR plasmid as a template. WT and siRNA-resistant GNL3 cDNAs were also cloned into the pcDNA4-TO vector to generate untagged GNL3 plasmids. The retroviral vector expressing Flag-GNL3 (pLPCX-Flag-GNL3) was constructed by PCR amplification from pcDNA3-2Flag-GNL3-siR at Hind III/Not I sites. The pLPCX-Flag-GNL3 E198Q and S141F mutants were then constructed by site-directed mutagenesis. Flag-BLM with K317R; K331R mutant (Flag-BLM^2KR^) and with mutation of the two SIMs (BLM^SIMdm^) were also generated by site-directed mutagenesis. Flag-SENP3 and its deletion mutants were generated by PCR cloning. V5-tagged SUMO2G plasmid with deletion of the last Gly residue was cloned by PCR. All the plasmids were confirmed by sequencing and are available upon request. See Supplementary Data 2 for all other plasmids and Supplementary Data 3 for sequences of primers used for plasmid construction. Cell transfection was performed using TransIT®-LT1 reagents (Mirus Bio Corporation) or Lipofectamine 2000 (Life Technologies) reagents following the manufacturer’s protocol. Cells were harvested at 36–48 h posttransfection for further analyses.

### Establishment of stable His10-SUMO2 and Flag-GNL3 expression cell lines

U2OS cells stably expressing His_10_-SUMO2 were generated by transient transfection with the pcDNA3-His_10_-SUMO2 plasmid, followed by selection in medium containing 0.5 mg/ml neomycin (G418) for up to 2 weeks. Single colonies were isolated, expanded, and screened by immunoblotting with anti-His antibodies. HEK293 cells stably expressing control or Flag-GNL3 were established by transfecting with control pcDNA3-Flag and Flag-GNL3 plasmids, respectively, followed by selection in medium containing 0.5 mg/ml G418. Single colonies were also isolated, expanded, and screened by immunoblotting with anti-Flag antibodies. All cells were maintained in culture medium containing 0.5 mg/ml G418.

### Immunoblot (IB) and co-immunoprecipitation (co-IP), and antibodies

Cells were lysed in NP40 lysis buffer consisting of 50 mM Tris-HCl (pH 8.0), 0.5% Nonidet P-40, 1 mM EDTA, 150 mM NaCl, 1 mM phenylmethylsulfonyl fluoride (PMSF), 1 mM dithiothreitol (DTT), 1 μg/ml pepstatin A, and 1 mM leupeptin with brief sonication. Equal amounts of total protein were used for IB analysis. For Co-IP, cells were lysed in the above NP40 lysis buffer in the presence of 20 mM NEM and 5 mM IAA. Equal amounts of cell lysates were used for co-IP using anti-Flag (M2) agarose beads at 4 °C for 4 h. Beads were washed four times with lysis buffer, and bound proteins were detected by IB using antibodies as indicated in the figure legends. A full list of antibodies and protocols can be found in Supplementary Data 4.

### Cell fractionation

Cell fractionation assays were performed essentially as described^73^. Briefly, the freshly harvested cells were washed with PBS, resuspended in hypotonic buffer A (10 mM HEPES pH 7.8, 10 mM KCl, 1.5 mM MgCl_2_, 0.5 mM DTT) in the presence of PMSF and incubated for 10 min on ice. The cells were homogenized using a douncer homogenizer B pestle followed by centrifugation at 228 x g for 5 min at 4 °C. The supernatant was supplemented with one-tenth volume of buffer B (0.3 M Tris-HCl pH 7.8, 1.4 M KCl, 30 mM MgCl_2_) and collected as the cytoplasmic fraction. The nuclear pellets were washed with buffer A once and then resuspended in buffer S1 (0.25 M sucrose, 10 mM MgCl_2_), layered over buffer S2 (0.35 M sucrose, 0.5 mM MgCl_2_), and centrifuged at 1430 x g for 10 min at 4 °C. The pelleted nuclei were resuspended in buffer S2 with PMSF and sonicated using a microtip probe at a power setting of 50%. The sonicated nuclei were layered over buffer S3 containing 0.88 M sucrose and 0.5 mM MgCl_2_, and centrifuged at 3,000 x g for 10 min at 4 °C. The supernatant was collected as the nucleoplasm fraction. The pellet contained purified nucleoli and was dissolved in high-salt RIPA buffer containing 50 mM Tris pH 7.5, 500 mM NaCl, 1% Nonidet P-40, 0.5% deoxycholate, and protease inhibitors in the presence of 80 U/mL DNase I on ice for 30 min. The lysates were then mixed with 2x volume of RIPA buffer without salt, incubated on ice for an additional 10 min, followed by centrifugation at maximum speed for 15 min. The supernatant was collected as soluble nucleolar fraction for IP analysis.

### Chromatin Fractionation

Soluble and chromatin fractions were extracted as previously described^30^. Cells were lysed in 400 μL EBC buffer A consisting of 50 mM Tris-HCl, pH 7.5, 100 mM NaCl, 1 mM EDTA, 2 μg/mL aprotinin, 20 mM NEM, 1 mM PMSF, 1 mM DTT, 0.05% NP40 for 8 min, followed by centrifugation at 800 x g for 5 min. The supernatants were collected as the soluble fractions. The pellets were then washed with EBC buffer A twice and a final wash with cold PBS. The pellets were then resuspended in 120 µL of EBC buffer B consisting of 50 mM Tris-HCl, pH 7.5, 300 mM NaCl, 0.5 mM EDTA, 2 μg/mL aprotinin, 20 mM NEM, 1 mM PMSF, 5 mM CaCl2, 50U micrococcal nuclease and incubated at room temperature for 10 min. The samples were then sonicated and centrifuged at 18,000 x g at 4 °C for 15 min. The supernatants were collected as the chromatin fractions.

### Gene knockdown by RNA interference

For siRNA-mediated knockdown, the 21-nucleotide siRNA duplexes with a 3’ dTdT overhang were synthesized by Dharmacon Inc (Lafayette, CO). The target sequences were 5’-GAACTAAAACAGCAGCAGA-3’ (GNL3 si1), 5’-AAGCTGTACTGCCAAGAAC-3’ (GNL3 si2), 5’-ACTCCGTACCAAGGGTTAT-3’ (SENP3 si1), 5’-CTGGCCCTGTCTCAGCCAT-3’ (SENP3 si2), 5’-TGTCCTGAGTGGAGAGAAT-3’ (USP36 si), 5’-GGAACCTGTCTCCACAAAG-3’ (BRCA1 si), and 5’- GGATGTTCTTAGCACATCA-3’ (BLM si). The control scrambled RNA was previously described^109^. These siRNA duplexes (100 nM) were introduced into cells using Lipofectamine 2000 (Invitrogen) following the manufacturer’s protocol. Cells were harvested at 48-72 hours post-transfection.

### Comet assay

Cells were trypsinized, pelleted at 200 g for 5 min, and resuspended in PBS. The cell suspension was mixed with molten 1% low melting agarose (LMA, 1:10 ratio), spread on glass slides coated with a 1% normal melting point agarose (NMA) layer, solidified at 4 °C for 10 min, and lysed with lysis buffer (2.5 M NaCl, 100 mM EDTA, 10 mM Tris base, 200 mM NaOH, 1% sodium lauryl sarcosinate, 1% Triton X-100) at 4 °C overnight in the dark. Slides were equilibrated in neutral electrophoresis buffer (100 mM Tris, 300 mM sodium acetate, pH 9.0) at 4 °C for 30 min and then electrophoresed at 1 V/cm (20 V, 400 mA) for 45 minutes. Slides were then sequentially dehydrated in 80% and 100% ethanol, 5 min each, respectively, and stained with SYBR Green (1:2000 in PBS, pH 7.4) for 15 min. After rinsing, slides were dried and imaged immediately. Tail moment was quantified from the images using ImageJ software.

### In vivo ubiquitination and SUMOylation assays

In vivo ubiquitination and SUMOylation assays under denaturing conditions were conducted using a Ni^2+^-NTA pull-down (PD) method^71^. For ubiquitination assay, cells were transfected with His-Ub and indicated plasmids and treated with 20 μM MG132 for 6 h before harvesting. The cells were harvested 48 h after transfection. A total of 20% of the cells were used for direct IB, while the remaining cells were lysed in lysis buffer containing 6 M guanidinium-HCl, 100 mM Na_2_HPO4/NaH_2_PO4, 10 mM Tris-HCl (pH 8.0), 10 mM imidazole and 10 mM β-mercaptoethanol (β-ME), followed by brief sonication to reduce viscosity. The cell lysates were then incubated Ni^2+^-NTA agarose beads for 4 h at room temperature. The beads were washed once each with lysis buffer, wash buffer I (8 M urea, 100 mM Na_2_HPO4/NaH_2_PO4, 10 mM β-ME, 10 mM Tris-HCl, pH 8.0), and wash buffer II (8 M urea, 100 mM Na_2_HPO4/NaH_2_PO4, 10 mM β-ME, 10 mM Tris-HCl, pH 6.3). The bead-bound proteins were eluted from the beads using elution buffer containing 150 mM Tris-HCl (pH 6.7), 150 mM NaCl, 10% Glycerol, 5% SDS, 300 mM imidazole and 0.72 M β-ME. The eluates were boiled in 1x SDS sample buffer and analyzed by IB. For the SUMOylation assay, cells were transfected with His-SUMO1 or His-SUMO2 and indicated plasmids, followed by Ni^2+^-NTA PD under denaturing conditions as above.

### In vitro SUMOylation assay

In vitro SUMOylation assay was carried out as described previously^71^ in a total of 20 μl reaction mixture containing SUMO E1 heterodimer (50 nM), Ubc9 (E2, 50 nM), SUMO2 (2 μM), ATP (2.5 mM), Flag-GNL3 (20 nM), and Flag-USP36 (0.25 μM) purified from HEK293 cells in reaction buffer consisting of 20 mM HEPES (pH 7.8), 100 mM NaCl, 5 mM MgCl_2_, 5% glycerol, and 1 mM DTT. The reactions were incubated at 30 °C for 4 h and then stopped by adding 7μl of 4x SDS sample buffer, followed by IB.

### Affinity purification and mass spectrometry

293 cells stably expressing control or Flag-GNL3 were lysed in NP40 lysis buffer supplemented with protease inhibitors at 4 °C for one hour followed by centrifugation. Twenty milligrams of the cleared cell lysates from either control or Flag-GNL3 expressing cells were incubated with 0.1 ml of anti-Flag (M2) agarose beads at 4 °C for 4 h. The beads were washed four times in lysis buffer containing protease inhibitors. The bead-bound proteins were eluted in 0.2 ml of TBS (50 mM Tris-HCl, 150 mM NaCl, pH 7.4) containing 0.1 mg/ml of Flag peptides. Eluted proteins were dried using vacuum centrifugation and resuspended in 75 µl of SDS protein extraction buffer (5% SDS, 50 mM TEAB). The samples were shaken at room temperature for 10 min and then reduced by adding 3.45 µl of 0.5 M dithiothreitol and incubated at 95 °C for 10 min. Samples were alkylated by the addition of 6.82 µl of 0.5 M iodoacetamide and incubation at room temperature for 30 min in the dark. Samples were then acidified by the addition of 8.53 µl of 12% aqueous phosphoric acid, and 562.8 µl of S-Trap protein binding buffer (90% aqueous methanol containing a final concentration of 100 mM triethylammonium bicarbonate (TEAB), pH 8) was added. An S-Trap micro column (Protifi, Farmingdale, NY) was placed into a 2.0 mL polypropylene tube to collect the flow-through for each sample. 165 µl of acidified SDS lysate/MeOH S-Trap buffer mixture from each sample was transferred to the S-trap micro column. Samples were centrifuged for 3 min at 4000 x g until all SDS lysate/S-Trap buffer had passed through the S-Trap column. Each S-Trap micro column was washed six times by adding 150 µl of S-Trap protein binding buffer, followed by centrifuge at 4000 x g for 3 min between each wash step. The S-trap columns containing the bound sample proteins were transferred to 2.0 mL Lobind centrifuge tubes, and 40 µL of 80 ng/µL sequencing-grade modified trypsin (Thermo Fisher, Waltham, MA) in 50 mM TEAB was added. S-trap columns were loosely capped, and digestion was performed at 37 °C overnight in a humidified chamber.

Upon completion of digestion, peptides were eluted by sequential addition of 40 µl of 50 mM TEAB, 40 µl of 0.2% aqueous formic acid, 40 µl of 50% acetonitrile, and 0.2% formic acid, with centrifugation at 4,000 xg for 4 min between each addition of elution buffer. The elution fractions were combined and dried using vacuum centrifugation. The dried peptides were reconstituted with 100 µL of HPLC-grade water and incubated for 15 min at 37 °C with shaking. Peptide concentrations were determined using the Pierce Quantitative Colorimetric Peptide Assay (Thermo Fisher). An equal volume of peptides from each sample were taken and dried using vacuum centrifugation. The dried peptides were then reconstituted by adding 20 µL of 5% formic acid in water and shaking at 37 °C for 15 min. The samples were injected onto an Acclaim PepMap 100 µm x 2 cm NanoViper C18, 5 µm trap (Pierce Thermo Fisher) at a 5 µL/minute flow rate on a switching valve. After 10 min of loading, the trap column was switched on-line to a PepMap RSLC C18, 2 µm, 75 µm x 25 cm EasySpray column (Pierce Thermo Fisher). Peptides from protein complexes were then separated using a 7.5–30% acetonitrile gradient over 55 min in the mobile phase containing 0.1% formic acid at a flow rate of 300 nL/min.

Tandem mass spectrometry data were collected using an Orbitrap Q-Exactive instrument (Thermo Scientific, San Jose, CA) configured with an EasySpray NanoSource. Survey scans were performed in the Orbitrap mass analyzer at a resolution of 120,000 with internal mass calibration enabled. The nanospray ionization source was operated with a spray voltage of 2400 V, and the ion transfer tube temperature was maintained at 300 °C. Full MS (MS1) survey scans were acquired in profile mode with a maximum injection time of 50 milliseconds and an automatic gain control (AGC) target of 3 × 10^5 ^ions. Dynamic exclusion was enabled, and charge states from 2 to 7 were included for fragmentation. The minimum AGC target threshold was 5 × 10^3 ^ions. Isotope exclusion was enabled and peptide matching was set to “preferred.” MS/MS (MS2) scans were also acquired in the Orbitrap analyzer. Precursor ions were isolated using a 1.2 m/z isolation window with no offset, followed by higher-energy collisional dissociation (HCD) fragmentation at a normalized collision energy of 30%. MS2 spectra were acquired at a resolution of 30,000 with a maximum injection time of 100 milliseconds, an AGC target of 1 × 10^4 ^ions, and a fixed first mass of 100 m/z. Data were recorded in centroid mode with a loop count of 10 MS2 scans per cycle.

Chromatographic separation was performed using a Dionex UltiMate NCS-3500RS system (Thermo Scientific, San Jose, CA). Mobile phase A consisted of 0.1% formic acid in water and mobile phase B consisted of 0.1% formic acid in acetonitrile. Peptides were initially trapped on a Thermo Acclaim PepMap C18 trap column (100 µm × 2 cm, NanoViper, 5 µm particle size) and washed for 10 minutes with mobile phase A. Following the wash step, peptides were separated on a Thermo PepMap RSLC C18 analytical column (75 µm ×  25 cm, EasySpray, 2 µm particle size). The flow rate delivered to the analytical column was maintained at 600 nL/min for a total run time was 90 min. A multi-step gradient elution profile was used to separate the peptides. From 0 to 10 min, the column was held at 2% mobile phase B to wash the samples on the trap column. At 10 min, the solvent composition was increased to 7.5% mobile phase B, followed by a linear gradient to 30% B over 55 min. The gradient was then increased to 98% mobile phase B for 5 min to wash the column. The mobile phase B composition was reduced back to 2% mobile phase B at 70 min, and the analytical column was equilibrated until the end of the run at 90 minutes.

Proteome UP000005640 (*homo sapiens*, taxon ID 9606) canonical FASTA sequences (20,652 proteins, one protein per gene) were downloaded June 2024 from www.UniProt.org. Common contaminants (175 sequences) were added, and sequence-reversed entries were concatenated for a final protein FASTA file of 41,654 sequences. The 24 binary instrument files were processed with the PAW pipeline^110^. RAW files were converted to text files using MSConvert^111^. Python scripts extracted fragment ion spectra in MS2 format^112^. The Comet search engine (version 2016.03)^113^ was used: 1.25 Da monoisotopic peptide mass tolerance, 0.02 Da monoisotopic fragment ion tolerance, 0.0 bin offset, fully tryptic cleavage with up to three missed cleavages, variable oxidation ( + 15.9949 Da) of methionine, and static alkylation of cysteines ( + 57.0215 Da). Top-scoring peptide spectrum matches (PSMs) were filtered to a 1% false discovery rate (FDR) using interactive delta-mass and conditional Peptide-prophet-like linear discriminant function scores^114^. Incorrect delta-mass and score histogram distributions were estimated using the target/decoy method^115^. The filtered PSMs were assembled into protein lists using basic and extended parsimony principles and required two distinct peptides per protein. The average number of proteins identified per sample was 1,460 + /− 60. Significance was determined by a two-tailed Student’s T-test statistical comparison between Flag-GNL3 and control groups (*n* = 3 biological replicates) after normalization of the spectral count data. Shared peptide spectral counts were fractionally split based on relative unique proteins spectral counts of the parent proteins containing the common peptides (corrected spectral counts).

### Cell viability and Colony formation assays

Cell viability was measured by 3-(4,5-dimethylthiazol-2-yl)−2,5-diphenyltetrazolium bromide (MTT) assays. Briefly, cells were incubated with 0.5 mg/ml MTT in medium for 3 h. After incubation, MTT medium was removed and dimethyl sulfoxide (DMSO, 100 μl per well) was added to fully dissolve the purple formazan. The absorbance was measured at OD560nm and OD690nm. The reduced absorbance (Abs560nm-Abs690nm) represents the relative number of viable cells per well. Colony formation was used to measure cell proliferation. Cells were seeded into 60 mm plates at a density of 2500 cells per plate and cultured in DMEM containing 10% FBS for up to 2 weeks. The colonies were visualized by staining with 0.5% crystal violet in 50% ethanol at room temperature for 2 h. Captured digital images of colonies were further processed and quantified using the ImageJ software.

### Proximity ligation assay

Proximity ligation assays were performed using the Duolink® PLA Fluorescence Kit (Sigma-Aldrich) according to the manufacturer’s instructions, with minor modifications. Briefly, cells cultured in coverslips were fixed with 4% paraformaldehyde for 15 min, permeabilized with 0.25% Triton X-100 for 15 min and blocked with Duolink® blocking solution at 37 °C for 1 h. Cells were then incubated with primary antibodies at 4 °C overnight. After washing with Duolink® In Situ Wash Buffer A, PLUS and MINUS PLA probes were diluted 1:5 in Duolink® antibody diluent buffer and applied to the cells, followed by incubation at 37 °C for 1 h. The cells were then washed with Wash Buffer A, and ligation was performed by adding ligase to 1× ligation buffer at a 1:40 dilution, followed by incubation in a pre-heated humidity chamber at 37 °C for 30 min. After additional washing with Wash Buffer A, amplification was carried out by adding polymerase to 1× amplification buffer at a 1:80 dilution, followed by incubation and subsequent washing with Wash Buffer B. Finally, the coverslips were mounted with Duolink® In Situ Mounting Medium containing DAPI. Samples were allowed to equilibrate for 15 min before imaging with a confocal microscope.

### DNA damage in situ ligation followed by PLA (DI-PLA)

This protocol is adapted from Galbiati et al^84^. Cells were grown on 13 mm coverslips and fixed in 4% PFA for 10 minutes at room temperature, followed by two washes with PBS. For DSB end blunting, coverslips were washed twice for 5 min with NEB2 buffer (50 mM NaCl, 10 mM Tris-HCl pH 8, 10 mM MgCl_2_, 1 mM DTT, 0.1% Triton X-100) and twice for 5 min with blunting buffer (50 mM NaCl, 10 mM Tris-HCl pH 7.5, 10 mM MgCl_2_, 5 mM DTT, 0.025% Triton X-100). Coverslips were then inverted onto a 35μL drop of NEB Blunting Reaction mixture (NEB, E1201) on parafilm containing 1 mM dNTPs, 1X Blunting Buffer, 0.2 mg/mL BSA, 1X Blunting Enzyme, and incubated in a dark humidity-controlled chamber for one hour at room temperature. For DSB end Ligation, coverslips were washed twice for 5 minutes with NEB2 buffer and twice for 5 minutes with ligation buffer (50 mM Tris-HCl pH 7.5, 10 mM MgCl_2_, 10 mM DTT, 1 mM ATP). Coverslips were then inverted onto a 50μL drop of Ligation Reaction (0.1μM DI-PLA Linker, 1X T4 Ligation Buffer (NEB, B0202), 1 mM ATP, 0.2 mg/mL BSA, 1X T4 Ligase (NEB, M0202)) on parafilm, and incubated overnight at 4 °C in a dark, humidity chamber, followed by proximity ligation assay between biotin and GNL3. The DI-PLA Linker sequence is 5’-TACTACCTCGAGAGTTACGCTAGGGATAACAGGGTAATATAGTTT [BtndT] TTTCTATATTACCCTGTTATCCCTAGCGTAACTCTCGAGGTAGTA −3’

### Immunofluorescence staining

Cells grown on coverslips were fixed with 4% paraformaldehyde, permeabilized with 0.25% Triton X-100, and blocked with 8% BSA. Cells were then incubated with primary antibodies, followed by staining with Alexa Fluor 488 (green) goat anti-mouse, Alexa Fluor 555 (red) goat anti-mouse, Alexa Fluor 488 (green) goat anti-rabbit, or Alexa Fluor 555 (red) goat anti-rabbit antibodies (Invitrogen), as well as DAPI for DNA staining (see listed antibodies in Supplementary Table 2). Stained cells were imaged by using a Zeiss ApoTome fluorescence microscope (Zeiss) or a Zeiss LSM980 confocal microscope (Zeiss).

### Image analysis

Confocal imaging was performed on a Zeiss LSM980 microscope equipped with a Plan-Apochromat 63 x/1.40 NA oil immersion objective lens (Zeiss). The following excitation and detection windows were used: DAPI, 405 nm excitation, 420–480 nm emission; Alexa Fluor 488, 488 nm excitation, 500–550 nm emission; Alexa Fluor 555, 561 nm excitation, 565–620 nm emission. PLA signals were quantified as the number of discrete nuclear dots per cell using ImageJ. DNA damage-induced foci were also quantified using ImageJ. For all PLA and DNA damage foci experiments, at least 100 cells were counted from at least three independent experiments.

### DNA End resection assay by BrdU labeling

Cells were seeded in 24-well plates at a density of 3-4 × 10^4^ cells per well and cultured in coverslips in the presence of 10 μM BrdU for 20 h. Cells were washed twice with PBS and incubated in pre-extraction buffer A (10 mM PIPES pH 7, 100 mM NaCl, 3 mM MgCl_2_, 1 mM EGTA, 0.5% Triton X-100 and 300 mM Sucrose) at 4 °C for 10 min. Cells were then incubated in cytoskeleton stripping buffer B (10 mM Tris pH 7.5, 10 mM NaCl, 3 mM MgCl_2_, 1% Tween 20, 0.50% sodium deoxycholate) at 4 °C for 10 min, followed by a single wash with PBS. Cells were fixed with 4% paraformaldehyde for 15 min, followed by incubation in 100% cold methanol at −20 °C for 5 min. After permeabilization with 0.5% Triton X-100 at room temperature for 15 min, cells were blocked in 3% BSA, and incubated with anti-BrdU and anti-GNL3 antibodies, followed by staining with Alexa Fluor 488 (green) goat anti-mouse, Alexa Fluor 555 (red) goat anti-rabbit antibodies along with DAPI for DNA staining. BrdU-positive cells were defined as nuclei containing ≥ 20 strong BrdU foci, based on previously published criteria^116,117^.

### ssDNA-PLA

ssDNA-PLA, a PLA-based approach for detecting protein association with ssDNA, was performed essentially as described^88^. Briefly, cells were labeled with 10 μM BrdU for 20 h, as described in the End Resection Assay. After fixation and permeabilization, PLA was performed using the Duolink® In Situ Red Starter Kit (Sigma-Aldrich) with anti-BrdU and anti-GNL3 antibodies following the manufacturer’s instructions. Slides were mounted using Duolink® In Situ Mounting Medium containing DAPI and imaged using a Zeiss LSM980 confocal microscope (Zeiss).

### DNA End resection assay by quantitative PCR

DNA end resection by qPCR was conducted essentially as described^85,118^. ER-*Asi*SI U2OS cells treated with 300 nM 4-OHT for 4 h were harvested, washed with ice-cold PBS and resuspended in 0.6% low-melting-point agarose in PBS prewarmed to 37 °C. Aliquots of 50 μL cell suspension were dropped onto Parafilm and allowed to solidify at 4 °C for 5–10 min. The resulting agarose plugs were transferred individually into 1.5 mL tubes containing 1 mL ESP buffer (0.5 M EDTA, 2% N-lauroylsarcosine, 1 mM CaCl₂, and 1 mg/mL proteinase K) and incubated for 20 h at 16 °C with rotation. The ESP buffer was then replaced with 1 mL HS buffer (1.85 M NaCl, 0.15 M KCl, 5 mM MgCl₂, 2 mM EDTA, 4 mM Tris-HCl pH 7.5, and 0.5% Triton X-100), and samples were incubated for an additional 20 h at 16 °C with rotation. The agarose plugs were then washed six times with ice-cold PBS for 10 min per wash at 4 °C. Plugs were then melted at 70 °C for 10 minutes and diluted 15-fold with prewarmed ddH₂O. Samples were gently mixed by pipetting to avoid shearing genomic DNA. After cooling to room temperature, an equal volume of 2× rCutSmart NEB buffer was added, and DNA concentration was measured using a NanoDrop spectrophotometer. Genomic DNA was purified using the DNeasy Blood & Tissue Kit (Qiagen) according to the manufacturer’s instructions, including RNase A treatment. A total of 140 ng genomic DNA was incubated with 20 U BsrGI-HF in 1× rCutSmart buffer at 37 °C for 12–16 h or mock-digested, then heat-inactivated at 65 °C, followed by qPCR analysis. DNA end resection was measured using primer pairs located 335 bp and 1618 bp from the chromosome 1 DSB site, respectively. Primer sequences for DSB (335 bp) were 5′- GAATCGGATGTATGCGACTGATC −3′, and 5′- TTCCAAAGTTATTCCAACCCGAT −3′. Primer sequences for DSB (1618 bp) were 5′- TGAGGAGGTGACATTAGAACTCAGA −3′, and 5′- AGGACTCACTTACACGGCCTTT −3′. ΔCt was determined as the difference between the Ct value of the restriction enzyme-digested sample and that of the corresponding mock-digested control. The percentage of single-stranded DNA was calculated as follows: ssDNA%=1/(2^(ΔCt-1) + 0.5)*100.

#### Chromatin Immunoprecipitation (ChIP)-qPCR

ChIP analysis was performed essentially as described^119,120^. ER-*Asi*SI U2OS cells transfected with control or Flag-GNL3 plasmids were cultured in the absence or presence of 300 nM 4-OHT for 4 h. Cells were then cross-linked with a 1% formaldehyde solution for 10 min, followed by incubation in 0.125 M glycine (final concentration) for 5 min. Cells were harvested and lysed in ChIP lysis buffer consisting of 150 mM NaCl, 1% Nonidet P-40, 0.5% deoxycholate, 0.1% SDS, 50 mM Tris (pH 8.0), 5 mM EDTA and protease inhibitors. Cell lysates were sonicated to yield chromatin fragments of less than 1000 bp. After centrifugation to remove the cell debris, cell lysates were incubated with control mouse IgG or anti-Flag antibody for 4 h at 4°C, followed by incubation with protein G beads for 2 h. The immunoprecipitates were washed with lysis buffer for 4 times and ChIP wash buffer (100 mM Tris [pH 8.5], 500 mM LiCl, 1% NP-40, 1% deoxycholic acid) for 4 times, followed by elution with ChIP elution buffer (50 mM NaHCO3, 1% SDS). The elutes were treated with RNAse A, followed by reversing cross-link with 0.2 M NaCl and Protease K at 65 °C for 6 h. Immunoprecipitated chromatin DNA was then purified using a DNA purification kit (Cell Signaling Technology) and analyzed for promoter occupancy by qPCR amplification. The primers for amplifying a DNA region near a *Asi*SI cleavage site located in chromosome 1 were 5’-CTGGAAGAGACTGGATGAGGG-3’ and 5’- AGTCACACTCCACAGCCAA −3’.

### HR and NHEJ reporter assays

U2OS Cells were transfected with scr, GNL3 siRNA, USP36 siRNA, or SENP3 siRNA together with pCBA-I-SceI, pCherry, and either DR-GFP or PimEJ5GFP plasmids. Cells were harvested by trypsinization and fixed at 48 h post-transfection and analyzed by flow cytometry for the percentage of mCherry/GFP positive cells. mCherry expression was used to normalize transfection efficiency.

### TCGA data analysis

RNA-seq and clinical data from The Cancer Genome Atlas (TCGA) were retrieved using the R package TCGAbiolinks (v2.30.4). Gene-level expression was quantified as transcripts per million (TPM) and transformed as log1p(TPM) prior to analysis. Triple-negative breast cancer (TNBC) status was defined from TCGA receptor annotations for ER, PR, and HER2 negative; cases missing any receptor annotation were excluded. Group differences were assessed with two-sided Wilcoxon rank-sum tests.

### Statistics and reproducibility

Measurements and cell counts were performed using ImageJ. Results are expressed as the mean ± SD unless otherwise specified. Statistical analyses were performed using GraphPad Prism. An unpaired two-tailed Student’s *t*test was employed to determine the differences between the two groups. One-way ANOVA was used for comparison among multiple groups with a single factor, whereas two-way ANOVA was employed to determine statistical differences among multiple groups with two factors, followed by Tukey’s multiple comparisons test. *P* values are indicated in figures or figure legends, and *p* < 0.05 was considered statistically significant. All experiments were repeated in multiple independent experiments as indicated in the figure legends. All immunoblots and images shown are representative of these independent experiments. In all cases, independent replication experiments produced similar results.

### Reporting summary

Further information on research design is available in the Nature Portfolio Reporting Summary linked to this article.

## Supplementary information

Supplementary information

Transparent Peer Review file

Reporting Summary

Description of Additional Supplementary Files

Supplementary Data 1

Supplementary Data 2

Supplementary Data 3

Supplementary Data 4

## Source data

Source Data

## Data Availability

The mass spectrometry proteomics data generated in this study have been deposited to the ProteomeXchange Consortium via the PRIDE partner repository with the dataset identifier PXD068465 (https://www.ebi.ac.uk/pride/archive/projects/PXD068465). GNL3 expression data for breast cancer were obtained from TCGA via the Genomic Data Commons Data Portal (https://portal.gdc.cancer.gov/). GNL3 protein expression data for breast cancers were obtained from CPTAC via UALCAN (https://ualcan.path.uab.edu/analysis-prot.html). Survival data were obtained from Kaplan–Meier plotter (http:// kmplot.com/analysis/). GNL3 variants data in breast cancers were obtained from cBioportal (https://www.cbioportal.org/). All data generated or analyzed in this study are included in this article and its Supplementary Information files. All relevant raw data from figures generated in this study have been provided in the Source Data file. Source data are provided with this paper.
