# Supplementary material for: GNL3 SUMOylation is essential for DNA double-strand break repair by homologous recombination
Source: Nat Commun. 2026 Aug 24;17:10122. doi: 10.1038/s41467-026-77013-1 (PMC13598120; doi:10.1038/s41467-026-77013-1)
Supplement: Supplementary file 1 [file 41467_2026_77013_MOESM1_ESM.pdf]

Supplementary Information

GNL3 SUMOylation is essential for DNA double-strand break repair by homologous recombination

Yunhan Yang[1], Yanping Li[1], Roselyn S. Dai[1], Canping Chen[2], Keith Zientek[3], Ashok Reddy[3], Zheng Xia[2,4,5], Rosalie C. Sears[1,5,6], Xiao-Xin Sun[1*,] Mu-Shui Dai[1,5,6*]

[1]Department of Molecular & Medical Genetics, School of Medicine, [2]Department of Biomedical Engineering, [3]OHSU Proteomics Shared Resource, [4]Center for Biomedical Data Science, [5]OHSU Knight Cancer Institute, and [6]Brenden-Colson Center for Pancreatic Care, Oregon Health & Science University, 3181 SW Sam Jackson Park Road, Portland, OR 97239, USA

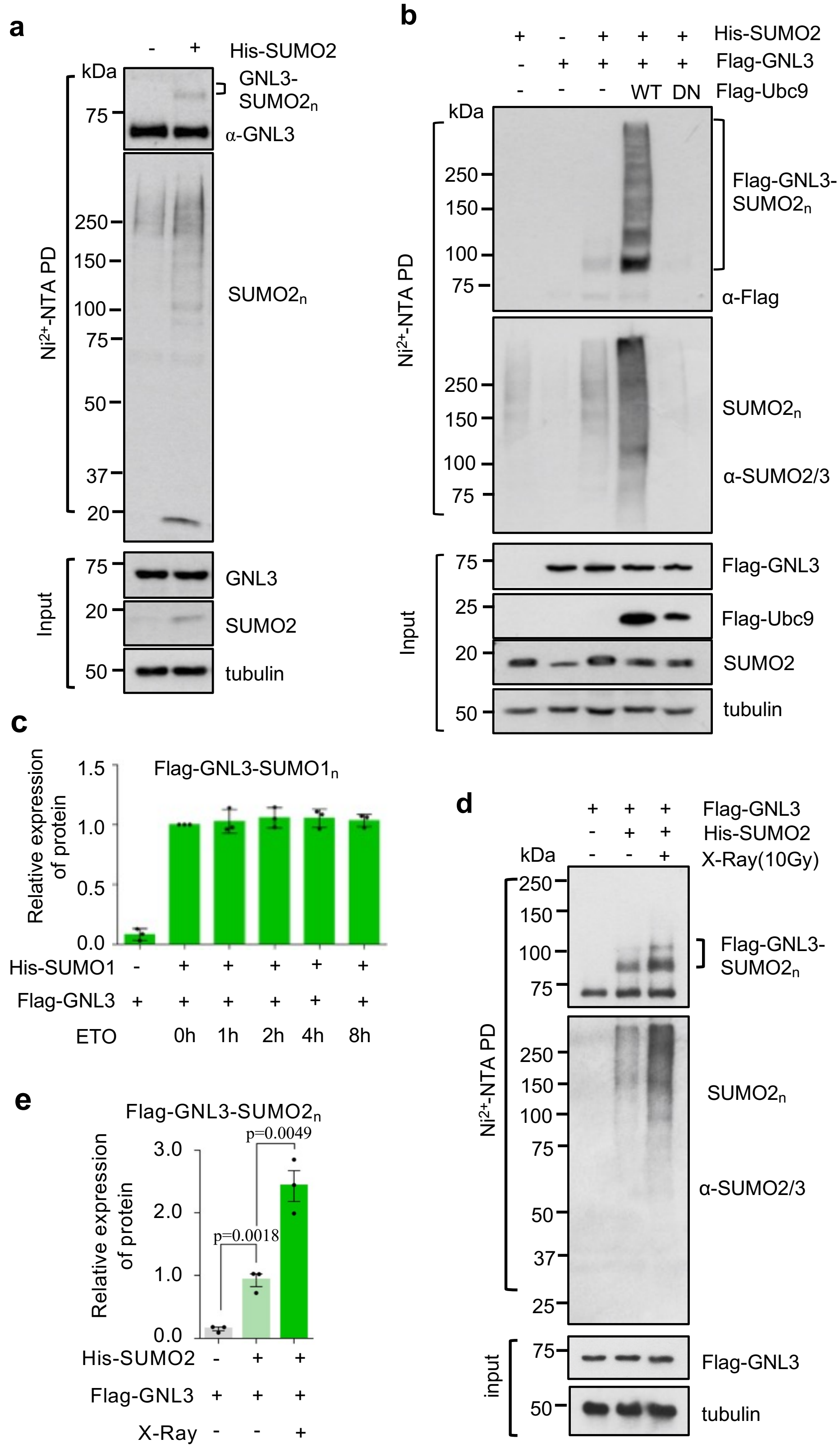
a
- +
His-SUMO2
kDa
75
GNL3-SUMO2n
α-GNL3
250
150
100
75
50
37
20
SUMO2n
Ni2+-NTA PD
75
GNL3
20
SUMO2
50
tubulin
Input
b
His-SUMO2
Flag-GNL3
Flag-Ubc9
WT
DN
kDa
250
150
100
75
Flag-GNL3-SUMO2n
α-Flag
250
150
100
75
SUMO2n
α-SUMO2/3
Ni2+-NTA PD
75
Flag-GNL3
25
Flag-Ubc9
20
SUMO2
50
tubulin
Input
c
Flag-GNL3-SUMO1n
Relative expression of protein
1.5
1.0
0.5
0.0
His-SUMO1
Flag-GNL3
ETO
0h
1h
2h
4h
8h
d
Flag-GNL3
His-SUMO2
X-Ray(10Gy)
kDa
250
150
100
75
Flag-GNL3-SUMO2n
250
150
100
75
50
37
25
SUMO2n
α-SUMO2/3
Ni2+-NTA PD
75
Flag-GNL3
50
tubulin
input
e
Flag-GNL3-SUMO2n
Relative expresion of protein
3.0
2.0
1.0
0.0
p=0.0049
p=0.0018
His-SUMO2
Flag-GNL3
X-Ray

Supplementary Figure S1. DNA damage induces GNL3 SUMOylation. (a) SUMOylation of endogenous GNL3 by SUMO2. HeLa cells transfected with control or His-SUMO2 were subjected to Ni^{2+}-NTA pull down (PD) under denaturing conditions, followed by IB. n=2 independent experiments. **(b)** Overexpression of WT Ubc9, but not its dominant-negative (DN) mutant, promotes GNL3 SUMOylation. H1299 cells transfected with the indicated plasmids were subjected to Ni^{2+}-NTA PD under denaturing conditions, followed by IB. The SUMO2 modified GNL3 and the expression of total SUMO2 are indicated. The protein expression is shown in the bottom panels. n=3 independent experiments. Error bars denote mean ± SD. **(c)** Quantification of SUMO1 modified GNL3 in response to ETO treatment (Fig. 1g) from three independent experiments. Error bars denote mean ± SD. **(d)(e)** GNL3 SUMOylation is increased in response to X-ray irradiation. U2OS cells stably expressing His-SUMO2 were treated with or without 10 Gy X-ray for 2 hours and assayed for GNL3 SUMOylation. Quantification of SUMOylated GNL3 relative to total GNL3 protein from three independent experiments is shown in (e). Significance was determined by unpaired two-tailed Student's t-test.

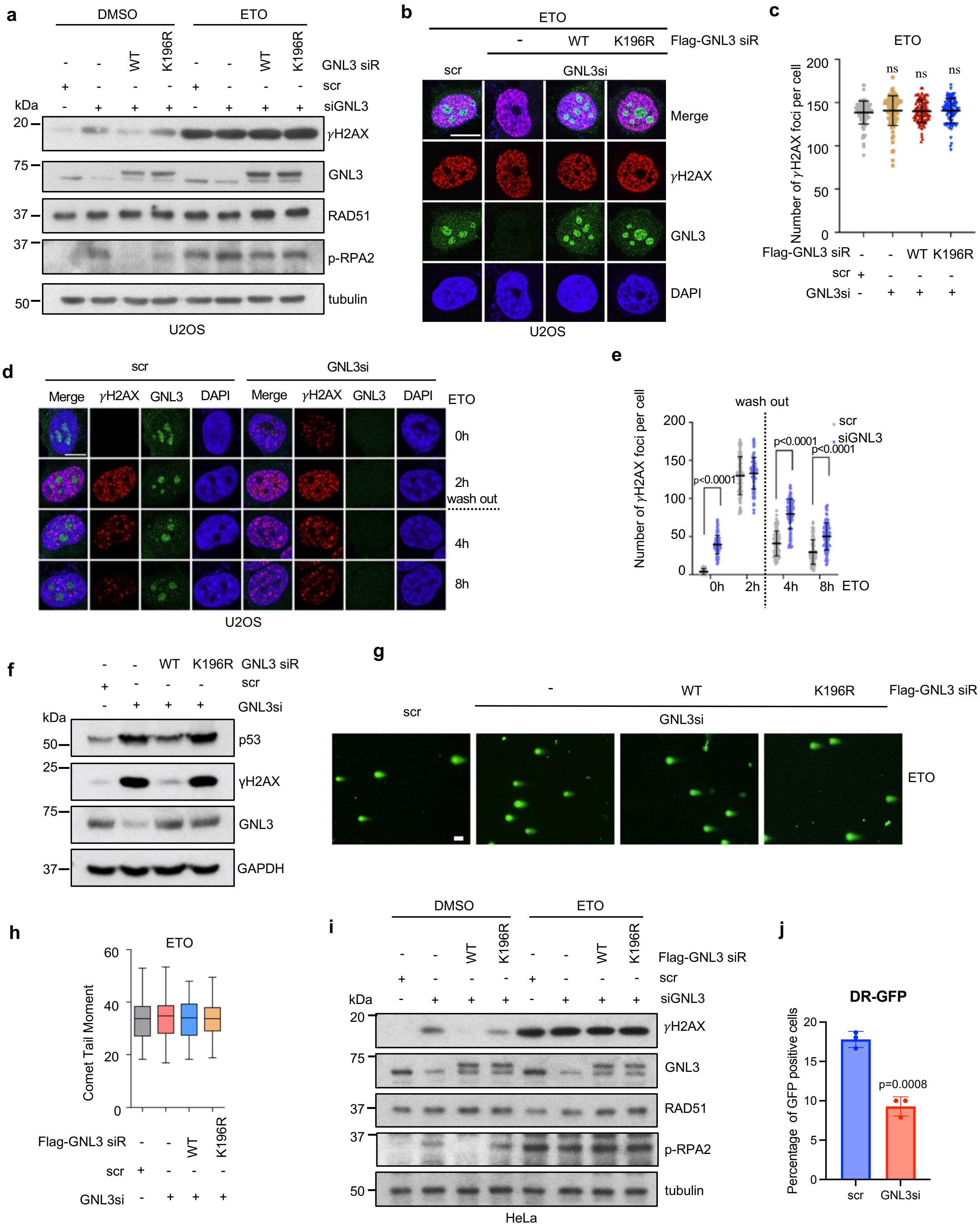
a
DMSO
ETO
- - WT K196R - - WT K196R GNL3 siR
+ - - - + - - - scr
kDa
- + + + - + + + siGNL3
20
γH2AX
75
GNL3
37
RAD51
37
p-RPA2
50
tubulin
U2OS
b
ETO
- WT K196R Flag-GNL3 siR
scr
GNL3si
Merge
γH2AX
GNL3
DAPI
U2OS
c
ETO
ns ns ns
200
150
100
50
0
Number of γH2AX foci per cell
Flag-GNL3 siR - - WT K196R
scr + - - -
GNL3si - + + +
d
scr
GNL3si
Merge γH2AX GNL3 DAPI Merge γH2AX GNL3 DAPI
ETO
0h
2h
wash out
4h
8h
U2OS
e
wash out
scr
siGNL3
200
150
100
50
0
p<0.0001
p<0.0001
p<0.0001
Number of γH2AX foci per cell
0h 2h 4h 8h ETO
f
- - WT K196R GNL3 siR
+ - - - scr
- + + + GNL3si
kDa
50
p53
25
γH2AX
75
GNL3
37
GAPDH
g
- WT K196R Flag-GNL3 siR
scr
GNL3si
ETO
h
ETO
60
40
20
0
Comet Tail Moment
Flag-GNL3 siR - - WT K196R
scr + - - -
GNL3si - + + +
i
DMSO
ETO
- - WT K196R - - WT K196R Flag-GNL3 siR
+ - - - + - - - scr
kDa
- + + + - + + + siGNL3
20
γH2AX
75
GNL3
37
RAD51
37
p-RPA2
50
tubulin
HeLa
j
DR-GFP
20
15
10
5
0
p=0.0008
Percentage of GFP positive cells
scr GNL3si

Supplementary Figure S2. GNL3 SUMOylation at K196 is essential for DDR. (a) U2OS cells transfected with scrambled (scr) or GNL3 siRNA, together with siRNA-resistant (siR) WT GNL3 or the K196R mutant, were treated with DMSO or 20 μM ETO for 2 hours and analyzed by IB. n=2 independent experiments. **(b)(c)** U2OS cells transfected with scr or GNL3 siRNA together with siRNA-resistant Flag-GNL3 (WT or K196R) were treated with 20 μM ETO for 2 hours and assayed by IF staining with anti-GNL3 and anti-γH2AX antibodies. Shown are representative confocal images (b) and the quantification (c) from three independent experiments (n=100 cells/condition). Error bars denote mean ± SD. Significance was determined by one-way ANOVA Tukey's multiple comparisons test. ns, not significant**. (d)(e)** U2OS cells transfected with scr or GNL3 siRNA were treated with or without 20 μM ETO for 2 hours, then washed and replaced with fresh medium, followed by IF with anti-γH2AX and anti-GNL3 antibodies. Shown are representative confocal images (d) and quantification of the γH2AX foci (e) from three independent experiments (n=100 cells/condition). Significance was determined by unpaired two-tailed t-test. **(f)** U2OS cells transfected with the indicated siRNA and plasmids were assayed by IB. n=2 independent experiments. **(g)(h)** Comet assays in U2OS cells transfected with scr or GNL3 siRNA together with siRNA-resistant Flag-GNL3 (WT or K196R) and treated with 20 μM ETO for 2 hours. Shown are representative images (g) and the quantification (h) from three independent experiments (n=100 cells/condition). The center line of the box plots represents the median value (50th percentile), and the box encapsulates the interquartile range. The whiskers extend to show the rest of the distribution. **(i)** HeLa cells transfected with the indicated siRNA and plasmids were treated with DMSO or 20 μM ETO for 2 hours and assayed by IB. n=3 independent experiments. (**j**) U2OS cells transfected with scr or GNL3 siRNA, together with HR reporter plasmids, were analyzed by flow cytometry to determine the percentage of GFP-positive cells. Significance was determined by unpaired two-tailed t-test. n=3 independent experiments. Scale bar, 10 μm. Error bars in **c**,**e**,**h**,**j** denote mean ± SD.

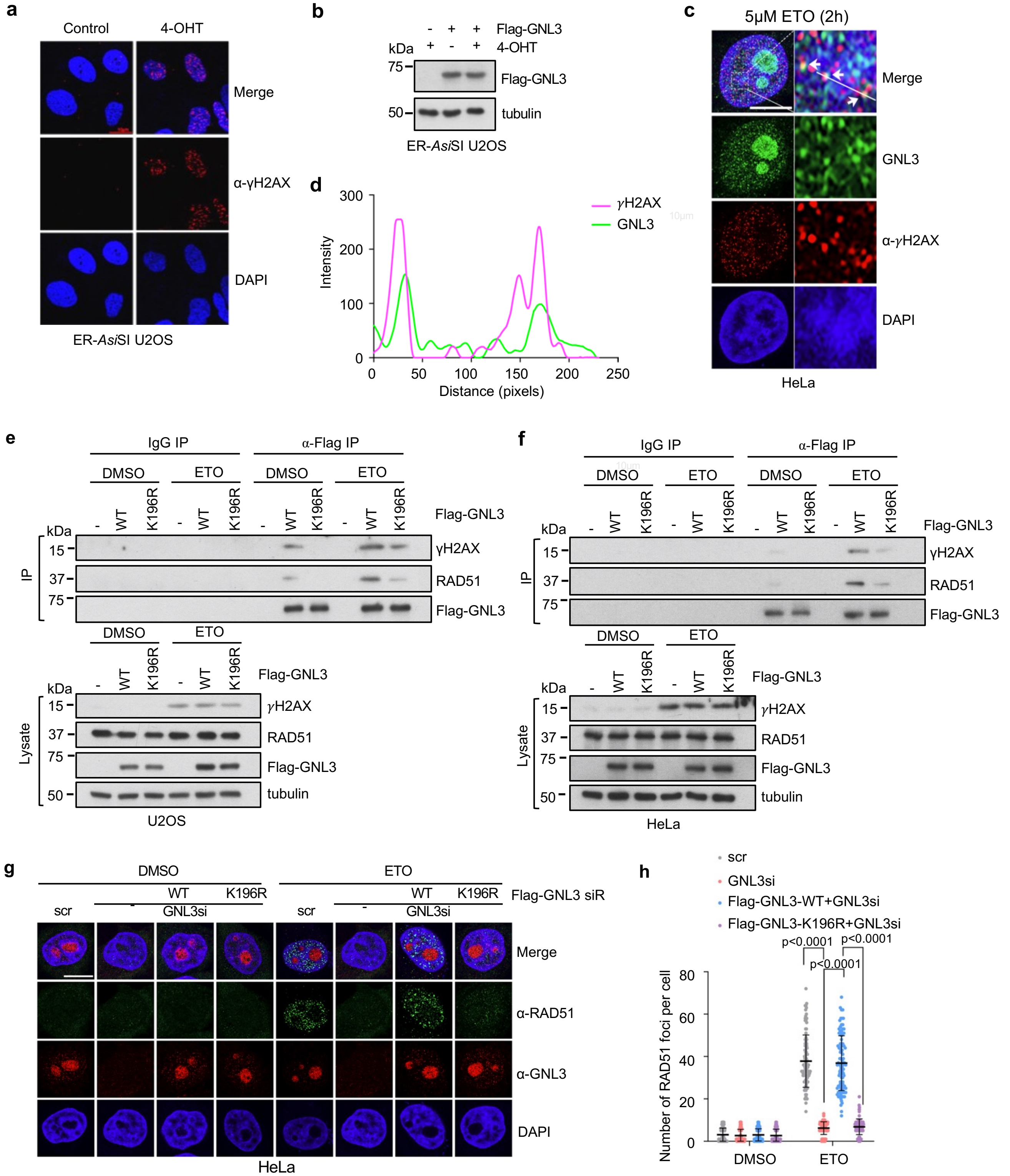
a
Control
4-OHT
Merge
α-γH2AX
DAPI
ER-AsiSI U2OS
b
Flag-GNL3
4-OHT
kDa
75
50
Flag-GNL3
tubulin
ER-AsiSI U2OS
c
5μM ETO (2h)
Merge
GNL3
α-γH2AX
DAPI
HeLa
d
γH2AX
GNL3
Intensity
Distance (pixels)
e
IgG IP
α-Flag IP
DMSO
ETO
WT
K196R
Flag-GNL3
IP
γH2AX
RAD51
Lysate
tubulin
U2OS
f
HeLa
g
DMSO
ETO
scr
GNL3si
Flag-GNL3 siR
α-RAD51
α-GNL3
h
scr
GNL3si
Flag-GNL3-WT+GNL3si
Flag-GNL3-K196R+GNL3si
p<0.0001
Number of RAD51 foci per cell

Supplementary Figure S3. GNL3 associates with DNA damage sites and its SUMOylation at K196 is critical for DDR. (a) ER-*Asi*SI U2OS cells were treated with control ethanol or 4-OHT for 4 hours, followed by IF staining for γH2AX foci using anti-γH2AX antibody. n=2 independent experiments. **(b)** Expression of Flag-GNL3 in the experiment shown in Fig. 3f. ER-*Asi*SI U2OS cells transfected with control or Flag-GNL3 plasmid were analyzed by IB. n=3 independent experiments. **(c)** GNL3 colocalizes with γH2AX foci. HeLa cells were treated with 5 μM ETO for 2 hours, followed by IF staining with anti-GNL3 and anti-γH2AX antibodies. Zoomed-in views of a nucleoplasm region are shown in the right panels. **(d)** Plot showing fluorescence intensity at each pixel along the line drawn from left to right in the upper-right image (c) (green, GNL3; magenta, γH2AX). **(e)(f)** GNL3 SUMOylation is critical for its interaction with γH2AX and RAD51 in response to DNA damage. U2OS (e) and HeLa (f) cells transfected with control, WT Flag-GNL3 or the K196R plasmid were treated with DMSO or 20 μM ETO for 2 hours. Cells were immunoprecipitated with control mouse IgG or anti-Flag antibody, followed by IB. n=2 independent experiments. **(g)(h)** GNL3 SUMOylation is critical for RAD51 foci formation upon DNA damage. HeLa cells transfected with scr or GNL3 siRNA, together with siRNA-resistant WT Flag-GNL3 or the K196R mutant, were treated with DMSO or 5 μM ETO for 2 hours and analyzed for RAD51 foci formation by IF. Shown are representative confocal images (g) and quantification (h) from three independent experiments (n=100 cells/condition). Scale bar, 10 μm. Error bars denote mean ± SD. Significance was determined by unpaired two-tailed t-test.

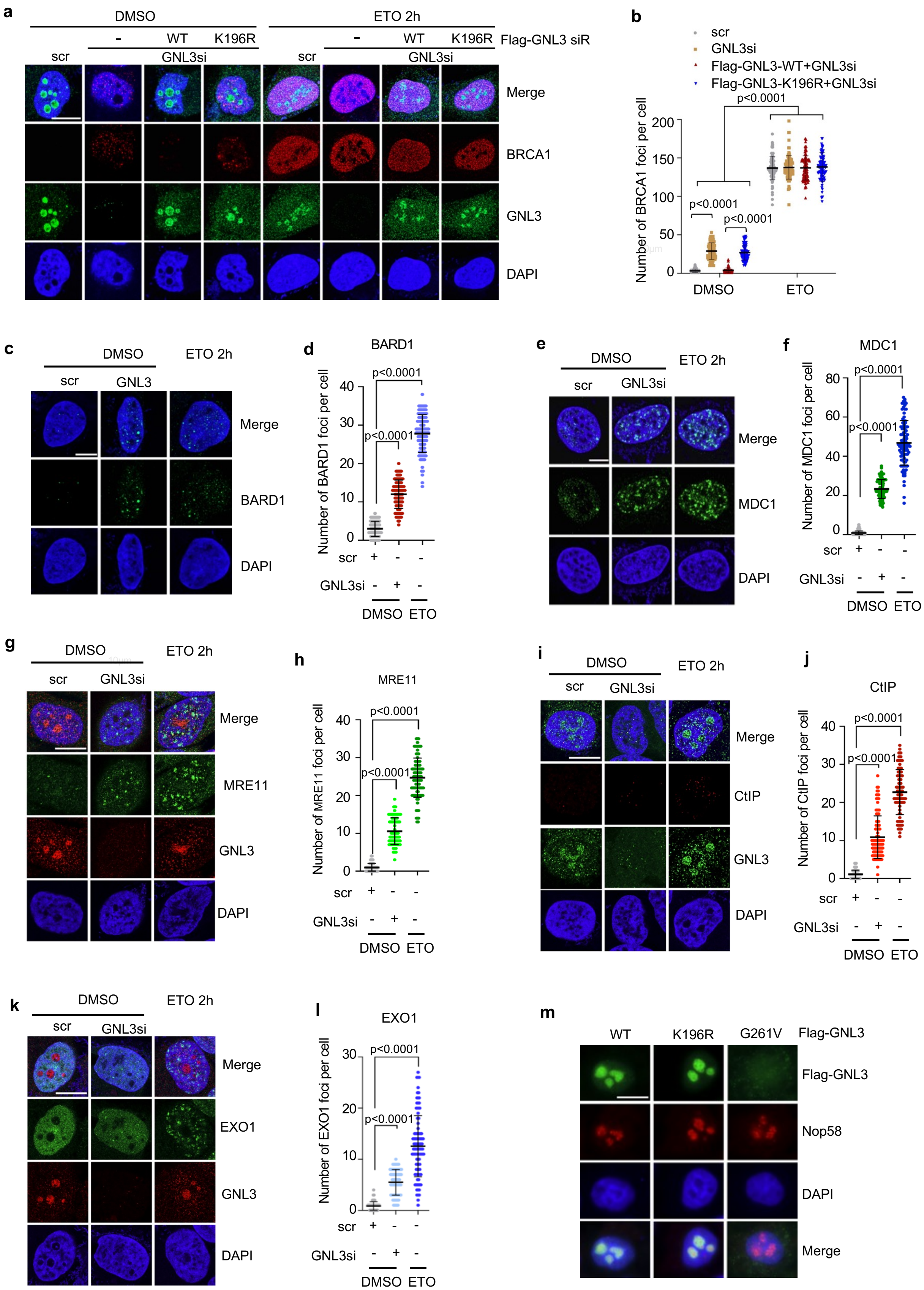
a
DMSO
ETO 2h
-
WT
K196R
Flag-GNL3 siR
scr
GNL3si
Merge
BRCA1
GNL3
DAPI
b
scr
GNL3si
Flag-GNL3-WT+GNL3si
Flag-GNL3-K196R+GNL3si
Number of BRCA1 foci per cell
p<0.0001
0
50
100
150
200
DMSO
ETO
c
DMSO
ETO 2h
scr
GNL3
Merge
BARD1
DAPI
d
BARD1
Number of BARD1 foci per cell
p<0.0001
0
10
20
30
40
scr
GNL3si
DMSO
ETO
e
DMSO
ETO 2h
scr
GNL3si
Merge
MDC1
DAPI
f
MDC1
Number of MDC1 foci per cell
p<0.0001
0
20
40
60
80
scr
GNL3si
DMSO
ETO
g
DMSO
ETO 2h
scr
GNL3si
Merge
MRE11
GNL3
DAPI
h
MRE11
Number of MRE11 foci per cell
p<0.0001
0
10
20
30
40
scr
GNL3si
DMSO
ETO
i
DMSO
ETO 2h
scr
GNL3si
Merge
CtIP
GNL3
DAPI
j
CtIP
Number of CtIP foci per cell
p<0.0001
0
10
20
30
40
scr
GNL3si
DMSO
ETO
k
DMSO
ETO 2h
scr
GNL3si
Merge
EXO1
GNL3
DAPI
l
EXO1
Number of EXO1 foci per cell
p<0.0001
0
10
20
30
scr
GNL3si
DMSO
ETO
m
WT
K196R
G261V
Flag-GNL3
Flag-GNL3
Nop58
DAPI
Merge

Supplementary Figure S4. GNL3 knockdown does not affect the formation of BRCA1, BARD1, MDC1, MRE11, CtIP and EXO1 foci. (a)(b) U2OS cells transfected with scr or GNL3 siRNA, together with Flag-tagged, siRNA-resistant WT GNL3 or the K196R mutant, were treated with DMSO or 20 μM ETO for 2 hours and assayed for BRCA1 foci formation by IF with anti-BRCA1 and anti-GNL3 antibodies. Shown are representative confocal images (a) and quantification (b) of n=100 cells/condition from three independent experiments. Error bars denote mean ± SD. Significance was determined by two-way ANOVA, Tukey's multiple comparisons test. **(c)-(l)** U2OS cells transfected with scr or GNL3 siRNA for 48 hours or treated with 20 μM ETO for 2 hours were assayed by IF using anti-BARD1 (c)(d), anti-MDC1 (e)(f), anti-MRE11 (g)(h), anti-CtIP (i)(j), and anti-EXO1 (k)(l) antibodies. Shown are representative confocal images (**c**,**e**,**g,I**,**k**) and the quantification (**d**,**f**,**h**,**j**,**l**) of n=100 cells/condition from three independent experiments. Error bars denote mean ± SD. Significance was determined by one-way ANOVA, Tukey's multiple comparisons test. **(m)** HeLa cells transfected with the indicated plasmids were assayed by IF staining with anti-Flag and anti-Nop58 (a nucleolar marker) antibodies. n=2 independent experiments. Scale bars, 10 μm.

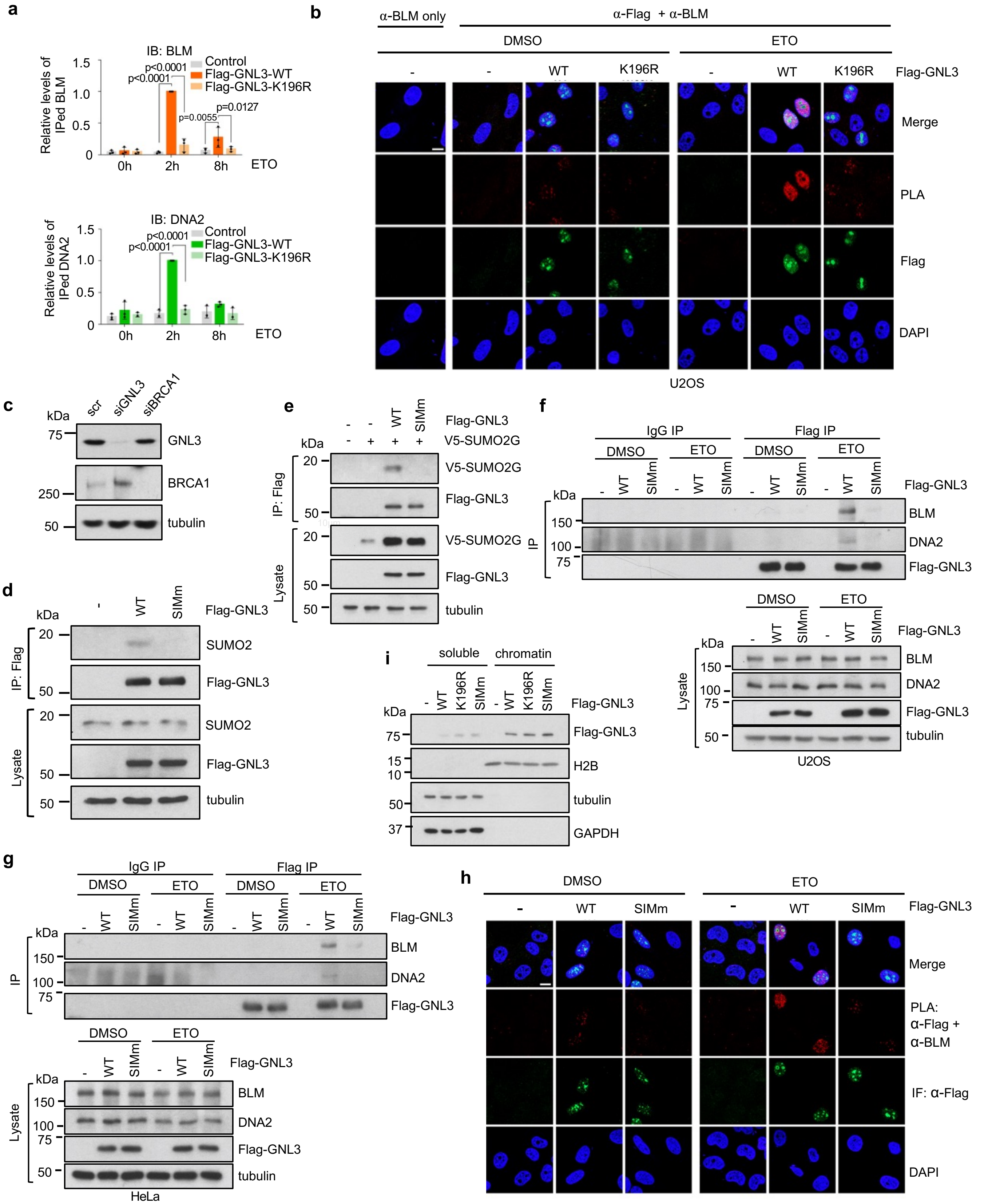
a
IB: BLM
Relative levels of IPed BLM
Control
Flag-GNL3-WT
Flag-GNL3-K196R
p<0.0001
p<0.0001
p=0.0055
p=0.0127
0h
2h
8h
ETO
IB: DNA2
Relative levels of IPed DNA2
b
α-BLM only
α-Flag + α-BLM
DMSO
ETO
WT
K196R
Flag-GNL3
Merge
PLA
Flag
DAPI
U2OS
c
scr
siGNL3
siBRCA1
kDa
GNL3
BRCA1
tubulin
d
SUMO2
Flag-GNL3
IP: Flag
Lysate
SIMm
e
V5-SUMO2G
f
IgG IP
Flag IP
BLM
DNA2
IP
g
HeLa
h
PLA:
α-Flag +
α-BLM
IF: α-Flag
i
soluble
chromatin
H2B
GAPDH

Supplementary Figure S5. GNL3 regulates DNA end resection by interacting with the BLM-DNA2 complex. (a) Quantification of immunoprecipitated BLM and DNA2 from three biological replicates of Fig. 4b. Error bars denote mean ± SD. Significance was determined by unpaired two-tailed Student's t-test. **(b)** Representative wide-field views of confocal images from the experiments shown in Fig. 4h. Scale bar, 10 μm. **(c)** Representative results of protein expression for Fig. 4o. ER-*Asi*SI U2OS cells transfected with scr, GNL3 siRNA or BRCA1 siRNA were treated with 4-OHT for 4 hours, followed by IB. n=3 independent experiments**. (d)** H1299 cells transfected with WT Flag-GNL3 or the SIM mutant (SIMm) were assayed by co-IP. n=2 independent experiments. **(e)** U2OS cells transfected with WT Flag-GNL3 or the SIM mutant together with V5-SUMO2G were assayed by co-IP, followed by IB. n=2 independent experiments. **(f)(g)** U2OS (f) and HeLa (g) cells were transfected with WT Flag-GNL3 or the SIM mutant and treated with DMSO or 20 μM ETO for 2 hours. Cells were assayed by co-IP using control mouse IgG or anti-Flag antibody (related to Fig. 5j). n=2 independent experiments**. (h)** Representative wide-field views of confocal images from the experiments shown in Fig. 5k. Scale bar, 10 μm.

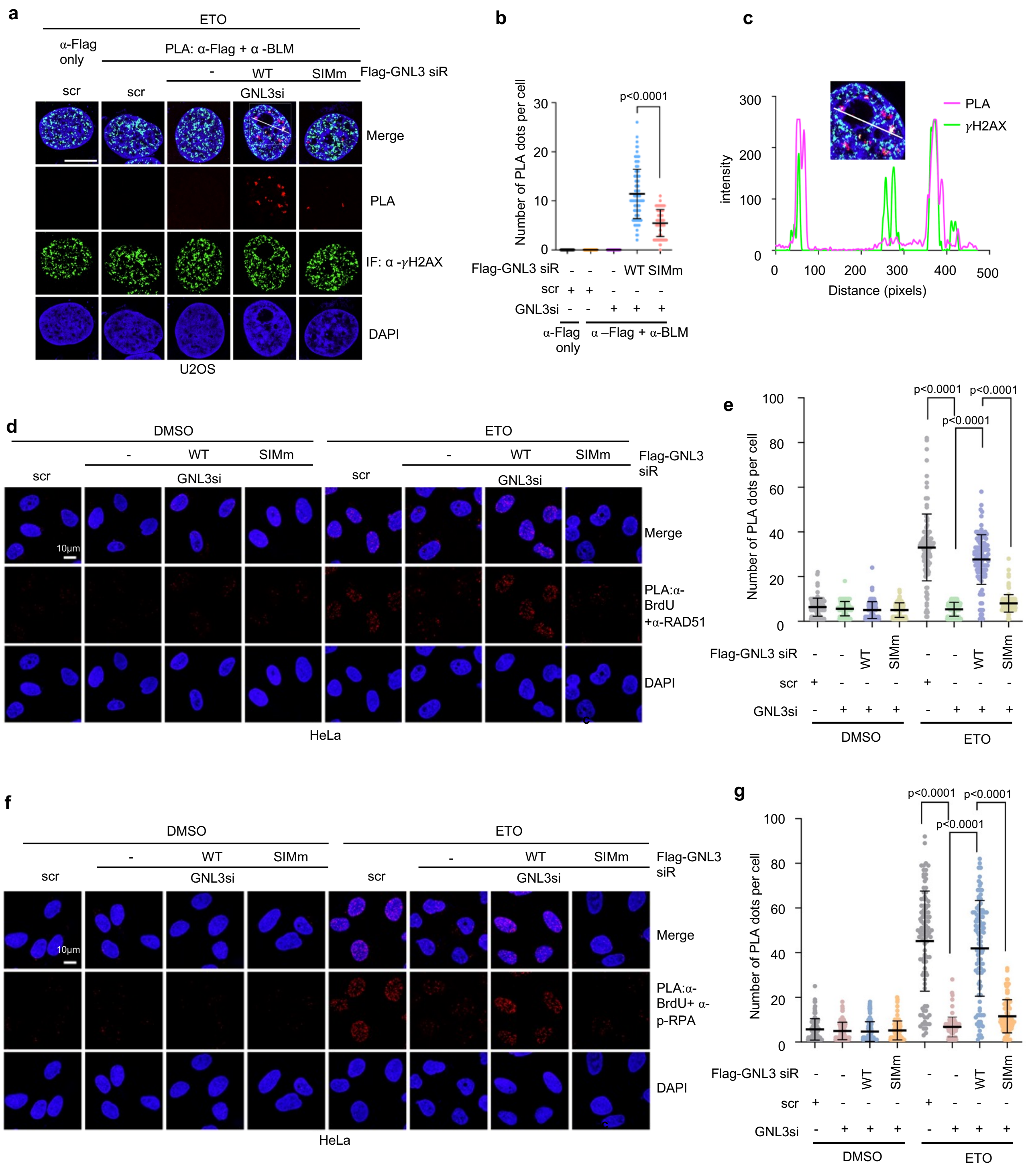
a
ETO
α-Flag only
PLA: α-Flag + α -BLM
- WT SIMm Flag-GNL3 siR
scr scr GNL3si
Merge
PLA
IF: α -γH2AX
DAPI
U2OS
b
p<0.0001
Number of PLA dots per cell
Flag-GNL3 siR - - - WT SIMm
scr + + - - -
GNL3si - - + + +
α-Flag only
α –Flag + α-BLM
c
PLA
γH2AX
intensity
Distance (pixels)
d
DMSO
ETO
- WT SIMm
Flag-GNL3 siR
scr GNL3si
10μm
Merge
PLA:α-BrdU +α-RAD51
DAPI
HeLa
e
p<0.0001
p<0.0001
p<0.0001
Number of PLA dots per cell
Flag-GNL3 siR - - WT SIMm - - WT SIMm
scr + - - - + - - -
GNL3si - + + + - + + +
DMSO
ETO
f
DMSO
ETO
- WT SIMm
Flag-GNL3 siR
scr GNL3si
10μm
Merge
PLA:α-BrdU+ α-p-RPA
DAPI
HeLa
g
p<0.0001
p<0.0001
p<0.0001
Number of PLA dots per cell
Flag-GNL3 siR - - WT SIMm - - WT SIMm
scr + - - - + - - -
GNL3si - + + + - + + +
DMSO
ETO

Supplementary Figure S6. GNL3 interacts with BLM at DNA damage sites, and this interaction is required for RAD51 and RPA loading onto ssDNA. (a)-(c) GNL3 interacts with BLM at DNA damage sites. U2OS cells transfected with scr or GNL3 siRNA, together with siRNA-resistant WT Flag-GNL3 or the SIM mutant, were treated with 20 μM ETO for 2 hours and assayed for PLA using anti-Flag and anti-BLM antibodies, followed by IF staining with anti-γH2AX antibody. Representative confocal images (a) and quantification (b) of n=100 cells per condition from three independent experiments are shown. Error bars denote mean ± SD. Significance was determined by unpaired two-tailed t-test. A fluorescence intensity plot at each pixel along a line drawn from left to right in the top fourth merged image in (a) is shown in (c) (green, γH2AX; magenta, Flag-GNL3-BLM PLA). **(d)-(g)** HeLa cells transfected with scr or GNL3 siRNA, together with siRNA-resistant WT Flag-GNL3 or the SIMm mutant, were cultured in the presence of 10 μM BrdU for 20 hours. Cells were then treated with DMSO or 20 μM ETO for 2 hours and analyzed by PLA using anti-BrdU and anti-RAD51 (d)(e) or anti-phospho-RPA2 (f)(g) antibodies. Shown are representative confocal images (d)(f) and quantification of n=110 (e) and n=100 (g) cells per condition from three independent experiments. Scale bar, 10 μm. Error bars denote mean ± SD. Significance was determined by one-way ANOVA followed by Tukey's multiple comparisons test.

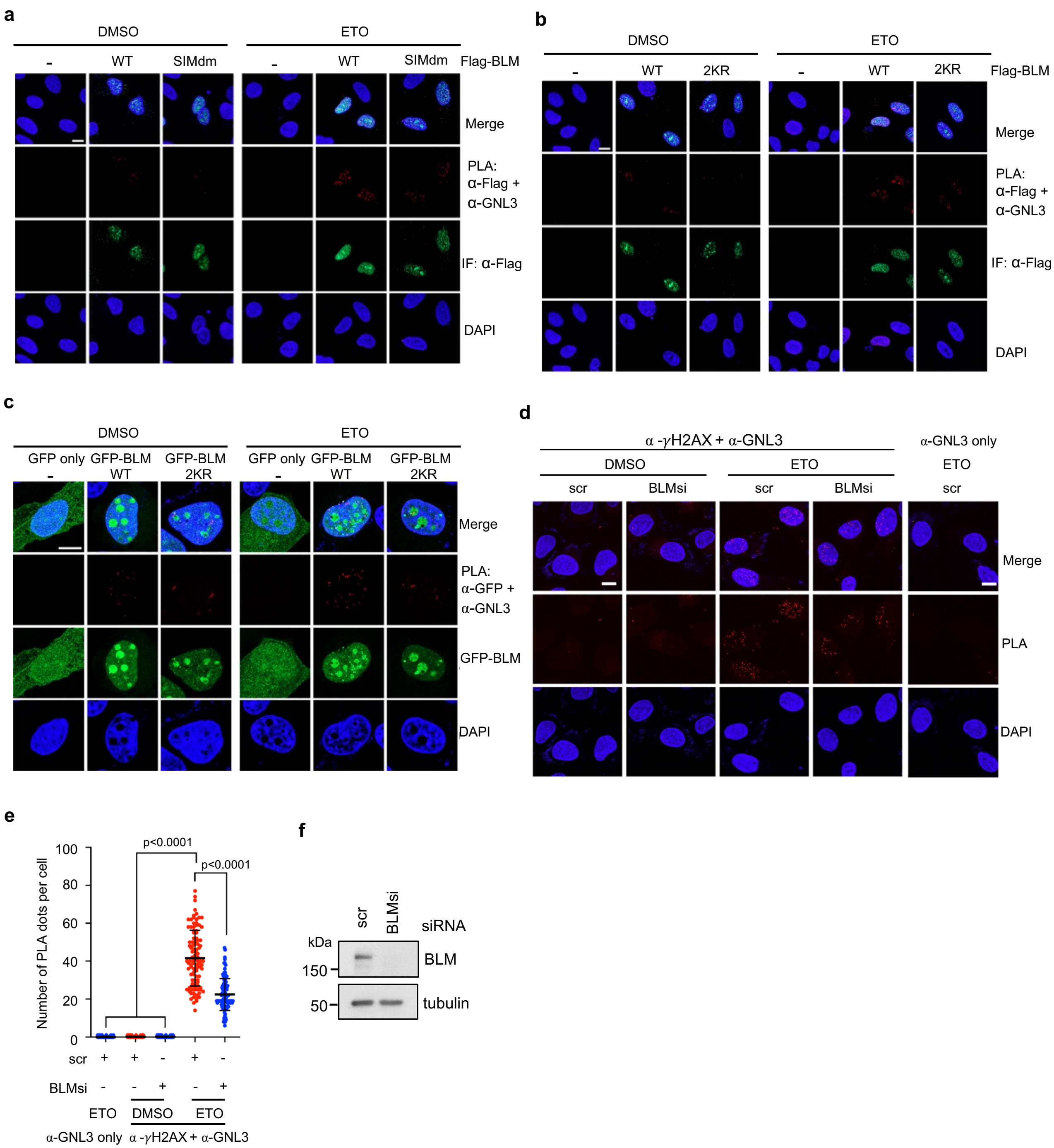
a
DMSO
ETO
-
WT
SIMdm
Flag-BLM
Merge
PLA:
α-Flag +
α-GNL3
IF: α-Flag
DAPI
b
DMSO
ETO
-
WT
2KR
Flag-BLM
Merge
PLA:
α-Flag +
α-GNL3
IF: α-Flag
DAPI
c
DMSO
ETO
GFP only -
GFP-BLM WT
GFP-BLM 2KR
Merge
PLA:
α-GFP +
α-GNL3
GFP-BLM
DAPI
d
α -γH2AX + α-GNL3
α-GNL3 only
DMSO
ETO
scr
BLMsi
Merge
PLA
DAPI
e
Number of PLA dots per cell
p<0.0001
p<0.0001
100
80
60
40
20
0
scr
BLMsi
ETO
DMSO
α-GNL3 only
α -γH2AX + α-GNL3
f
scr
BLMsi
siRNA
kDa
150
50
BLM
tubulin

Supplementary Figure S7. BLM SUMOylation and its SIM are critical for interaction with GNL3. (a) BLM SIMs are critical for its interaction with GNL3 in response to DNA damage. Representative wide-field views of confocal images from the experiments shown in Fig. 5n. Scale bar, 10 μm. **(b)** Representative wide-field views of confocal images from the experiments shown in Fig. 5q. Scale bar, 10 μm. **(c)** U2OS cells transfected with GFP, GFP-BLM or GFP-BLM^{2KR} mutant were treated with control DMSO or 20 μM ETO for 2 hours and assayed by PLA using anti-GFP and anti-GNL3 antibodies. n=2 independent experiments. **(d)-(f)** U2OS cells transfected with scr or BLM siRNA were treated with DMSO or 20 μM ETO for 2 hours and analyzed by PLA using γH2AX and anti-GNL3 antibodies. Representative confocal images (d) and quantification (e) of n=100 cells per condition from three independent experiments are shown. Error bars denote mean ± SD. Significance was determined by one-way ANOVA followed by Tukey's multiple comparison test. Cell lysates were also analyzed by IB to assess BLM knockdown efficiency (f). Scale bar, 10 μm.

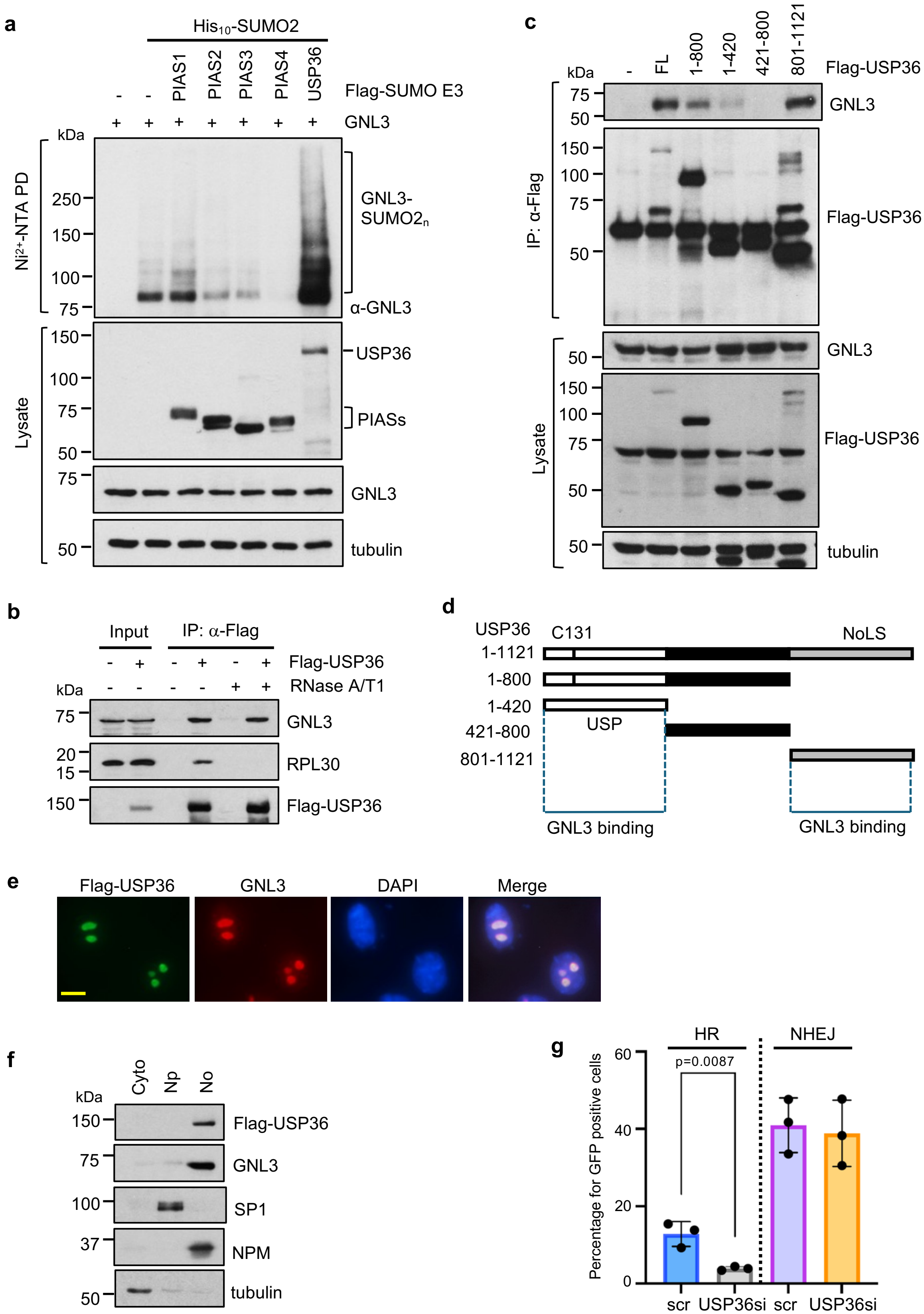
a
His10-SUMO2
Flag-SUMO E3
- - PIAS1 PIAS2 PIAS3 PIAS4 USP36
GNL3
kDa
Ni2+-NTA PD
GNL3-SUMO2n
α-GNL3
Lysate
USP36
PIASs
GNL3
tubulin
b
Input
IP: α-Flag
Flag-USP36
RNase A/T1
GNL3
RPL30
Flag-USP36
c
- FL 1-800 1-420 421-800 801-1121
Flag-USP36
IP: α-Flag
GNL3
Flag-USP36
Lysate
GNL3
Flag-USP36
tubulin
d
USP36
C131
NoLS
1-1121
1-800
1-420
USP
421-800
801-1121
GNL3 binding
GNL3 binding
e
Flag-USP36
GNL3
DAPI
Merge
f
Cyto Np No
Flag-USP36
GNL3
SP1
NPM
tubulin
g
HR
NHEJ
p=0.0087
Percentage for GFP positive cells
scr USP36si scr USP36si

Supplementary Figure S8. USP36 is a GNL3 SUMO ligase. (a) U2OS-His$_{10}$-SUMO2 cells transfected with the indicated plasmids were assayed by Ni^{2+}-NTA PD under denaturing conditions followed by IB. SUMO2 modified GNL3 is indicated. Protein expression is shown in the bottom panels. n=3 independent experiments**. (b)** HEK293 cells transfected with Flag-USP36 were assayed by co-IP using in the absence or presence of 100 μg/mL RNase A and 100U/ml RNase T1, followed by IB. n=3 independent experiments**. (c)(d)** Mapping the GNL3 binding to USP36. H1299 cells transfected with Flag-USP36 or the indicated its deletion mutants were assayed by Co-IP followed by IB (c). A diagram of USP36 deletion mutants is shown in (d). NoLS, nucleolar localization signal. n=2 independent experiments**. (e)** U2OS cells transfected with Flag-USP36 were subjected to IF staining with anti-Flag and anti-GNL3 antibodies. n=2 independent experiments**.** Scale bar, 10 μm. **(f)** HeLa cells stably expressing Flag-USP36 were fractionated into cytoplasmic (Cyto), nucleoplasmic (Np) and nucleolar fractions (No) followed by IB. Tubulin, SP1 and nucleophosmin (NPM) were used as cytoplasmic, nucleoplasmic, and nucleolar markers, respectively. n=2 independent experiments**. (g)** U2OS cells transfected with scr or USP36 siRNA, together with HR or NHEJ reporter plasmids, were assayed by flow cytometry to determine the percentage of GFP-positive cells. n=3 independent experiments**.** Significance was determined by unpaired two-tailed t-test. Error bars denote mean ± SD.

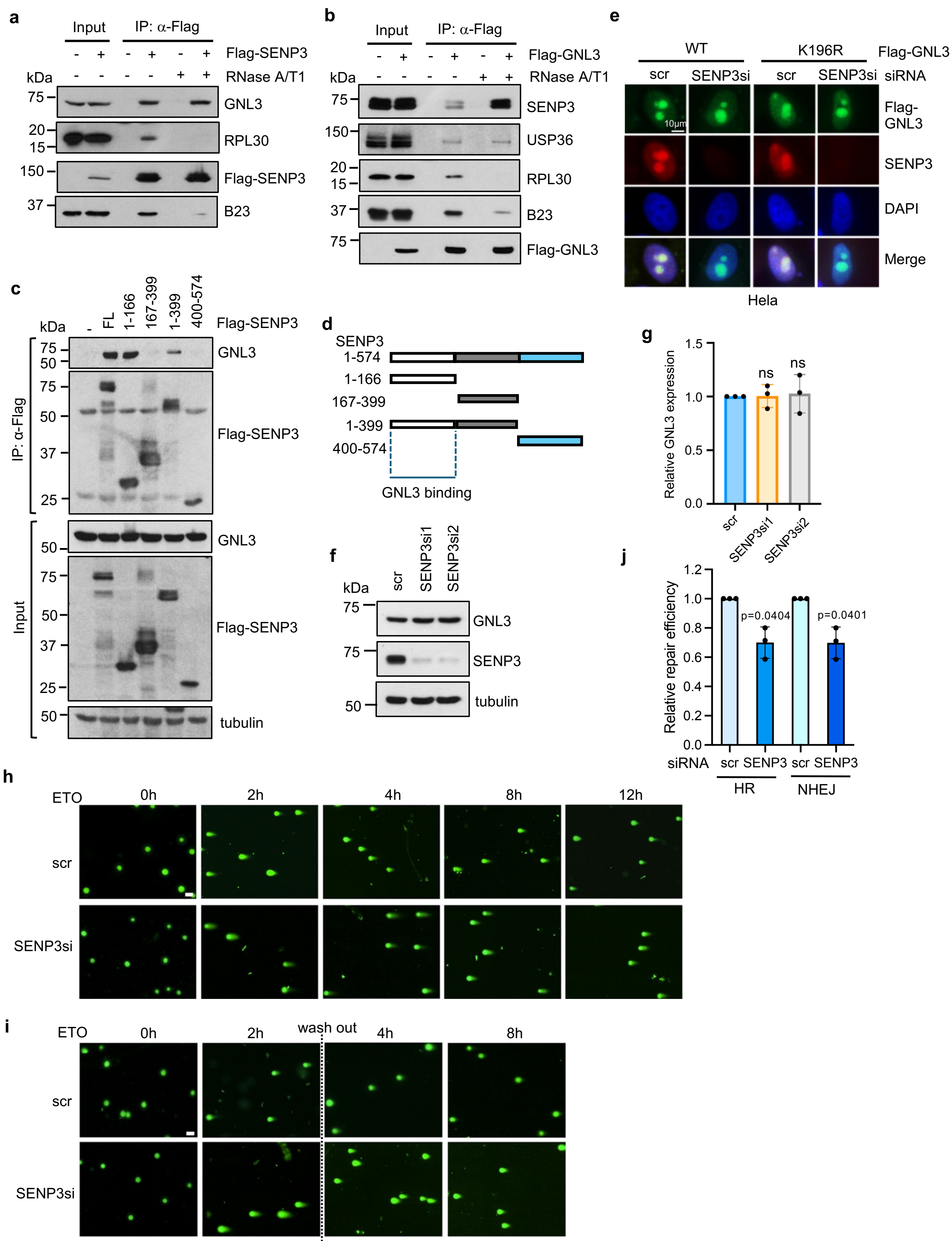
a
Input
IP: α-Flag
Flag-SENP3
kDa
RNase A/T1
75
GNL3
20
15
RPL30
150
Flag-SENP3
37
B23
b
Input
IP: α-Flag
Flag-GNL3
kDa
RNase A/T1
75
SENP3
150
USP36
20
15
RPL30
37
B23
75
Flag-GNL3
e
WT
K196R
Flag-GNL3
scr
SENP3si
scr
SENP3si
siRNA
10μm
Flag-
GNL3
SENP3
DAPI
Merge
Hela
c
kDa
-
FL
1-166
167-399
1-399
400-574
Flag-SENP3
IP: α-Flag
75
50
GNL3
75
50
37
25
Flag-SENP3
Input
50
GNL3
75
50
37
25
Flag-SENP3
50
tubulin
d
SENP3
1-574
1-166
167-399
1-399
400-574
GNL3 binding
g
Relative GNL3 expression
1.5
1.0
0.5
0.0
ns
ns
scr
SENP3si1
SENP3si2
f
kDa
scr
SENP3si1
SENP3si2
75
GNL3
75
SENP3
50
tubulin
j
Relative repair efficiency
1.2
1.0
0.8
0.6
0.4
0.2
0.0
p=0.0404
p=0.0401
siRNA
scr
SENP3
scr
SENP3
HR
NHEJ
h
ETO
0h
2h
4h
8h
12h
scr
SENP3si
i
ETO
0h
2h
wash out
4h
8h
scr
SENP3si

Supplementary Figure S9. SENP3 deSUMOylates GNL3. (a)(b) SENP3 interacts with GNL3 independently of RNA. HEK293 cells transfected with Flag-SENP3(a) or Flag-GNL3 (b) were assayed by co-IP in the absence or presence of 100 μg/mL RNase A and 100U/ml RNase T1, followed by IB. n=3 independent experiments**. (c)(d)** H1299 cells transfected with Flag-SENP3 or its deletion mutants were assayed by co-IP, followed by IB (c). n=3 independent experiments**.** A diagram of SENP3 deletion mutants is shown in (d). (**e**) U2OS cells transfected with Flag-GNL3 (WT or K196R) together with scr or SENP3 siRNA were assayed by IF staining with anti-SENP3 and anti-Flag antibodies. n=2 independent experiments**. (f)(g)** U2OS cells transfected with scr or SENP3 siRNAs were assayed by IB. Shown are the representative IB blots (f) and quantification from three independent experiments (g). Error bars denote mean ± SD.
Ns, no significance. (**h**) Representative wide-field views of Comet assay images from the experiments shown in Fig. 7i. Scale bar, 10 μm. (**i**) Representative wide-field views of Comet assay images from the experiments shown in Fig. 7k. Scale bar, 10 μm. (**j**) U2OS cells transfected with scr or SENP3 siRNA, together with HR or NHEJ reporter plasmids, were assayed by flow cytometry for the percentage of GFP-positive cells. n=3 independent experiments**.** Significance was determined by unpaired two-tailed t-test. Error bars denote mean ± SD.

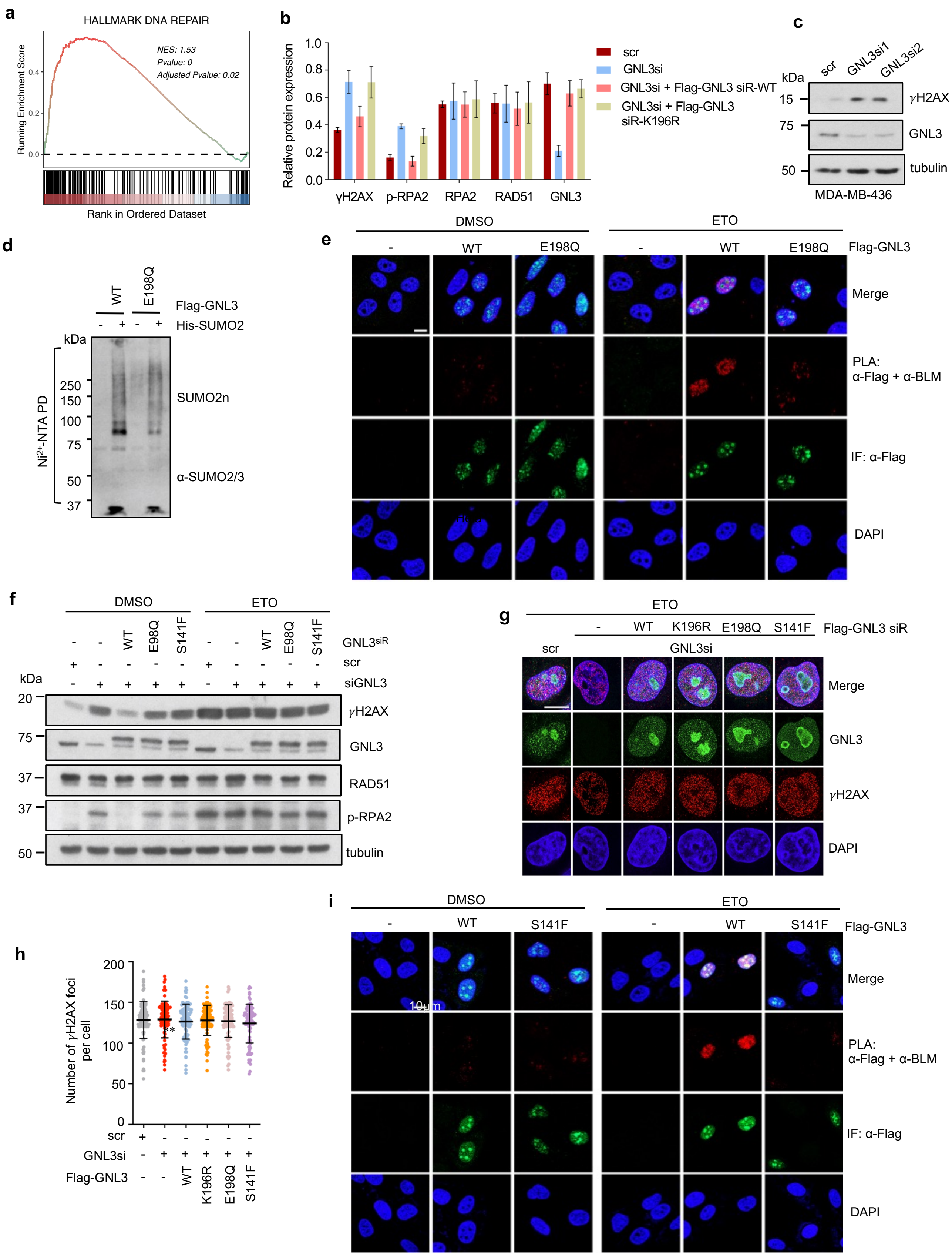
a
HALLMARK DNA REPAIR
NES: 1.53
Pvalue: 0
Adjusted Pvalue: 0.02
Running Enrichment Score
Rank in Ordered Dataset
b
Relative protein expression
γH2AX
p-RPA2
RPA2
RAD51
GNL3
scr
GNL3si
GNL3si + Flag-GNL3 siR-WT
GNL3si + Flag-GNL3 siR-K196R
c
kDa
scr
GNL3si1
GNL3si2
γH2AX
GNL3
tubulin
MDA-MB-436
d
WT
E198Q
Flag-GNL3
His-SUMO2
kDa
Ni2+-NTA PD
SUMO2n
α-SUMO2/3
e
DMSO
ETO
WT
E198Q
Flag-GNL3
Merge
PLA: α-Flag + α-BLM
IF: α-Flag
DAPI
f
DMSO
ETO
WT
E98Q
S141F
GNL3siR
scr
siGNL3
kDa
γH2AX
GNL3
RAD51
p-RPA2
tubulin
g
ETO
WT
K196R
E198Q
S141F
Flag-GNL3 siR
scr
GNL3si
Merge
GNL3
γH2AX
DAPI
h
Number of γH2AX foci per cell
scr
GNL3si
Flag-GNL3
WT
K196R
E198Q
S141F
i
DMSO
ETO
WT
S141F
Flag-GNL3
10μm
Merge
PLA: α-Flag + α-BLM
IF: α-Flag
DAPI

Supplementary Figure 10. GNL3 is implicated in breast cancer. (a) GNL3 expression is associated with the DNA damage hallmark in TNBC. Shown is the GSEA score. **(b)** Quantification of protein expression in Fig. 8i from two biological replicates. **(c)** MDA-MB-436 cells transfected with scr or GNL3 siRNAs were analyzed by IB. n=2 independent experiments**. (d)** IB with anti-SUMO2/3 antibody to detect total His-SUMO2 expression in the *in vivo* SUMOylation assay shown in Fig. 9b. n=3 independent experiments**. (e)** Representative wide-field views of confocal images from the experiments shown in Fig. 9c. **(f)** HeLa cells transfected with scr or GNL3 siRNA, together with siRNA-resistant WT Flag-GNL3 or the E198Q and S141F mutants, were treated with control DMSO or 20 μM ETO for 2 hours and analyzed by IB. n=2 independent experiments**. (g)(h)** HeLa cells transfected with scr or GNL3 siRNA, together with siRNA-resistant Flag-GNL3 (WT, K196R, E198Q, or S141F), were treated with 20 μM ETO for 2 hours and assayed by IF staining with anti-GNL3 and anti-γH2AX antibodies. Representative confocal images (g) and the quantification (h) of the γH2AX foci of n=100 cells per condition from three independent experiments are shown. Error bars denote mean ± SD. No significance difference between different groups was determined by one-way ANOVA followed by Tukey's multiple comparison test. (related to Fig. 9e). **(i)** Representative wide-field views of confocal images from the experiments shown in Fig. 9g. Scale bars, 10 μm.

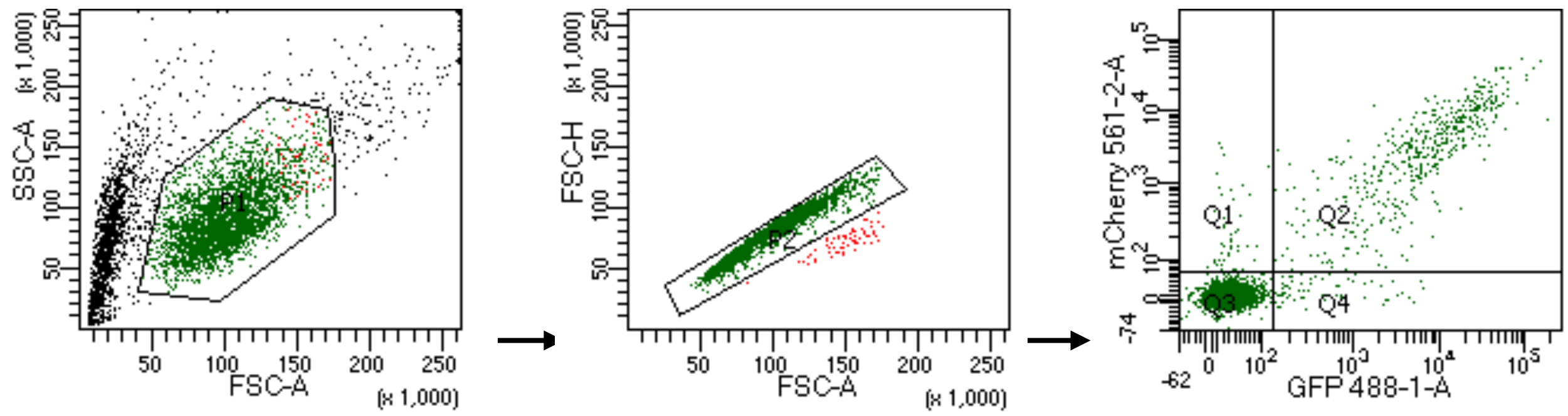

Supplementary Figure 11. FACS gating strategy for HR and NHEJ assays. Repair efficiency was calculated as Q2/(Q1+Q2).
