# Supplementary material for: GNL3 SUMOylation is essential for DNA double-strand break repair by homologous recombination
Source: Nat Commun. 2026 Aug 24;17:10122. doi: 10.1038/s41467-026-77013-1 (PMC13598120; doi:10.1038/s41467-026-77013-1)
Supplement: Supplementary file 2 [file 41467_2026_77013_MOESM2_ESM.pdf]

nature portfolio

Peer Review File

GNL3 SUMOylation is essential for DNA double-strand break repair by homologous recombination

Corresponding Author: Dr Mushui Dai

This file contains all reviewer reports in order by version, followed by all author rebuttals in order by version.

Version 0:

Reviewer comments:

Reviewer #1

(Remarks to the Author)
This manuscript presents a comprehensive and mechanistically detailed study on the role of GNL3 SUMOylation in homologous recombination (HR)-mediated DNA double-strand break (DSB) repair. The authors provide compelling evidence that GNL3 is SUMOylated at K196 in response to DNA damage, and this modification is essential for its interaction with the BLM-DNA2 resection complex, thereby promoting DNA end resection, RPA/RAD51 loading, and HR repair. The study also identifies USP36 as a SUMO ligase and SENP3 as a deSUMOylase for GNL3, and links GNL3 SUMOylation defects to breast cancer pathogenesis and therapy resistance. However, several issues need to be addressed before further consideration.

1 The data of sumo blot is not convincing. The sumo blot is cut off at 75kDa for the most experiments and it didn't appears the smear goes down to a much lower size, raising questions into what is actually being visualized since the IP of LATS1 seems to run at near 60kDa. Furthermore, whether the sumo experiments IP with His beads? Please add His expression in IP sample.

2 "Irradiation (IR)" is a classical method for inducing DNA damage. In Fig1f, cells treated with ETO, CPT and HU and checked GNL3 SUMOylation. Please test whether IR exposure promotes SUMO modification of GNL3.

3 Please provide for mass spectrometry was performed to analyze GNL3 SUMOylation sites, along with making this data accessible to the public.

4 In Fig 3a, PLA assays observed that treatment of cells with either ETO or X-rays markedly induced the interaction between GNL3 and gH2AX. But the lack of co-localization between GNL3 foci and γH2AX foci in Figure 2C raises the question of whether GNL3 functions directly at DNA damage sites.

5 The images in Fig2h,7i,7k are not convincing. It would be helpful to include a more zoomed out images that include many cells (ideally with cells of similar confluence).

6 γH2AX is an early "flag" or "recruitment platform," while RAD51 is a late "executor" of the repair. They are functionally linked through this crucial amplification and recruitment pathway. Fig 3 demonstrates that GNL3 interacts with both γH2AX and RAD51 upon DNA damage. What is the precise mechanism governing these interactions?

7 Knockdown of GNL3 markedly induced DDR as shown by the increased levels and foci formation of phosphorylated H2AX (gH2AX) in U2OS cells in Figs. 2c-2e, but knockdown of GNL3 does not abolish gH2AX foci formation in cells treated with ETO in supplementary Fig. 2a & 2b. Why?

8 Knockdown of GNL3 also abolishes RAD51 or RPA2 foci formation induced by ETO treatment (Fig. 3f,3g,3h,3i). Why knockdown of GNL3 increased the foci formation of BRCA1 (Fig. 3j, 3k), BARD1 (Supplementary Fig. 3a,3b), 53BP1 (Supplementary Fig. 3c, 3d) and MDC1 (Supplementary Fig. 3c , 3e) following the ETO treatment?

9 This manuscript detailed study on the role of GNL3 SUMOylation in homologous recombination (HR)-mediated DNA double-strand break (DSB) repair, and this modification is essential for its interaction with the BLM-DNA2 resection complex.

The authors did not examine homologous recombination (HR) assay or DNA resection using AsiSI system.

10 In general, conclusions from the western blot experiments should be quantified over several replicates.

Reviewer #2

(Remarks to the Author)
In this study, Yunhan et al. report that GNL3 is involved in DNA repair through its SUMOylation. Using the GPS-SUMO tool, the authors predicted K196 and K275 as potential SUMOylation sites and further validated that GNL3 is SUMOylated at K196 but not at K275. Depletion of GNL3 induced endogenous H2AX signaling; however, the SUMO-deficient mutant GNL3-K196R failed to rescue H2AX accumulation upon endogenous GNL3 knockdown, highlighting the importance of GNL3 SUMOylation in suppressing endogenous DNA damage. The authors subsequently identified USP36 as the SUMO E3 ligase for GNL3 and SENP3 as the enzyme responsible for its de-SUMOylation. Mechanistically, the GNL3 SUMO-mutant displayed reduced DNA end resection. IP-mass spectrometry further identified BLM as a GNL3-interacting protein. PLA assays using a GNL3 SIM-mutant (SUMO-Interacting Motif) demonstrated that the SUMO–SIM interaction between GNL3 and BLM is critical for BLM-mediated DNA end resection upon damage. Overall, this study reveals a crucial role for GNL3 SUMOylation in the DNA damage response through its ability to interact with BLM and other DNA damage factors involved in homologous recombination. However, there are several weaknesses in the study that need to be addressed. The manuscript has an overreliance on PLA for demonstrating damage associated phenotypes, lacks direct evidence that GNL3 promotes HR repair and its many interactions are not properly controlled, which could be explained by indirect interactions identified by IP analysis. To better support the proposed mechanism, the role of GNL3 in homologous recombination needs further validation to strengthen the authors' conclusions.

Major comments
1. GNL3 is known to involved in replication stress and telomere regulation through TRF1 (PMID: 3796589, 22045740, 22641345), and in the manuscript the authors state that SUMO-SIM interaction of BLM. It is known that SUMO BLM is involved in ALT telomere maintenance and stalled replication responses (PMID:39038850, 24027577). The majority of the figures were done exclusively in U2OS cells, which is an ATL+ cell line. The authors need to rule out that the results obtained are not solely due to affects on ALT. Key data needs to be done in a panel of cell lines, including non-ALT cancer cells.
2. The authors should provide more conclusive data indicating GNL3 is involved in DNA end resection at DNA damage sites but not involved in replication stress related ALT telomere maintenance. The authors need to have orthogonal approaches to show GNL3 is recruited to DNA damage sites other than PLA assay, such as Laser DNA damage, ChIP at a DSB, chromatin fraction, etc. Most of the experiment were done by overexpression. Some key experiments need to be performed with proteins expressed at their endogenous levels.
3. In Fig. 2, the whole figure used three different cell lines without any rational and proper explanation. It needs to provide a justification for using these cell lines and key experiments should be done in multiple different cell lines, to rule out genetic and cell line specific effects.
4. Fig. 3c, IP results showed K196R had less interaction with H2AX and Rad51. It is possible that IP is pulling down everything and K196R mutant is less chromatin bound. The authors should provide a negative control for IP and should use chromatin fractionation to compare chromatin binding ability of GNL3 WT and K196R mutant.
5. Fig. 3f, Rad51 expression level should be provided in GNL3 knock down. The concentration of Etoposide might need to be less to have reduced RAD51 foci, making quantification and results easier to interpret.
6. U2OS is a known ALT+ cell line, and GNL3 is known to interact with TRF1. In Fig. 2 J and K, the MTT assay and clonogenic assays using U2OS would be hard to pinpoint whether telomere integrity is involved. Pherphaps using telomerase+ cell line such us HeLa could address this uncertainty.
7. GNL3 is involved in replication stress (PMID: 3796589), so this needs to be addressed to know if H2AX signals in GNL3 depletion is due to replication stress, DNA damage or a mixture of both. If GNL3 is involved in DNA repair, HR and NHEJ reporter assays need to be done to show this functionally. Depletion of GNL3 might have defect/ delay repair upon drug treatment. Although H2AX was no affect upon Etoposide treatment in GNL3 depleted cells (Fig. S2a), but it might show defect of repair in later time points compared to siControl. On the other hand, overexpressing GNL3 might repair faster.
8. Fig. 3c, GNL3 SUMO-mutant showed less binding to H2AX. Dose GNL3 have a phospho binding site? The authors should address why less binding of H2AX would affect DNA end resection and DNA repair.
9. Fig. 3J and Fig. S3, GNL3 is proposed to be involved in resection but no effects on BRCA1/53BP1/BARD1/MDC1 loading but only RAD51 and RPA2. If these cells have less end resection as proposed in the manuscript, it would be expected to have increased 53BP1 foci accumulation at damage sites. The authors should address why GNL3 is involved in DNA end resection but does not affect BRCA1/53BP1/BARD1/MDC1 foci formation.
10. Fig. 4, Dose GNL3 affect other resection portions at DNA damage sites such as MRE11, CtIP, Exo1?
11. Fig 4a, Does GNL3 IP-MS data also show known GNL3 interaction factors?
12. Fig. 5, Similar to SUMO K196R mutant, SIM mutant might just have less chromatin binding. Authors should provide negative control for IP and should use chromatin fractionation to check chromatin binding ability of GNL3 WT and SIM mutant.
13. SUMOylated BLM has effect on PML body localization (PMID:15829507). GNL3 SIM- mutant is shown to have less interaction with BLM by IP-WB and PLA assay. The claim of GNL3 involved in DNA end resection by SUMO-SIM interaction with BLM/DNA2 should have additional evidence. More evidence showing that GNL3 SUMO-SIM interaction with BLM is involved in DNA end resection should be provided. Dose GNL3 SIM-mutant also effects RPA and Rad51 loading on ssDNA?
14. Fig. 6K, Depletion of USP36 showed lower level of GNL3, and this is thought to be through post-translational modification. Dose overexpressing UPS36 have higher GNL3 level?

15. Fig. 8h, depletion of GNL3 confers drug sensitivity in MDA-MB-231 cells, the authors should include E198Q and S141F and WT constructs for survival assays. Fig. 8, the GNL3 mutation variants E198Q and S141F in U2OS cells, the authors should address if this is due to less resection or is it because of PML recruitment, ie telomere maintenance.
16. GNL3 depletion in MDA-MB-231 cells is shown to lead to PARPi sensitivity but not in BRCA-deficient breast cancer cells. This is hard to reconcile if GNL3 is involved in HR since these cells are hypomorph so depletion of GNL3 would likely lead to a more severe defect in HR and increase PARPi sensitivity. Can the authors explain this? Also, BRCA-deficient cells should be very sensitive to PARPi compared to BRCA-proficient cells, which is not clear from the data in Figure 8 I and K.
17. The studies with USP36 and SENP3 are underdeveloped. How does a DUB also function at a SUMO E3 ligase? Is its DUB activity involved? How do these proteins impact HR repair and endogenous DNA damage (i.e. replication stress, telomeres, DSB repair)?

Minor comments
1. In the introduction, “Yet, the majority of cancers are HR-proficient and PARPi resistant”. PARPi is less sensitive in BRCA-proficient cells compared to BRCAness, rather than PARPi resistant.
2. The formatting should be more consistent. It is a bit distracting throughout the manuscript to have non aligned panels and text. The text should be aligned, and labels should be more consistent.

Reviewer #3

(Remarks to the Author)
This manuscript by Yang and colleagues investigates how the nucleolar GTP-binding protein GNL3 regulates homologous recombination (HR) repair through a SUMOylation-dependent mechanism. The authors show that GNL3 is SUMOylated at K196, which can be enhanced by DNA damage, and that it is required for RPA/RAD51 loading, for GNL interaction with the BLM–DNA2 complex. The authors further demonstrate that the interaction between GNL3 and BLM is dependent on a SUMO–SIM interaction, supported by the mutations in SIM within GNL3. In addition, they identify USP36 as the SUMO ligase for GNL3 and SENP3 as its deSUMOylase. Therapeutically, GNL3 levels are significantly elevated in breast cancers, and GNL3 depletion or SUMO-defective mutants impair HR repair and sensitize breast cancer cells to ETO treatment. Overall, the study proposes that GNL3 SUMOylation is a critical regulatory node that controls HR competency in cancer cells. While GNL3 SUMOylation is well-supported with data, how much of this SUMOylation contributes to DNA repair is questionable, particularly given that GNL3 and its SUMOylation-defective mutations have no effect on DNA repair and gH2AX foci formation with ETO treatment (supplementary Fig. 2a, b, c, d). In addition, the SUMO ligase USP36 and SENP3 all co-localize with GNL3 in the nucleolus, suggesting their main mode of action might be in the nucleolus. The USP36 and SENP3 manipulation results might be the regulation of proteins other than GNL3. The IP data regarding all interactions between GNL3 and the DDR proteins are not localization-specific, and the co-localization data are based on PLA without evidence that these PLA foci are at DNA damage sites. These may reflect changes in global SUMOylation dynamics in response to nucleolar stress induced by manipulation of GNL3. I’d be more convinced if there were a clear GNL3 co-localization with BLM/DNA at DNA damage sites, and their colocalization at DNA damage sites depends on SUMO/SIM of each protein. Other comments are:
1. Are BLM and the BLM–DNA2 complex required for GNL3 recruitment to damage foci and vice versa? Knockdown of BLM would help determine whether GNL3 functions upstream or downstream of BLM–DNA2, and whether its role in promoting DNA end resection is fully or only partially dependent on this complex. The authors also propose that GNL3 binds ssDNA generated by MRN-CtIP-mediated short-range resection. Is there additional evidence supporting GNL3 acting at the short-range initiation step, rather than functioning exclusively during long-range BLM-DNA2-mediated resection?
2. The author has identified USP36 as the SUMO ligase for GNL3. The current data rely primarily on overexpression and knockdown experiments, in which do not distinguish between direct enzymatic SUMOylation by USP36 or indirect effects. To confirm USP36 as a SUMO ligase for GNL3, an in vitro enzymatic assay using purified proteins should be done.
3. The rationale for investigating GNL3 in the context of SUMOylation and HR repair is not clearly described. More background on prior work on GNL3’s role in ribosome biogenesis, p53, and HR, particularly how these roles are differentiated/linked, and what is already known for its role in HR, would be helpful.
4. In Fig. 2C, the DAPI staining shows irregular cell morphology. It would be helpful to provide higher-quality images.
5. In Figure 6h, the WB labels do not correspond correctly to the positions of each band.
6. The SUMO E1 inhibitor is listed incorrectly as ML972 in the Methods. In the HR and NHEJ reporter assays Methods, ‘transection’ should be ‘transfection’.
7. Some of the sentences are long and confusing.

Reviewer #4

(Remarks to the Author)
I co-reviewed this manuscript with one of the reviewers who provided the listed reports. This is part of the Nature Communications initiative to facilitate training in peer review and to provide appropriate recognition for Early Career Researchers who co-review manuscripts.

Version 1:

Reviewer comments:

Reviewer #1

(Remarks to the Author)
The authors have carefully responded to and addressed all the reviewers' comments, and the quality of the manuscript has significantly improved. I have no further substantive comments.

Reviewer #2

(Remarks to the Author)
The authors have done extensive revisions that further support a role for GLN3 and its SUMOylation in DNA end resection and DNA repair. The authors have now addressed all of this reviewers concerns prior to publication.

Reviewer #3

(Remarks to the Author)
The authors have addressed all of my concerns.

Reviewer #4

(Remarks to the Author)
I co-reviewed this manuscript with one of the reviewers who provided the listed reports. This is part of the Nature Communications initiative to facilitate training in peer review and to provide appropriate recognition for Early Career Researchers who co-review manuscripts.

Open Access This Peer Review File is licensed under a Creative Commons Attribution 4.0 International License, which permits use, sharing, adaptation, distribution and reproduction in any medium or format, as long as you give appropriate credit to the original author(s) and the source, provide a link to the Creative Commons license, and indicate if changes were made.
In cases where reviewers are anonymous, credit should be given to 'Anonymous Referee' and the source.
The images or other third party material in this Peer Review File are included in the article's Creative Commons license, unless indicated otherwise in a credit line to the material. If material is not included in the article's Creative Commons license and your intended use is not permitted by statutory regulation or exceeds the permitted use, you will need to obtain permission directly from the copyright holder.
To view a copy of this license, visit https://creativecommons.org/licenses/by/4.0/

Responses to reviewers' comments:

We appreciate all the reviewers' constructive comments and suggestions that have allowed us to improve our manuscript. In response to these comments, we have performed an extensive series of new experiments and further demonstrated a critical role for GNL3 SUMOylation in HR repair. We added a total of **71** new panels of data (New Figures 1c, 1e, 1i; 2k-o; 3c-3f, 3h, 3o, 3p; 4e, 4f, 4n, 4o; 5f-5i; 6b; 8r, 8s, 8w and New Supplementary Figures S1c-S1e; S2a, S2d, S2e, S2i, S2j; S3a-S3h; S4g-S4l; S5a, S5c, S5f, S5g, S5i; S6a-S6g; S7d-S7f; S8g; S10b, S10d, S10f-S10h; S11) into our revised manuscript and modified **9** new panels (New Figures 1a, 1b, 1d, 1f-1h; 2b, 2f and new supplementary Figure S2g). We also modified text correspondingly. Our responses to the reviewers' comments are described as below.

<u>Response to Reviewer #1:</u>

1. The data of sumo blot is not convincing. The sumo blot is cut off at 75kDa for the most experiments and it didn't appear the smear goes down to a much lower size, raising questions into what is actually being visualized since the IP of LATS1 seems to run at near 60kDa. Furthermore, whether the sumo experiments IP with His beads? Please add His expression in IP sample.

Our response: We thank the reviewer for this concern. Yes, we used Ni^{2+}-NTA agarose beads (His beads) to pull down GNL3 conjugated with His-tagged SUMO2. We have now added full blots for total SUMOylation (His-SUMO2 expression) in major SUMOylation assays (New modified Figs. 1a, 1b,1d,1f-1g; 2b & new supplementary Figs. S1d, S10d) The molecular weights for SUMO-modified GNL3 are higher than that of unmodified GNL3 (~60 kDa). The uncropped images with areas below unmodified GNL3 beyond the cutoff for all Western blots are provided in the <u>Source Data</u>.

2. "Irradiation (IR)" is a classical method for inducing DNA damage. In Fig1f, cells treated with ETO, CPT and HU and checked GNL3 SUMOylation. Please test whether IR exposure promotes SUMO modification of GNL3.

Our response: Thanks for the important suggestion. We have now performed additional experiments and showed that IR exposure also promotes SUMOylation of GNL3 (New supplementary Fig. S1d, S1e).

3. Please provide for mass spectrometry was performed to analyze GNL3 SUMOylation sites, along with making this data accessible to the public.

Our response: Our mass spectrometry data for GNL3-associated proteins does not contain information on GNL3 SUMOylation sites, as there is no Lys/Arg near C-terminal di-Gly that would permit trypsin digestion-based detection of SUMOylated peptides/remnants (unlike in the case of ubiquitin). However, several published datasets are available showing that K196 is a GNL3 SUMO site (please see PMID 29942033; 24782567; 34518535)

4. In Fig 3a, PLA assays observed that treatment of cells with either ETO or X-rays markedly induced the interaction between GNL3 and gH2AX. But the lack of co-localization between GNL3 foci and γH2AX foci in Figure 2C raises the question of whether GNL3 functions directly at DNA damage sites.

Our response: We thank the reviewer for this critical comment. Although GNL3 is mainly localized to the nucleolus, it is a nucleolar-nucleoplasm shuttling protein regulated by GTP-binding [1, 2]. Using high-resolution immunofluorescence imaging, we showed that nucleoplasmic GNL3 largely co-localizes with γH2AX following ETO treatment (New Supplementary Figs S3c, S3d). Similar data was also reported by the Tsai group[3] in cells treated with HU. In this revised manuscript, we provided additional evidence supporting that GNL3 associates with DNA damage sites as described below:

(1). Using a DNA damage in situ proximity ligation assay (DI-PLA) assay, wherein DSBs are blunt-ended and ligated with a biotinylated DNA probe, followed by PLA using anti-biotin and anti-GNL3

antibodies, we showed a robust increase in the interaction of GNL3 with ETO-induced DSBs (New Figs. 3c, 3d).

(2). We performed chromatin immunoprecipitation (ChIP) assays by taking advantage of ER-AsiSI U2OS cells, in which DSBs can be induced by the addition of 4-OHT (New Supplementary Fig. S3a). We showed that GNL3 binding to an AsiSI digestion site on chromosome 1 was specifically detected only when DSBs are generated by 4-OHT-induced AsiSI activation (New Fig. 3e, 3f), demonstrating that GNL3 associates with DSBs generated by site-specific cleavage.

Together, these results strongly demonstrate that GNL3 directly binds to DNA damage sites.

5. The images in Fig2h,7i,7k are not convincing. It would be helpful to include a more zoomed out images that include many cells (ideally with cells of similar confluence).

Our response: Thanks, we have now included more zoomed-out images showing more cells in the modified Fig. 2f & Supplementary Fig. S2g; New Supplementary S9h, S9i.

6. γH2AX is an early "flag" or "recruitment platform," while RAD51 is a late "executor" of the repair. They are functionally linked through this crucial amplification and recruitment pathway. Fig 3 demonstrates that GNL3 interacts with both γH2AX and RAD51 upon DNA damage. What is the precise mechanism governing these interactions?

Our response: This is an excellent point. We observed the GNL3-γH2AX interaction in cells in response to DSBs as evidenced by both PLA and co-IP, suggesting that GNL3 recruitment to the sites of DSBs may be an early event in DDR. γH2AX is also maintained during late stages of repair process and our data suggest that GNL3 plays a role in BLM-DNA2 mediated DNA end resection. This could also explain GNL3 interaction with γH2AX. The GNL3-γH2AX interaction could be indirect via other DDR proteins during a concerted recruitment of DDR proteins. The GNL3-RAD51 interaction suggests that GNL3 may have additional roles in RAD51 loading, as in the case of BLM-RAD51 interaction[4]. This may also involve SUMO-SIM interactions, given that RAD51 can be SUMOylated and contains SIM[5, 6]. This is certainly an important point that we hope, the reviewer can allow us to address in a future study.

7. Knockdown of GNL3 markedly induced DDR as shown by the increased levels and foci formation of phosphorylated H2AX (gH2AX) in U2OS cells in Figs. 2c-2e, but knockdown of GNL3 does not abolish gH2AX foci formation in cells treated with ETO in supplementary Fig. 2a & 2b. Why?

Our response: GNL3 plays a role in DNA end resection required for HR repair. Thus, knockdown of GNL3 induces replication stress-mediated DNA damage and thus γH2AX. Given the critical role for GNL3 in HR repair, ETO treatment-induced DNA damage and thus γH2AX are unable to be resolved by GNL3 knockdown. Further supporting the role for GNL3 in HR repair, time course studies of cells with ETO washout showed that GNL3 knockdown impairs the resolution of γH2AX foci following ETO removal (New supplementary Fig. S2d & S2e).

8. Knockdown of GNL3 also abolishes RAD51 or RPA2 foci formation induced by ETO treatment (Fig. 3f,3g,3h,3i). Why knockdown of GNL3 increased the foci formation of BRCA1 (Fig. 3j, 3k), BARD1 (Supplementary Fig. 3a,3b), 53BP1 (Supplementary Fig. 3c, 3d) and MDC1 (Supplementary Fig. 3c, 3e) following the ETO treatment?

Our response: Knockdown of GNL3 prevents the repair of replication stress-induced DNA damage, leading to DNA damage as indicated by induction of γH2AX, which facilitate the concerted recruitment of DDR proteins MDC1, BRCA1-BARD1 and 53BP1, upstream of RAD51 loading (with ETO treatment as a positive control). GNL3 plays a critical role in DNA end resection. Therefore, GNL3 knockdown abolishes RAD51 and RPA2 foci upon ETO treatment. This is also supported by Tsai group showing that knockdown of GNL3 induces BRCA1 foci, but not RAD51[3].

9. This manuscript detailed study on the role of GNL3 SUMOylation in homologous recombination (HR)-mediated DNA double-strand break (DSB) repair, and this modification is

essential for its interaction with the BLM-DNA2 resection complex. The authors did not examine homologous recombination (HR) assay or DNA resection using AsiSI system.

Our response: We thank the reviewer for these great points. We have now performed the HR reporter and DNA resection assays. Using ER-AsiSI U2OS cell system, we showed that knockdown of GNL3 markedly reduced the relative levels of ssDNA at an AsiSI-induced DSB site, similar to BRCA1 knockdown (New Figs 4n, 4o). Also, reporter assays using DR-GFP reveal that knockdown of GNL3 markedly reduced HR repair efficiency (New supplementary Fig. S2j). Together, these results further support the critical role for GNL3 in DNA end resection during homologous recombination.

10 In general, conclusions from the western blot experiments should be quantified over several replicates.

Our response: Thanks, we have now included quantitative analyses from three independent experiments for key western blot data (New Figs 1c, 1e, 1i, 3h and New Supplementary Fig. S1c, S5a,10b).

Response to Reviewer #2:

We appreciate the reviewer's overall support of our finding as well as the general concerns about "**lacking direct evidence that GNL3 promotes HR repair**", "**many interactions are not properly controlled**" and "**the role of GNL3 in homologous recombination needs further validation**". These general concerns are addressed below in our responses to comments #2, #7 and #12, respectively.

Major comments

1. GNL3 is known to involved in replication stress and telomere regulation through TRF1 (PMID: 3796589, 22045740, 22641345), and in the manuscript the authors state that SUMO-SIM interaction of BLM. It is known that SUMO BLM is involved in ALT telomere maintenance and stalled replication responses (PMID:39038850, 24027577). The majority of the figures were done exclusively in U2OS cells, which is an ATL+ cell line. The authors need to rule out that the results obtained are not solely due to affect on ALT. Key data needs to be done in a panel of cell lines, including non-ALT cancer cells.

Our response: We really thank the reviewer for this critical comment regarding the potential sole role of ALT-associated mechanisms. In our original manuscript, we showed some data in ALT-negative cell lines including HeLa, MDA-MB-231 and MDA-MB-436 cells, including ETO treatment-increased GNL3 SUMOylation in HeLa cells (Fig. 1d) and GNL3 knockdown-induced γH2AX levels and foci (now Fig. 8g & 8h) in MDA-MB-231 cells and γH2AX levels in MDA-MB-436 cells (now supplementary Fig. 10c). To fully address this concern, we have performed additional extensive experiments in HeLa cells, MDA-MB-231 cells, which are all ALT-negative, summarized below.

(1) Knockdown of GNL3 led to a marked increase in the levels of γH2AX (New Figs 2k, 2l) and phospho-RPA2 (New supplementary Fig. S2i), which can be rescued by wild-type GNL3, but not the SUMO-deficient K196R mutant (New Fig. 2l; supplementary Fig. S2i) in HeLa cells.

(2) Knockdown of GNL3 abolished ETO-induced RAD51 foci formation, which can be rescued by WT GNL3, but not the K196R mutant (New supplementary Fig. S3g, S3h) in HeLa cells.

(3) GNL3 knockdown suppresses HeLa cell growth, which can be rescued by WT GNL3, but not the K196R mutant, as determined by cell viability (New Fig. 2m) and colony formation (New Figs. 2n, 2o) assays.

(4) Co-immunoprecipitation (co-IP) assays show that GNL3 interaction with γH2AX and RAD51 also increased in response to ETO treatment, which was markedly attenuated by mutating K196 (New supplementary Fig. S3f) in HeLa cells. Similarly, the interaction of GNL3 SIM mutant with BLM and DNA2 was also markedly reduced in response to ETO treatment compared to WT GNL3 (New supplementary Figure S5g) in HeLa cells.

(5) Knockdown of GNL3 led to a marked increase of γH2AX foci and levels, which was rescued by WT GNL3, but not the K196R, E198Q and S141F mutants (New Figs. 8r, 8s; New supplementary Figs. S10f) in HeLa cells.

(6) GNL3 knockdown significantly reduced both RPA-BrdU and RAD51-BrdU PLA signals, which rescued by WT GNL3, but not the SIM mutant, in HeLa cells (New Supplementary Figs S6d-S6g), indicating a critical role of the SIM of GNL3 in DNA end resection and RPA and RAD51 loading onto ssDNA in HeLa cells.

(7) Knockdown of GNL3 led to a marked increase in the levels of γH2AX and p-RPA2, which is rescued by WT GNL3, but not the K196R mutant (New Fig. 8i; New supplementary Fig. S10b) in MDA-MB-231 cells.

In addition, knockdown of GNL3 also induces the γH2AX levels and foci formation in ALT-negative pancreatic ductal adenocarcinoma (PDAC) MiaPaCa-2 cells (below Figure Rev1 for review).

Together, these results demonstrate that the critical role of GNL3 SUMOylation in HR repair is independent of ALT-associated telomere maintenance.

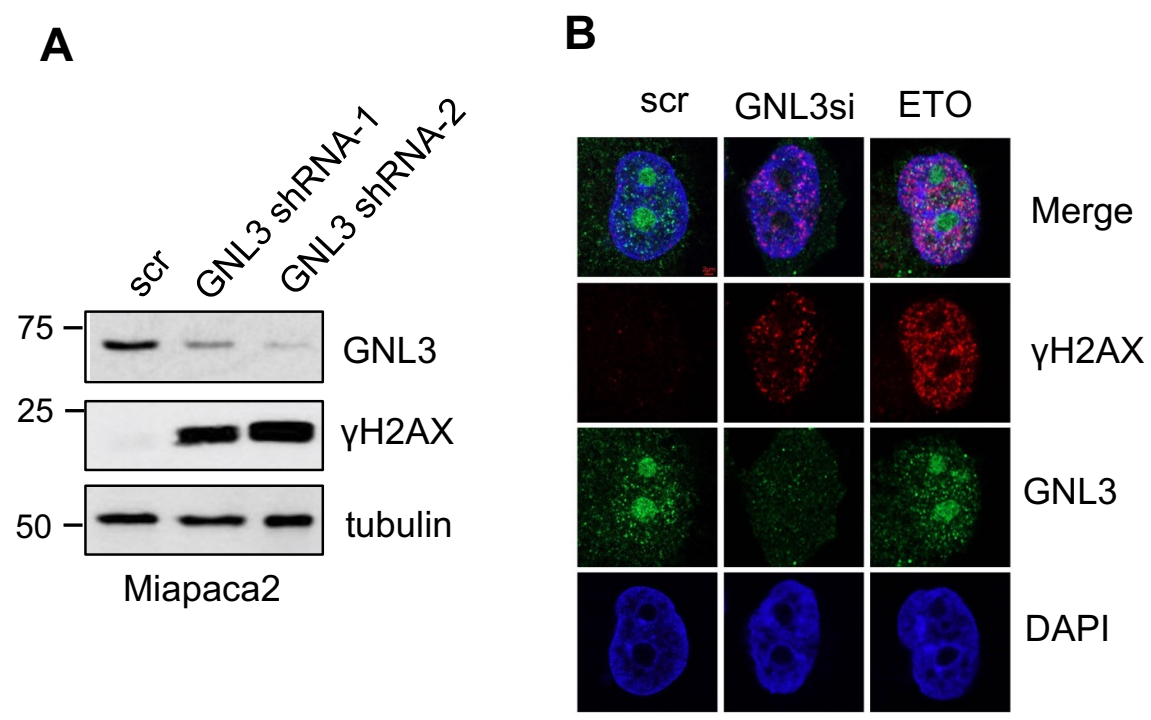

Additional data for review, Figure Rev1. Knockdown of GNL3 induces the lelves and foci formation of γH2AX. (A) MiaPaCa-2 cells were infected with scr or GNL3 lentiviruses were assayed by IB. (B). MiaPaCa-2 cells transfected with scr or GNL3 siRNA or treated with 20 μM ETO for 2 hours were stained with anti-γH2AX and anti-GNL3 antibodies.

2. The authors should provide more conclusive data indicating GNL3 is involved in DNA end resection at DNA damage sites but not involved in replication stress related ALT telomere maintenance. The authors need to have orthogonal approaches to show GNL3 is recruited to DNA damage sites other than PLA assay, such as Laser DNA damage, ChIP at a DSB, chromatin fraction, etc. Most of the experiment were done by overexpression. Some key experiments need to be performed with proteins expressed at their endogenous levels

Our response: We thank the reviewer for raising these critical points. We have provided several additional key evidence showing that GNL3 is recruited to the sites of DNA damage. Please see our response to **reviewer #1 point #4**, including ChIP assays in ER-AsiSI U2OS cells and DI-PLA assays.

To directly examine the role of GNL3 in DNA end resection, we performed the established DNA end resection assays using the ER-AsiSI U2OS cell system, in which 4-hydroxytamoxifen (4-OHT) treatment induces the translocation of AsiSI enzyme from the cytoplasm to the nucleus where it generates site-specific DSBs. Genomic DNAs are subjected to restriction enzyme digestion with BsrG1, which only cleave dsDNA, but not ssDNA. qPCR is then conducted to detect the relative levels of ssDNA generated by DNA end resection (New Fig. 4n), which is resistant to BsrGI digestion. Thus, increased PCR signal reflects increased ssDNA formation and DNA end resection.

Using this assay, we showed that knockdown of GNL3 markedly reduced the relative levels of ssDNA at an AsiSI-induced DSB site on chromosome 1, similar to BRCA1 knockdown (as a positive control) (New Fig. 4o; Supplementary Fig. S5c). Together, these results reveal that GNL3 is critical for efficient DNA end resection at DSBs.

Although U2OS is an ALT-positive cell line, the AsiSI system induces site-specific DSBs at non-telomeric genomic loci, allowing us to assess GNL3 function in HR rather in a telomere-specific ALT context.

In response to the reviewer's concern regarding overexpression, most of our experiments were using siRNA-mediated knockdown of endogenous GNL3 and rescue with exogenous GNL3. We would like to mention that the exogenously expressed GNL3 and its mutants are at levels comparable to

endogenous GNL3 (See Figs. 2e, 8i; New supplementary Fig. S2a, S2i, and S10f; and quantification of total GNL3 levels in New supplementary Fig. S10b).

3. In Fig. 2, the whole figure used three different cell lines without any rational and proper explanation. It needs to provide a justification for using these cell lines and key experiments should be done in multiple different cell lines, to rule out genetic and cell line specific effects.

Our response: Thanks. We performed the assays in different cell lines to exclude potential cell line specific effects. To address this concern and concerns about ALT, we have now only included data from U2OS and HeLa cells, including new data, in revised Figure 2, with rationale of excluding the possible ALT effect (also see our response to above point **#1**).

4. Fig. 3c, IP results showed K196R had less interaction with γH2AX and Rad51. It is possible that IP is pulling down everything and K196R mutant is less chromatin bound. The authors should provide a negative control for IP and should use chromatin fractionation to compare chromatin binding ability of GNL3 WT and K196R mutant.

Our response: We have now included IgG controls to confirm the specificity of the IP and showed γH2AX and Rad51 were specifically pulled down by anti-Flag, but not IgG control, following ETO treatment in both U2OS and HeLa cells (New Supplementary Figs S3e, S3f). In addition, chromatin fractionation assays show that WT and K196R GNL3 exhibit comparable levels in the chromatin fraction (New Supplementary Fig S5i). Thus, the reduced interaction of the K196R mutant with γH2AX and RAD51 is unlikely due to reduced overall chromatin binding.

5. Fig. 3f, Rad51 expression level should be provided in GNL3 knock down. The concentration of Etoposide might need to be less to have reduced RAD51 foci, making quantification and results easier to interpret.

Our response: We thank the reviewer for this helpful suggestion. We have now provided RAD51 protein expression in New supplementary Fig. S2a (and S2i) showing that depletion of GNL3 does not affect total RAD51 protein levels in both U2OS and HeLa cells. We also performed the assays using lower concentration of ETO (5 μM) (New supplementary Figs. S3g, S3h) and showed similar results to 20 μM ETO treatment. Together, these results indicate that GNL3 SUMOylation is critical for RAD51 foci formation without altering its protein expression levels in response to DNA damage

6. U2OS is a known ALT+ cell line, and GNL3 is known to interact with TRF1. In Fig. 2 J and K, the MTT assay and clonogenic assays using U2OS would be hard to pinpoint whether telomere integrity is involved. Perhaps using telomerase+ cell line such us HeLa could address this uncertainty.

Our response: Thanks for the critical suggestion. We have performed the similar assays in HeLa cells and showed that GNL3 knockdown suppressed HeLa cell growth, which can also be rescued by WT GNL3, but not the K196R mutant (New Figs. 2m-2o).

7. **GNL3 is involved in replication stress (PMID: 3796589), so this needs to be addressed to know if γH2AX signals in GNL3 depletion is due to replication stress, DNA damage or a mixture of both. If GNL3 is involved in DNA repair, HR and NHEJ reporter assays need to be done to show this functionally. Depletion of GNL3 might have defect/ delay repair upon drug treatment. Although γH2AX was no affect upon Etoposide treatment in GNL3 depleted cells (Fig. S2a), but it might show defect of repair in later time points compared to siControl. On the other hand, overexpressing GNL3 might repair faster.**

Our response: Thanks for the critical and excellent suggestion. Indeed, it's known that GNL3 is involved in replication stress and knockdown of GNL3 causes replication-stress mediated DNA damage [3, 7, 8]. We have now shown that GNL3 is directly involved in HR repair as evident by HR reporter assays (new supplementary Fig S2j) as well as ChIP and DNA end resection assays using the well-established ER-AsiSI U2OS system (New Figs. 3f, 4o). GNL3 binding to DSBs was also shown by the Tsai group[3]

using ChIP assay in cells transfected with DR-GFP reporter plasmids. Thus, GNL3 plays a critical role in both replication stress and direct HR repair of DSBs.

With regard to the γH2AX foci in GNL3-depleted cells in reponse to ETO treatment, we have now performed ETO washout experiments. As shown in New supplementary Figs. S2d, S2e, knockdown of GNL3 significantly impairs the clearance of ETO-induced γH2AX foci upon ETO removal, further supporting the role for GNL3 in DNA damage repair.

8. **Fig. 3c, GNL3 SUMO-mutant showed less binding to γH2AX. Dose GNL3 have a phospho binding site? The authors should address why less binding of γH2AX would affect DNA end resection and DNA repair.**

Our response: This is a great question. Based on the sequence analysis, it seems that GNL3 does not contain an apparent phospho-binding site such as the BRCT domain seen in MDC1, 53BP1 and BRCA1, etc. As our study points to the key role for GNL3-BLM interaction, the reduced binding to γH2AX for the K196R mutant reflects the reduced binding to BLM and reduced recruitment to DNA damage site. Future studies would address whether GNL3 directly binds to γH2AX.

9. Fig. 3J and Fig. S3, GNL3 is proposed to be involved in resection but no effects on BRCA1/53BP1/BARD1/MDC1 loading but only RAD51 and RPA2. If these cells have less end resection as proposed in the manuscript, it would be expected to have increased 53BP1 foci accumulation at damage sites. The authors should address why GNL3 is involved in DNA end resection but does not affect BRCA1/53BP1/BARD1/MDC1 foci formation.

Our response: We thank the reviewer for this insightful comment. We have now performed 53BP1 foci analyses in GNL3 knockdown cells with ETO treatment. Indeed, GNL3 knockdown leads to an increase in 53BP1 foci formation in response to ETO treatment (New Figs. 3o, 3p).

MDC1, 53BP1, BRCA1, and BARD1 are recruited upstream of DNA end resection and RAD51 loading. Given that GNL3 functions in DNA end resection and likely RAD51 loading, it is not surprising that knockdown of GNL3 does not abolish their recruitment to DNA damage sites. This is also supported by Tsai group shown that knockdown of GNL3 induces BRCA1 foci, but not RAD51[3].

10. Fig. 4, Dose GNL3 affect other resection portions at DNA damage sites such as MRE11, CtIP, Exo1?

Our response: We have performed experiments and showed that GNL3 knockdown does not abolish the foci formation of MRE11, CtIP, EXO1 (New supplementary Figs. S4g-4l). Thus, GNL3 specifically affects BLM-DNA2-mediated long-range DNA end resection.

11. Fig 4a, Does GNL3 IP-MS data also show known GNL3 interaction factors?

Our response: Yes, our IP-MS identified known GNL3 interaction factors, those mainly involved in 60S ribosome biogenesis [9, 10], including nucleophosmin, nucleolin, DDX21, the PeBoW complex (PES1, BOP1, WDR12, and DDX27), EBP2-BRX1 dimer, MAK16, etc (please see our supplementary table for MS data).

12. Fig. 5, Similar to SUMO K196R mutant, SIM mutant might just have less chromatin binding. Authors should provide negative control for IP and should use chromatin fractionation to check chromatin binding ability of GNL3 WT and SIM mutant.

Our response: As suggested, we have now also included IgG controls and showed BLM and DNA2 were specifically pulled down by anti-Flag, but not IgG control, following ETO treatment in both U2OS and HeLa cells (New Supplementary Figs S5f, S5g). In addition, chromatin fractionation assays show that WT GNL3 and the SIM mutant exhibit comparable levels in the chromatin fraction (new Supplementary Fig 5i). Therefore, the reduced interaction of the SIM mutant with BLM and DNA2 is less likely due to overall impaired chromatin binding.

13. The claim of GNL3 involved in DNA end resection by SUMO-SIM interaction with BLM/DNA2 should have additional evidence. More evidence showing that GNL3 SUMO-SIM interaction with

BLM is involved in DNA end resection should be provided. Dose GNL3 SIM-mutant also effects RPA and Rad51 loading on ssDNA?

Our response: We thank the reviewer for this insightful suggestion. First, to further test whether the SUMO-SIM interaction of GNL3 is involved in DNA end resection through BLM, we performed additional GNL3 knockdown and rescue experiments using PLA. As shown in New Supplementary Figs S6a & S6b, mutating the SIM markedly reduced interaction between BLM and GNL3 in cells treated with ETO, further supporting that the SIM motif is required for the association of GNL3 with BLM. Importantly, GNL3-BLM PLA signals largely colocalize with γH2AX foci, indicating that GNL3 interact with BLM at DSB sites (New Supplementary Figs S6c).

Second, we examined RPA and RAD51 loading onto ssDNA by using ssDNA-PLA. Cells were transfected with GNL3 siRNA together with siRNA resistant WT GNL3 or the SIM mutant, followed by BrdU labeling and ssDNA-PLA assays [11] under non-denaturing condition, wherein anti-BrdU only recognizes ssDNA, but not dsDNA. As shown in new Supplementary Figs S6d-S6g, GNL3 knockdown significantly reduced both RPA-BrdU and RAD51-BrdU signals, which rescued by WT GNL3, but not the SIM mutant. Thus, the SIM plays a critical role for GNL3 in DNA end resection and thus proper RPA and RAD51 loading onto ssDNA.

14. Fig. 6K, Depletion of USP36 showed lower level of GNL3, and this is thought to be through post-translational modification. Dose overexpressing UPS36 have higher GNL3 level?

Our response: Overexpression of USP36 does not further increase GNL3 levels in both U2OS and HeLa cells (below Figure Rev2 for review). This is likely due to that GNL3 is a stable protein with a half-life more than 12 hours (See Figs 6J & 6k). The steady-state level of endogenous GNL3 is not or minimally ubiquitinated in cells, which is likely due to USP36-mediated deubiquitination. Further expression of USP36 unlikely further increases GNL3 levels, whereas knockdown of USP36 results in GNL3 decrease (Figs. 6I & 6J) due to impaired deubiquitination.

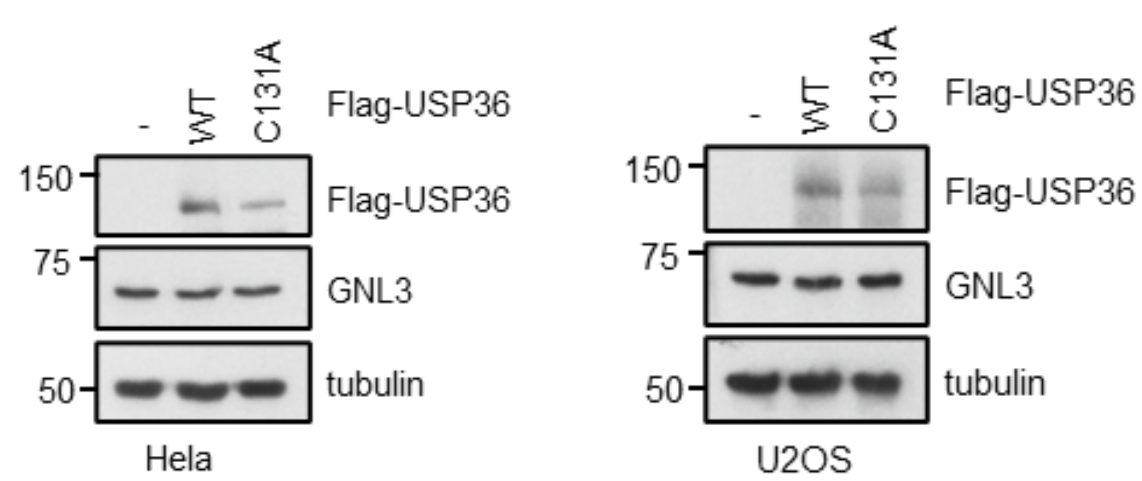

Additional data for review, Figure Rev2. Overexpression of USP36 does not further increase GNL3 levels. HeLa and U2OS cells transfected with control, Flag-USP36 WT or its catalytically inactive C131A mutant were assayed by IB.

15. Fig. 8h, depletion of GNL3 confers drug sensitivity in MDA-MB-231 cells, the authors should include E198Q and S141F and WT constructs for survival assays. Fig. 8, the GNL3 mutation variants E198Q and S141F in U2OS cells, the authors should address if this is due to less resection or is it because of PML recruitment, ie telomere maintenance.

Our response: We thank the reviewer for this excellent suggestion. We have now included rescue experiments in MDA-MB-231 cells using WT GNL3 and the E198Q and S141F mutants. We showed that expression of siRNA resistant WT GNL3, but not the E198Q and S141F mutants rescued MDA-MB-231 cells resistant to Olaparib treatment conferred by knockdown of endogenous GNL3 (New Fig. 8w). This effect is presumably related to impaired HR repair for the mutants, as MDA-MB-231 is also ALT-negative. The results are consistent with those performed in HeLa cells. In addition, we showed that WT GNL3, but not the K196R, E198Q and S141F mutants, abolished endogenous GNL3 knockdown-induced γH2AX foci formation in HeLa cells (New Figs. 8r, 8s). Thus, GNL3 plays a role in DNA end resection independently of its role in ALT-associated telomere maintenance. We hope the reviewer can allow us to further explore the role of GNL3 SUMOylation in telomere maintenance in future studies.

16. GNL3 depletion in MDA-MB-231 cells is shown to lead to PARPi sensitivity but not in BRCA-deficient breast cancer cells. This is hard to reconcile if GNL3 is involved in HR since these

cells are hypomorph so depletion of GNL3 would likely lead to a more severe defect in HR and increase PARPi sensitivity. Can the authors explain this? Also, BRCA-deficient cells should be very sensitive to PARPi compared to BRCA-proficient cells, which is not clear from the data in Figure 8 I and K.

Our response: Our data indeed show that BRCA-deficient MDA-MB-436 cells are more sensitive to PARPi compared to BRCA wild-type MDA-MB-231 cells (compare scr control in Fig. 8m to 8k). MDA-MB-231 cell line is BRCA1 wild type, HR proficient and less sensitive to PARPi. Thus, depletion of GNL3 causing HR defects would sensitize the cells to PARPi (as shown in Fig. 8k). MDA-MB-436 cells harbor BRCA1 gene mutation and are often used to model BRCA1 deficient and behave as BRCA1 function null (no or undetectable BRCA1 protein). The cells are more sensitive to PARPi. As GNL3 also functions in HR and it is expected that GNL3 knockdown has less effect in further sensitizing MDA-MB-436 cells to PARPi (Fig. 8m).

17. How does a DUB also function at a SUMO E3 ligase? Is its DUB activity involved? How do these proteins impact HR repair and endogenous DNA damage (i.e. replication stress, telomeres, DSB repair)?

Our response: These are all excellent questions. We have previously discovered the unexpected SUMO ligase activity of USP36 [12]. USP36 has all SUMO E3 characteristics: binding to Ubc9; binding to SUMO; promoting SUMOylation of multiple proteins, including snoRNP protein, EXOSC10 and Las1L [12-14] in cells; SUMOylating multiple substrates, including Nop58, Nhp2 and PARP1, *in vitro* using recombinant proteins [12, 13]; requiring the SUMO interaction with the backside of Ubc9[12] for SUMO ligase activity, as in the case of PIAS1 and ZNF451 family SUMO ligases[15-18]. To the best of our knowledge, USP36 is the only protein with both deubiquitinating enzyme (DUB) and SUMO ligase dual functions identified so far.

Whether DUB is involved in SUMO ligase activity and HR is a great question. The SUMO ligase activity is located at the N-terminal domain which overlaps with the USP domain. Mutating the DUB catalytic Cys 131 (C131) also abolishes its SUMO ligase activity. However, mutating the DUB catalytic triad residue His 382 to Ala (H382A) abolishes its DUB, but not SUMO E3, activity[12]. Thus, USP36's SUMO ligase activity <u>does not</u> require its DUB activity. Yet, there is an emerging interest in my lab to delineate the mechanism of USP36's SUMO ligase activity and the crosstalk between its DUB and SUMO E3 activity.

We have performed reporter assays and showed that knockdown of USP36 markedly reduced HR activity, but not NHEJ (New supplementary Fig. S8g), supporting its role in DSB HR repair. While it is important to understand USP36 function in replication stress and telomere maintenance, we hope the reviewer can allow us to address these in future studies.

Minor comments

1. In the introduction, "Yet, the majority of cancers are HR-proficient and PARPi resistant". PARPi is less sensitive in BRCA-proficient cells compared to BRCAness, rather than PARPi resistant.

Our response: Thanks. We have made changes as suggested.

2. The formatting should be more consistent. It is a bit distracting throughout the manuscript to have not aligned panels and text. The text should be aligned, and labels should be more consistent.

Our response: Thanks so much for this critical suggestion. We have made changes as suggested.

<u>Response to Reviewer #3:</u>

General comments:

"***While GNL3 SUMOylation is well-supported with data, how much of this SUMOylation contributes to DNA repair is questionable, particularly given that GNL3 and its SUMOylation-defective mutations have no effect on DNA repair and gH2AX foci formation with ETO treatment (supplementary Fig. 2a, b, c, d).”***

Our response: Our data indicate that mutating the SUMO site K196 or the SIM markedly impaired GNL3 function in HR, as evidenced by defects in DSB repair (Figs. 2f, 2g and New supplementary Figs. S2j); the rescue of cell growth inhibition by GNL3 knockdown (Figs. 2h-2j; New Figs. 2m-2o); the rescue of RAD51 foci formation by GNL3 knockdown upon ETO treatment (Figs. 3k, 3l; New Figs. 5f, 5g); the binding to BLM in response to ETO treatment (Fig. 4b, 4h, 4i, 5k-5r); and the rescue of DNA end resection upon GNL3 knockdown (Figs. 4j & 4k).

The K196R and SIM mutants have no effect on γH2AX foci formation upon ETO treatment. This is due to that ETO treatment induces sufficient DSBs to saturate γH2AX foci formation and thus, further GNL3 knockdown or rescue by these mutants does not further increase DNA damages. To address this question more clearly, we performed ETO washout experiments. As shown in New supplementary Figs. S2d, S2e, knockdown of GNL3 significantly impairs the clearance of ETO-treatment-induced γH2AX foci upon ETO removal, further supporting the role for GNL3 in DNA damage repair.

“In addition, the SUMO ligase USP36 and SENP3 all co-localize with GNL3 in the nucleolus, suggesting their main mode of action might be in the nucleolus. The USP36 and SENP3 manipulation results might be the regulation of proteins other than GNL3.” The IP data regarding all interactions between GNL3 and the DDR proteins are not localization-specific, and the co-localization data are based on PLA without evidence that these PLA foci are at DNA damage sites. These may reflect changes in global SUMOylation dynamics in response to nucleolar stress induced by manipulation of GNL3. I'd be more convinced if there were a clear GNL3 co-localization with BLM/DNA at DNA damage sites, and their colocalization at DNA damage sites depends on SUMO/SIM of each protein.”

Our response: These are all great points. GNL3 is a nucleolar-nucleoplasm shuttling protein [1, 2]. The nucleoplasmic GNL3 largely co-localizes with γH2AX following ETO treatment (New supplementary Figs S3c, S3d). GNL3 directly binds to DSB sites as demonstrated by DI-PLA (New Figs. 3c, 3d) and ChIP assays (New Fig. 3e & 3f) (please also see our response to **Reviewer #1 point #4**). We also performed PLA assays and showed that GNL3-BLM PLA signals co-localize with γH2AX foci (New Figs. 4e, 4f). We have already shown that GNL3-BLM interaction in response to DNA damage depends on GNL3 SUMOylation (Figs. 5j-5r). Thus, GNL3 functions in global DNA damage repair in the nucleoplasm. The Tsai group has shown that the nucleolar localization of GNL3 is <u>not</u> required for its function in protecting genome [3]. It will be interesting in future studies to test whether USP36 and SENP3 regulate GNL3 SUMOylation in the nucleolus and affect its function in the nucleoplasm. Of note, our ongoing preliminary study suggest that GNL3 also plays a role in nucleolar DNA damage response (n-DDR) following rDNA damage. Knockdown of GNL3 markedly impaired n-DDR-induced cap formation in response to sgRNA-Cas9 mediated rDNA DSBs (below Figures Rev3 for review). We hope this reviewer allows us to further address this aspect in the ongoing separate study.

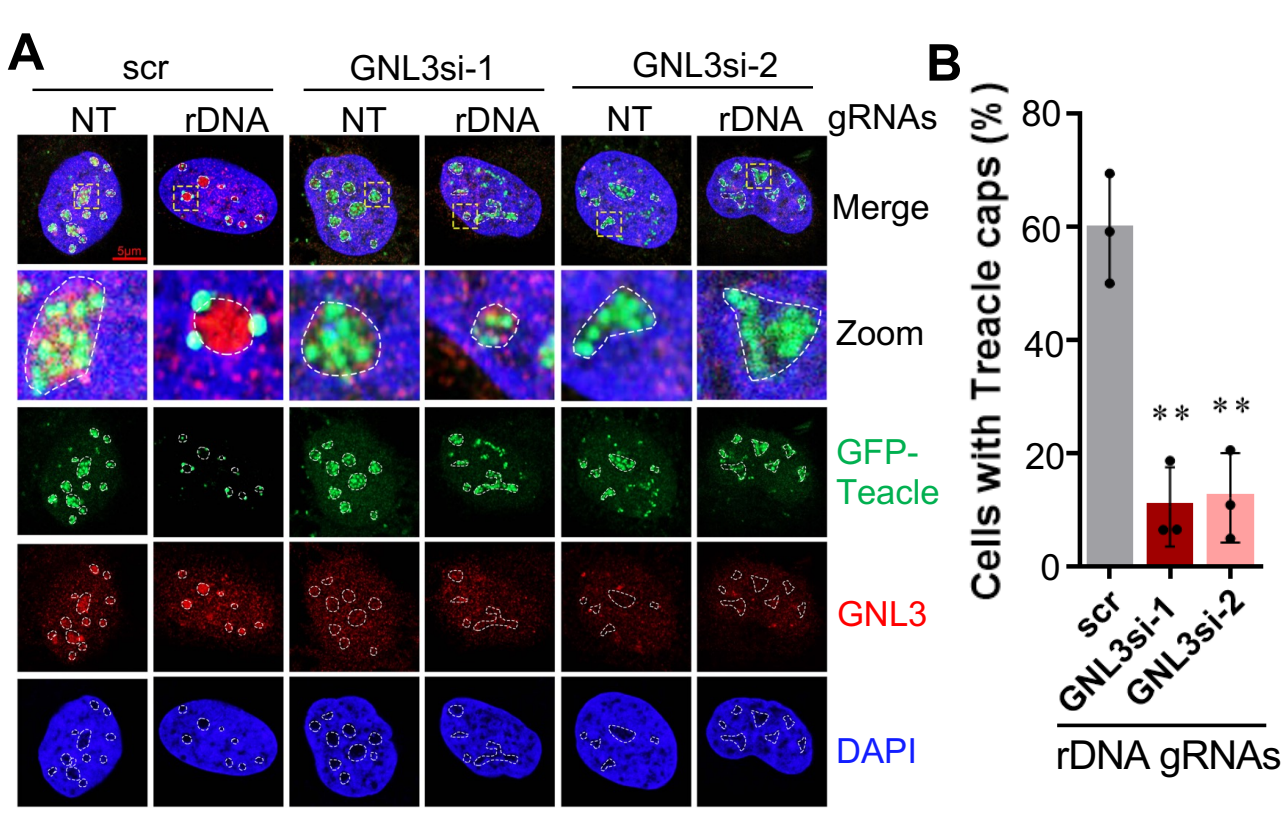

Additional data for review, Figure Rev3. GNL3 is critical for nucleolar Cap formation upon rDNA DSBs. U2OS-Treacle-GFP cells transfected with scr or GNL3 siRNAs together with non-targeting (NT) or rDNA targeting gRNA and Cas9 for 6 hours were assayed by IF. Shown are representative images (A) and quantification (B). Zoomed-in images show details of individual nucleoli. **P<0.01, compared to scr control.

Other comments

1. Knockdown of BLM would help determine whether GNL3 functions upstream or downstream of BLM–DNA2, and whether its role in promoting DNA end resection is fully or only partially dependent on this complex. The authors also propose that GNL3 binds ssDNA generated by MRN-CtIP-mediated short-range resection. Is there additional evidence supporting GNL3 acting at the short-range initiation step, rather than functioning exclusively during long-range BLM-DNA2-mediated resection?

Our response: Thanks for this excellent suggestion. We have now performed BLM knockdown experiments and showed that knockdown of BLM by siRNA significantly reduced the interaction of GNL3 with γH2AX (New supplementary Figs. S7d-S7f), suggesting that GNL3 acts downstream of BLM. Our additional data showed that GNL3 knockdown does not abolish the foci formation of MRE11 and CtIP (New supplementary Figs. S4g-4j), suggesting that GNL3 may specifically affect BLM-DNA2-mediated long-range DNA end resection. Directly confirming whether it affects short-range DNA end resection requires extensive new tools and assays and we hope this reviewer allows us to address this in future studies.

2. The author has identified USP36 as the SUMO ligase for GNL3. The current data rely primarily on overexpression and knockdown experiments, in which do not distinguish between direct enzymatic SUMOylation by USP36 or indirect effects. To confirm USP36 as a SUMO ligase for GNL3, an in vitro enzymatic assay using purified proteins should be done.

Our response: Thanks for the suggestion. We have now performed *in vitro* SUMOylation assays and showed that USP36 SUMOylates GNL3 *in vitro* using purified recombinant proteins (New Fig. 6b).

3. The rationale for investigating GNL3 in the context of SUMOylation and HR repair is not clearly described. More background on prior work on GNL3's role in ribosome biogenesis, p53, and HR, particularly how these roles are differentiated/linked, and what is already known for its role in HR, would be helpful.

Our response: Thanks for the suggestion. We have now incorporated this information in the introduction in the revised manuscript.

4. In Fig. 2C, the DAPI staining shows irregular cell morphology. It would be helpful to provide higher-quality images.

Our response: Thanks. We have provided high-quality images (New supplementary Figs. S3c, New Figure 4e as well as Fig. 2c).

5. In Figure 6h, the WB labels do not correspond correctly to the positions of each band.

Our response: Thanks. We have made the changes.

6. The SUMO E1 inhibitor is listed incorrectly as ML972 in the Methods. In the HR and NHEJ reporter assays Methods, 'transection' should be 'transfection'.

Our response: Thanks so much for correcting the typos. We have made the changes.

7. Some of the sentences are long and confusing.

Our response: Thanks. We have carefully proofread and made the corresponding changes.

Response to Reviewer #4:

I co-reviewed this manuscript with one of the reviewers who provided the listed reports. This is part of the Nature Communications initiative to facilitate training in peer review and to provide appropriate recognition for Early Career Researchers who co-review manuscripts.

Our response: Thanks for reviewing our manuscript. Please see our response to above three reviewers.

References

1. Meng, L., Yasumoto, H. & Tsai, R.Y. Multiple controls regulate nucleostemin partitioning between nucleolus and nucleoplasm. *J Cell Sci* **119**, 5124-5136 (2006).
2. Meng, L., Zhu, Q. & Tsai, R.Y. Nucleolar trafficking of nucleostemin family proteins: common versus protein-specific mechanisms. *Mol Cell Biol* **27**, 8670-8682 (2007).
3. Meng, L. *et al.* Nucleostemin deletion reveals an essential mechanism that maintains the genomic stability of stem and progenitor cells. *Proc Natl Acad Sci U S A* **110**, 11415-11420 (2013).
4. Ouyang, K.J., Yagle, M.K., Matunis, M.J. & Ellis, N.A. BLM SUMOylation regulates ssDNA accumulation at stalled replication forks. *Front Genet* **4**, 167 (2013).
5. Hariharasudhan, G. *et al.* TOPORS-mediated RAD51 SUMOylation facilitates homologous recombination repair. *Nucleic Acids Res* **50**, 1501-1516 (2022).
6. Shima, H. *et al.* Activation of the SUMO modification system is required for the accumulation of RAD51 at sites of DNA damage. *J Cell Sci* **126**, 5284-5292 (2013).
7. Lin, T. *et al.* Nucleostemin reveals a dichotomous nature of genome maintenance in mammary tumor progression. *Oncogene* **38**, 3919-3931 (2019).
8. Lebdy, R. *et al.* The nucleolar protein GNL3 prevents resection of stalled replication forks. *EMBO Rep* **24**, e57585 (2023).
9. Romanova, L. *et al.* Critical role of nucleostemin in pre-rRNA processing. *J Biol Chem* **284**, 4968-4977 (2009).
10. Thome, C.C. *et al.* Distinct steps of nuclear maturation of human pre-60S complexes require the activity of GTPases including GNL3L. *Nucleic Acids Res* **54** (2026).
11. Yang, Y., Li, Y., Sun, X.X. & Dai, M.S. ssDNA-PLA, a proximity ligation assay to interrogate DNA damage response proteins involved in homologous recombination. *Biol Methods Protoc* **11**, bpag003 (2026).
12. Ryu, H. *et al.* The deubiquitinase USP36 promotes snoRNP group SUMOylation and is essential for ribosome biogenesis. *EMBO Rep* **22**, e50684 (2021).
13. Chen, Y. *et al.* The ubiquitin-specific protease USP36 SUMOylates EXOSC10 and promotes the nucleolar RNA exosome function in rRNA processing. *Nucleic Acids Res* (2023).
14. Li, Y., Yang, Y., Sears, R.C., Dai, M.S. & Sun, X.X. USP36 SUMOylates Las1L and Promotes Its Function in Pre-Ribosomal RNA ITS2 Processing. *Cancer Res Commun* **4**, 2835-2845 (2024).
15. Cappadocia, L., Pichler, A. & Lima, C.D. Structural basis for catalytic activation by the human ZNF451 SUMO E3 ligase. *Nat Struct Mol Biol* **22**, 968-975 (2015).
16. Eisenhardt, N. *et al.* A new vertebrate SUMO enzyme family reveals insights into SUMO-chain assembly. *Nat Struct Mol Biol* **22**, 959-967 (2015).
17. Eisenhardt, N., Ilic, D., Nagamalleswari, E. & Pichler, A. Biochemical characterization of SUMO-conjugating enzymes by in vitro sumoylation assays. *Methods Enzymol* **618**, 167-185 (2019).
18. Koidl, S. *et al.* The SUMO2/3 specific E3 ligase ZNF451-1 regulates PML stability. *Int J Biochem Cell Biol* **79**, 478-487 (2016).

Point-to point Responses to reviewers' comments:

Reviewer #1:
The authors have carefully responded to and addressed all the reviewers' comments, and the quality of the manuscript has significantly improved. I have no further substantive comments.

Reviewer #1:
The authors have done extensive revisions that further support a role for GLN3 and its SUMOylation in DNA end resection and DNA repair. The authors have now addressed all of this reviewer concerns prior to publication.

Reviewer #3:
The authors have addressed all of my concerns.

Reviewer #4:
I co-reviewed this manuscript with one of the reviewers who provided the listed reports. This is part of the Nature Communications initiative to facilitate training in peer review and to provide appropriate recognition for Early Career Researchers who co-review manuscripts.

Our response: We greatly appreciate all the reviewer for all their constructive suggestions throughout the review process, which have significantly improved our study. We thank all the reviewers for their support of the publication of our work in Nat Communications.
