# Supplementary material for: GNL3 SUMOylation is essential for DNA double-strand break repair by homologous recombination
Source: Nat Commun. 2026 Aug 24;17:10122. doi: 10.1038/s41467-026-77013-1 (PMC13598120; doi:10.1038/s41467-026-77013-1)
Supplement: Supplementary file 3 [file 41467_2026_77013_MOESM3_ESM.pdf]

nature portfolio

Corresponding author(s): Mu-Shui Dai

Last updated by author(s): 6/18/2026

Reporting Summary

Nature Portfolio wishes to improve the reproducibility of the work that we publish. This form provides structure for consistency and transparency in reporting. For further information on Nature Portfolio policies, see our Editorial Policies and the Editorial Policy Checklist.

Statistics

For all statistical analyses, confirm that the following items are present in the figure legend, table legend, main text, or Methods section.

n/a	Confirmed	
[ ]	[x]	The exact sample size (n) for each experimental group/condition, given as a discrete number and unit of measurement
[ ]	[x]	A statement on whether measurements were taken from distinct samples or whether the same sample was measured repeatedly
[ ]	[x]	The statistical test(s) used AND whether they are one- or two-sided<br>*Only common tests should be described solely by name; describe more complex techniques in the Methods section.*
[x]	[ ]	A description of all covariates tested
[ ]	[x]	A description of any assumptions or corrections, such as tests of normality and adjustment for multiple comparisons
[ ]	[x]	A full description of the statistical parameters including central tendency (e.g. means) or other basic estimates (e.g. regression coefficient) AND variation (e.g. standard deviation) or associated estimates of uncertainty (e.g. confidence intervals)
[ ]	[x]	For null hypothesis testing, the test statistic (e.g. F, t, r) with confidence intervals, effect sizes, degrees of freedom and P value noted<br>*Give P values as exact values whenever suitable.*
[x]	[ ]	For Bayesian analysis, information on the choice of priors and Markov chain Monte Carlo settings
[x]	[ ]	For hierarchical and complex designs, identification of the appropriate level for tests and full reporting of outcomes
[x]	[ ]	Estimates of effect sizes (e.g. Cohen's d, Pearson's r), indicating how they were calculated

Our web collection on statistics for biologists contains articles on many of the points above.

Software and code

Policy information about availability of computer code

Data collection	Microscopy images were acquired using a Zeiss LSM980 microscope, using a ZEISS Microscopy Software
Data analysis	Prism v10 were used for statistical analysis. Imaging analysis of DNA damage foci and PLA signal was performed using ImageJ v1.54 software.

For manuscripts utilizing custom algorithms or software that are central to the research but not yet described in published literature, software must be made available to editors and reviewers. We strongly encourage code deposition in a community repository (e.g. GitHub). See the Nature Portfolio guidelines for submitting code & software for further information.

Data

Policy information about availability of data

All manuscripts must include a data availability statement. This statement should provide the following information, where applicable:

- Accession codes, unique identifiers, or web links for publicly available datasets
- A description of any restrictions on data availability
- For clinical datasets or third party data, please ensure that the statement adheres to our policy

The mass spectrometry proteomics data generated in this study have been deposited to the ProteomeXchange Consortium via the PRIDE partner repository with the dataset identifier PXD068465 (https://www.ebi.ac.uk/pride/archive/projects/PXD068465). GNL3 expression data for breast cancer were obtained from TCGA via the Genomic Data Commons Data Portal (https://portal.gdc.cancer.gov/). GNL3 protein expression data for breast cancers were obtained from CPTAC via UALCAN (https://ualcan.path.uab.edu/analysis-prot.html). Survival data were obtained from Kaplan–Meier plotter (http:// kmplot.com/analysis/). GNL3 variants in breast

cancers were obtained from cBioportal (https://www.cbioportal.org/).

All data generated or analyzed in this study are included in this article and its Supplementary Information files. All relevant raw data from figures generated in this study have been provided in the Source Data file. Source data are provided with this paper.

Research involving human participants, their data, or biological material

Policy information about studies with human participants or human data. See also policy information about sex, gender (identity/presentation), and sexual orientation and race, ethnicity and racism.

Reporting on sex and gender	Not applicable
Reporting on race, ethnicity, or other socially relevant groupings	Not applicable
Population characteristics	Not applicable
Recruitment	Not applicable
Ethics oversight	Not applicable

Note that full information on the approval of the study protocol must also be provided in the manuscript.

Field-specific reporting

Please select the one below that is the best fit for your research. If you are not sure, read the appropriate sections before making your selection.

[x] Life sciences   [ ] Behavioural & social sciences   [ ] Ecological, evolutionary & environmental sciences

For a reference copy of the document with all sections, see nature.com/documents/nr-reporting-summary-flat.pdf

Life sciences study design

All studies must disclose on these points even when the disclosure is negative.

Sample size	Sample sizes were chosen according to common practice in the field.
Data exclusions	No data were excluded from the analysis.
Replication	All experiments were performed at least three independent biological replicates. the exact number of repeats of each experiments are described in figure legends and methods.
Randomization	For in vitro cell culture experiments, cells were randomly allocated to control and experimental groups.
Blinding	Not applicable

Reporting for specific materials, systems and methods

We require information from authors about some types of materials, experimental systems and methods used in many studies. Here, indicate whether each material, system or method listed is relevant to your study. If you are not sure if a list item applies to your research, read the appropriate section before selecting a response.

Materials & experimental systems

n/a	Involved in the study	
[ ]	[x]	Antibodies
[ ]	[x]	Eukaryotic cell lines
[x]	[ ]	Palaeontology and archaeology
[x]	[ ]	Animals and other organisms
[x]	[ ]	Clinical data
[x]	[ ]	Dual use research of concern
[x]	[ ]	Plants

Methods

n/a	Involved in the study	
[x]	[ ]	ChIP-seq
[ ]	[x]	Flow cytometry
[x]	[ ]	MRI-based neuroimaging

Antibodies

Antibodies used	Anti-Flag (IF 1:1000; PLA 1:1000; IB 1:2000 Sigma F3165<br>Anti-V5 (IB 1:3000) Life technologies R960-25

	Anti-tubulin (IB 1:10000) Proteintech 66240-1-Ig<br>Anti-γH2AX (IF 1:500; PLA 1:500; IB 1:4000) Millipore 05-636-25<br>Anti-GNL3 (IF 1:200; PLA 1:200) Santa Cruz sc-166460<br>Anti-GNL3 (IF 1:400; PLA 1:400; IB 1:1000) Proteintech 15060-AP<br>Anti-GNL3 (IB 1:2000) Dai et al N/A<br>Anti-RAD51 (IF 1:1000) Abcam ab133534<br>Anti-RAD51 (IB 1:2000) Proteintech No1496-AP<br>Anti-GAPDH (IB 1:10000) Proteintech 60004-1-1g<br>Anti-Nop58 (IF 1:400) Bethyl A302-719A<br>Anti-SENP3 (IF 1:400; IB 1:1000) Cell signaling technology 5591S<br>Anti-BRCA1 (IF 1:300) Santa Cruz sc5654<br>Anti-BARD1 (IF 1:200) Bethyl A300-263A<br>Anti-53BP1 (IF 1:200) Millipore 630916<br>Anti-MDC1 (IF 1:500) Bethyl A300-053A<br>Anti-BLM (IF 1:50; IB 1:500) Santa Cruz sc-365753<br>Anti-BLM (PLA 1:200; IB 1:1000) Invitrogen PA5-77880<br>Anti-RPA2 phospho S33 (IF 1:1000; IB 1:5000) Abcam ab21187<br>anti-DNA2 (IB 1:5000) Proteintech 18727-1-AP<br>Anti-EXO1 (IF 1:1000) Invitrogen AB-2546534<br>Anti-H2B (IB 1:5000) Millipore 07-371<br>Anti-RPL5 (IB 1:2000) Dai et al N/A<br>Anti-RPL30 (IB 1:500) Santa Cruz sc-98106<br>Anti-B23 (IB 1:5000) Zymed 32-5200<br>Anti-SP1 (IB 1:2000) Millipore 07-645<br>Anti-BrdU (IF 1:100; PLA 1:100) Roche 11170376001<br>Anti-SUMO2/3 (IB 1:4000) Dr. Yoshiaki Azuma N/A<br>Anti-SUMO2/3 (IB 1:2000) abcam ab3742<br>Anti-USP36 (IB 1:1000) Dr. Masayuki Komada N/A<br>Anti-USP36 (IB 1:1000) Proteintech 14783-1-AP<br>Anti-Biotin (PLA 1:50) Abcam ab53494<br>Alexa Fluor 488 goat anti-mouse IgG (IF 1:400) Invitrogen A11001<br>Alexa Fluor 555 goat anti-mouse IgG (IF 1:400) Invitrogen A21422<br>Alexa Fluor 488 goat anti-rabbit IgG (IF 1:400) Invitrogen O6381<br>Alexa Fluor 555 goat anti-rabbit IgG (IF 1:400) Invitrogen A21428
Validation	The antibodies purchased from the best commercial vendors were selected based on relevant literatures and validated by the individual vendors as shown on the websites of the manufacturer and in the cited articles. We performed the validation using western blot, IP, and IF staining.<br><br>House-made antibodies including anti-USP36 and anti-GNL3 were extensively verified by IB and siRNA knockdown experiments in our published papers (PMC4378440; PMC2447154; PMC8183414; PMC10164564; PMC11523043)

Eukaryotic cell lines

Policy information about cell lines and Sex and Gender in Research

Cell line source(s)	U2OS, HEK293, HeLa, H1299, MDA-MB-231 and MDA-MB-436 were derived from the ATCC. ER-AsiSI U2OS cells were provided by Dr. Gaëlle Legube (University of Toulouse, France).
Authentication	Cell lines have been authenticated via STR profiling by ATCC. In this study, authentication of cell lines was performed via morphology assessment.
Mycoplasma contamination	All cell lines have been tested to be free of mycoplasma
Commonly misidentified lines<br>(See ICLAC register)	Not applicable

Plants

Seed stocks	No applicable
Novel plant genotypes	No applicable
Authentication	No applicable

Flow Cytometry

Plots

Confirm that:

- [x] The axis labels state the marker and fluorochrome used (e.g. CD4-FITC).
- [x] The axis scales are clearly visible. Include numbers along axes only for bottom left plot of group (a 'group' is an analysis of identical markers).
- [x] All plots are contour plots with outliers or pseudocolor plots.
- [x] A numerical value for number of cells or percentage (with statistics) is provided.

Methodology

Sample preparation	U2OS cells were transfected with DR-GFP or PimEJ5GFP plasmids together with pCherry and pCBAScel plasmids using using TransIT-LT1 reagents (Mirus Bio Corporation) reagetns. Six hours post-transfection, the cells were replaced with free medium and transfected with scr, GNL3si, USP36 si, or SENP3 siRNA using Lipofectamine 2000 (Life Technologies) reagents . 48 hours after siRNA transfection, the cells were trypsinized, washed with 1xPBS and resuspended in o.5 ml PBS. the percentage of GFP and mCherry double positive cells was determined by flow cytometry.
Instrument	Data collection was performed using a BD LASR II analyzer (BD Bioscience)
Software	BD FACSDiva software (BD Bioscience) and FlowJo (FlowJo.com)
Cell population abundance	All the transfection efficiency was nomalized with the expression of mCherry. Cells transfected with pCBAScel, pCherry and DR-GFP together with scr had GFP/mCherry double positivity of approximately 3%, respectively. Cells transfected with pCBAScel, pCherry and EJF-GFP together with scr or SENP3 siRNA had GFP/mCherry double positivity of approximately 6%, respectively.
Gating strategy	Cells were gated first with FSC and SSC to determine live and single cells. Cells were then gated with mCherry and GFP positive cells. The expression of mCherry was used for the normalization of transfection efficiency.

- [x] Tick this box to confirm that a figure exemplifying the gating strategy is provided in the Supplementary Information.
