# Supplementary material for: GNL3 SUMOylation is essential for DNA double-strand break repair by homologous recombination
Source: Nat Commun. 2026 Aug 24;17:10122. doi: 10.1038/s41467-026-77013-1 (PMC13598120; doi:10.1038/s41467-026-77013-1)
Supplement: Supplementary file 4 [file 41467_2026_77013_MOESM4_ESM.pdf]

Description of Additional Supplementary Files

File Name: Supplementary Data 1

Description: Mass spectrometry of GNL3-associated interactome

File Name: Supplementary Data 2

Description: List of plasmids used in this study

File Name: Supplementary Data 3

Description: List of primers used in this study

File Name: Supplementary Data 4

Description: List of antibodies used in this study
